# Comparator-Dependent Safety Signals for Incident Systemic Autoimmune Rheumatic Diseases After mRNA COVID-19 Vaccination: A Systematic Review

**DOI:** 10.3390/vaccines14080706

**Published:** 2026-08-17

**Authors:** Larisa Pinte, Paul Balanescu, Alina Dima, Ana-Maria Mandescu, Mirela-Emanuela Simion-Stanciu, Andra-Cristiana Dumitru, Cristian Baicus

**Affiliations:** 1 Faculty of Medicine, Carol Davila University of Medicine and Pharmacy, 050474 Bucharest, Romania; paul.balanescu@umfcd.ro (P.B.); alina.dima@umfcd.ro (A.D.); andra-cristiana.dumitru@drd.umfcd.ro (A.-C.D.); cristian.baicus@umfcd.ro (C.B.); 2 Department of Internal Medicine, Department of Rheumatology, Colentina Clinical Hospital, 020125 Bucharest, Romania; mirela-emanuela.stanciu@rez.umfcd.ro; 3 Cochrane Romania Affiliate Centre, Carol Davila University of Medicine and Pharmacy, 050474 Bucharest, Romania; 4 Neurology Department, Colentina Clinical Hospital, 020125 Bucharest, Romania; ana-maria.mandescu@rez.umfcd.ro

**Keywords:** COVID-19, mRNA vaccine, immune disease, safety, systematic review

## Abstract

Background: Pharmacovigilance systems have flagged possible associations between mRNA COVID-19 vaccination and systemic autoimmune inflammatory rheumatic diseases (AIRDs), generating uncertainty for rheumatologists counselling patients. As the first population-scale deployment of an mRNA vaccine platform, COVID-19 vaccination provides a unique setting to examine whether autoimmune safety signals detected in spontaneous reporting systems correspond to measurable disease risk. We synthesised pharmacovigilance and population-based evidence on incident EULAR-defined systemic AIRDs after mRNA COVID-19 vaccination and assessed whether disproportionality signals were corroborated by analytical studies. Methods: We conducted a PRISMA 2020-compliant systematic review searching MEDLINE, Web of Science, Scopus, Embase, and the Cochrane Library from 2019 to April 2026, supplemented by medRxiv and trial registries. Eligible studies included pharmacovigilance disproportionality analyses and analytical studies, including cohorts and randomised controlled trials, evaluating BNT162b2 or mRNA-1273 in adults without known pre-existing autoimmune disease. Risk of bias was assessed using READUS-PV, ROBINS-I, and RoB 2. Meta-analysis was not performed because of substantial heterogeneity. Results: Fourteen studies were included: seven pharmacovigilance studies and seven analytical studies. Disproportionality analyses suggested increased reporting of selected AIRDs, most consistently polymyalgia rheumatica and giant cell arteritis, mainly when all other adverse-event reports served as comparators. These signals were largely neutral when influenza vaccines were the reference. Across analytical studies, associations were inconsistent; modest increases in systemic lupus erythematosus appeared only in selected analyses. Long-term evidence was scarce: only four studies, from three countries (South Korea, Israel, and Norway), followed participants for up to approximately one year, and three of these reported at least one positive association—systemic lupus erythematosus, post-booster rheumatoid arthritis, and polymyalgia rheumatica in older adults—whereas studies restricted to risk windows of three months or less reported no increase. Conclusions: The available evidence does not indicate a consistent increase in incident systemic AIRDs after mRNA COVID-19 vaccination. Although pharmacovigilance studies identified comparator-dependent signals for selected diseases, particularly polymyalgia rheumatica and giant cell arteritis, these findings were generally not confirmed in comparative population-based studies and should be considered hypothesis-generating. Delayed-onset disease remains poorly characterised, and studies with at least one year of follow-up are needed.

## 1. Introduction

The rollout of messenger ribonucleic acid (mRNA) Coronavirus Disease 2019 (COVID-19) vaccines enabled the rapid and effective control of the Severe Acute Respiratory Syndrome Coronavirus 2 (SARS-CoV-2) pandemic [1], but their large-scale deployment and first widespread clinical use prompted sustained interest in their immunologic safety profile. Although mRNA platforms had been explored for decades [2], COVID-19 marked their first large-scale application. Early post-marketing reports of myocarditis, pericarditis, and various immune-mediated events raised concerns about the potential risk of new-onset inflammatory rheumatic diseases occurrence [3,4].

Vaccine safety has been monitored globally through major pharmacovigilance (PV) systems, including VigiBase (the World Health Organization global database of individual case safety reports), the Vaccine Adverse Event Reporting System (VAERS), and EudraVigilance (the European Medicines Agency database for suspected adverse drug reactions), complemented by national registries [5]. These systems can detect disproportionate reporting patterns but do not provide incidence estimates or causal inference; comparative risk assessment thus relies on analytical population-based studies, particularly cohort designs. In this systematic review, we treat disproportionality analyses as hypothesis-generating (signals of disproportionate reporting) and analytical population-based studies as hypothesis-testing (comparative risk estimation). We therefore interpret PV signals primarily by their comparator dependence and by whether they are corroborated in analytical designs.

At the same time, the unprecedented adoption of mRNA platforms has accelerated their development for other infectious diseases beyond SARS-CoV-2, as well as oncology or rare disorders [6]. Notably, individuals with autoimmune disease were commonly excluded from mRNA therapeutic trials [6], underscoring the importance of real-world vaccine data for identifying potential immune-mediated risks in the general population.

Several case reports and small series have described new-onset autoimmune inflammatory rheumatic diseases (AIRDs) occurring shortly after mRNA COVID-19 vaccination, prompting interest in potential vaccine-related immune effects [7,8,9], but these designs cannot quantify risk. Robust population-level data remains comparatively limited, and existing reviews have largely focused on case-based evidence or mixed autoimmune outcomes, leaving uncertainty regarding population-level risk for European Alliance of Associations for Rheumatology (EULAR)-defined AIRDs after mRNA vaccination.

We conducted a systematic review integrating pharmacovigilance signal detection evidence with population-based cohort and trial data, with a specific focus on whether disproportionality signals for incident AIRDs are corroborated by analytical designs—a question relevant not only to COVID-19 vaccine safety, but to the interpretation of safety signals as mRNA platforms expand to new indications. To our knowledge, no previous review has systematically compared pharmacovigilance disproportionality findings with analytical cohort evidence for EULAR-defined systemic AIRDs following mRNA vaccination.

## 2. Materials and Methods

### 2.1. Protocol and Reporting

This systematic review followed the Cochrane Handbook for Systematic Reviews of Interventions [10] and was reported in accordance with the Preferred Reporting Items for Systematic Reviews and Meta-Analyses (PRISMA) 2020 statement [11].

The study protocol was prospectively registered in the International Prospective Register of Systematic Reviews (PROSPERO) (CRD42024541705).

### 2.2. Information Sources and Search Strategy

A comprehensive search for studies published from 2019 onwards was conducted in five relevant medical databases: MEDLINE (via PubMed), Web of Science Core Collection, Scopus, Embase (Elsevier), and Cochrane Library on 4 April 2024, with an updated search performed on 25 April 2026. Search terms covered mRNA, COVID-19 vaccines, immunity, and adverse events; full strategies and database-specific restrictions are provided in the Supplementary Files, Section S1 (Table S1).

In addition, supplemented searches on preprint servers and clinical trials, specifically medRxiv, ClinicalTrials.gov, and the WHO International Clinical Trials Registry Platform (ICTRP), were performed on 25 April 2026 in order to identify unpublished or ongoing studies. The reference lists of included studies and relevant reviews were also screened.

### 2.3. Eligibility Criteria

We included studies evaluating mRNA COVID-19 vaccines in the general adult population without known pre-existing autoimmune disease. Eligible comparators differed by study design and included no vaccination/no intervention, placebo, historical background rates including literature-derived rates, and head-to-head comparisons of BNT162b2 and mRNA-1273 when effect estimates were reported. For analytical studies, we required an incident AIRD definition that excluded participants with pre-existing autoimmune disease at baseline (e.g., diagnosis-code washout/look-back period or explicit registry-based exclusion, as reported). Because pharmacovigilance reports typically do not capture pre-existing autoimmune disease reliably, we did not apply this criterion to pharmacovigilance studies and interpreted their findings accordingly. Outcomes were restricted to new-onset AIRD, selected based on the EULAR reference list [12] (Supplementary Section S2), to improve clinical specificity and comparability across administrative datasets. We focused on EULAR-defined systemic AIRDs because they represent clinically serious, systemic immune-mediated outcomes that are relevant to internal medicine and rheumatology, have established classification frameworks, and are sufficiently specific to allow meaningful comparison between pharmacovigilance signals and population-based diagnostic data. We extracted each study’s operational outcome definitions and have summarised them in the study characteristics table.

Studies reporting poorly defined autoimmune conditions or non-specific musculoskeletal symptoms, case reports, case series, reviews, and editorials were excluded. Eligible designs were pharmacovigilance analyses, randomised controlled trial safety analyses, and non-randomised intervention studies. No restrictions were applied regarding language or setting. The full PICO (Population, Intervention, Comparator, and Outcomes) framework is detailed in Supplementary Section S3 (Table S2).

### 2.4. Data Collection and Analysis

#### 2.4.1. Article Selection and Management

Pairs of reviewers independently screened titles, abstracts, and, subsequently, full texts using Rayyan (Rayyan Systems Inc., Doha, Qatar) [13], with disagreements resolved by consensus or adjudication. Screening procedures were piloted on a subset of records (50 for title and abstract screening and 5 for full-text screening).

Deduplication and reference management were performed in EndNote^®^ 20 (Clarivate Analytics, Philadelphia, PA, USA) [14]. No automation tools were used.

#### 2.4.2. Data Extraction

Two reviewers (PB, MS) independently extracted data, and a third reviewer (LP) verified all extractions for both completeness and accuracy. The data extraction followed STROBE (Strengthening the Reporting of Observational Studies in Epidemiology) guidance [15] and included study characteristics (design, setting, period, funding), participant characteristics, vaccine exposure (type, dose, regimen), comparator, and the timeframe between vaccination and disease diagnosis. Outcome measures included the incidence rates of patients with new-onset AIRDs, along with the effect estimates reported (hazard ratios, incidence rate ratios, reporting odds ratios), stratified by disease type and, where applicable, population subgroups.

For pharmacovigilance studies, additional variables included database source, exposure definition, comparator, disproportionality metrics, and signal thresholds.

#### 2.4.3. Risk of Bias Assessment

The reporting quality of the pharmacovigilance studies was assessed using the READUS-PV (Reporting of Evidence-based Disproportionality Analyses for Pharmacovigilance) framework, as described by Fusaroli et al. (2024) [16], with each included criterion scored from 0 to 2.

Cohort studies were evaluated using ROBINS-I (Risk Of Bias In Non-randomized Studies-of Interventions) version 2 [17] across seven domains: confounding, participant selection, classification of interventions, deviations from intended interventions, missing data, outcome measurement, and selection of reported results.

Randomised controlled trials (RCTs) were assessed using RoB2 (Risk of Bias 2) across the five domains [18].

All assessments were performed independently in duplicate, with disagreements resolved by consensus and third-party review.

#### 2.4.4. Data Synthesis and Analysis

Given the marked heterogeneity in study design, exposure definition, comparators, and outcome measures, performing a meta-analysis was not feasible. A qualitative synthesis was therefore undertaken. In cohort studies, incidence measures (HR, IRR) were summarised in tables and stratified by vaccine type, dose, and the specific AIRD considered. Subgroup analyses included age, gender, prior SARS-CoV-2 infection, vaccination regimen (homologous vs. heterologous), and booster dose status.

Spontaneous reporting systems cannot provide population incidence estimates, because event capture is incomplete and the population generating the reports is not reliably defined. They do, however, yield reporting counts, reporting rates where doses distributed are known, and disproportionality measures, which are informative for comparing signals across products and for hypothesis generation.

In disproportionality analyses, the signal definitions followed those reported in the primary studies; where unspecified, standard disproportionality thresholds were applied. A signal refers to an unexpectedly high number of adverse event reports associated with a specific drug when compared to other reports in the pharmacovigilance database. Safety signals can be detected using either frequentist or Bayesian statistical approaches [19].

Reporting odds ratio (ROR) and the proportional reporting ratio (PRR) are commonly used statistical approaches, whereas Bayesian methods include the Bayes geometric mean (EBGM) and the information component (IC). For PRR, widely accepted signal detection thresholds reported in the literature are PRR ≥ 2 and χ^2^ ≥ 4. For ROR, a signal is typically defined as a lower 95% confidence interval (CI) bound greater than 1. For IC, IC025 (the lower 95% CI bound) > 0, while for EBGM, EB05 (the lower one-sided 95% CI bound) ≥ 2 [20].

## 3. Results

### 3.1. Results of the Search

**Figure 1:**
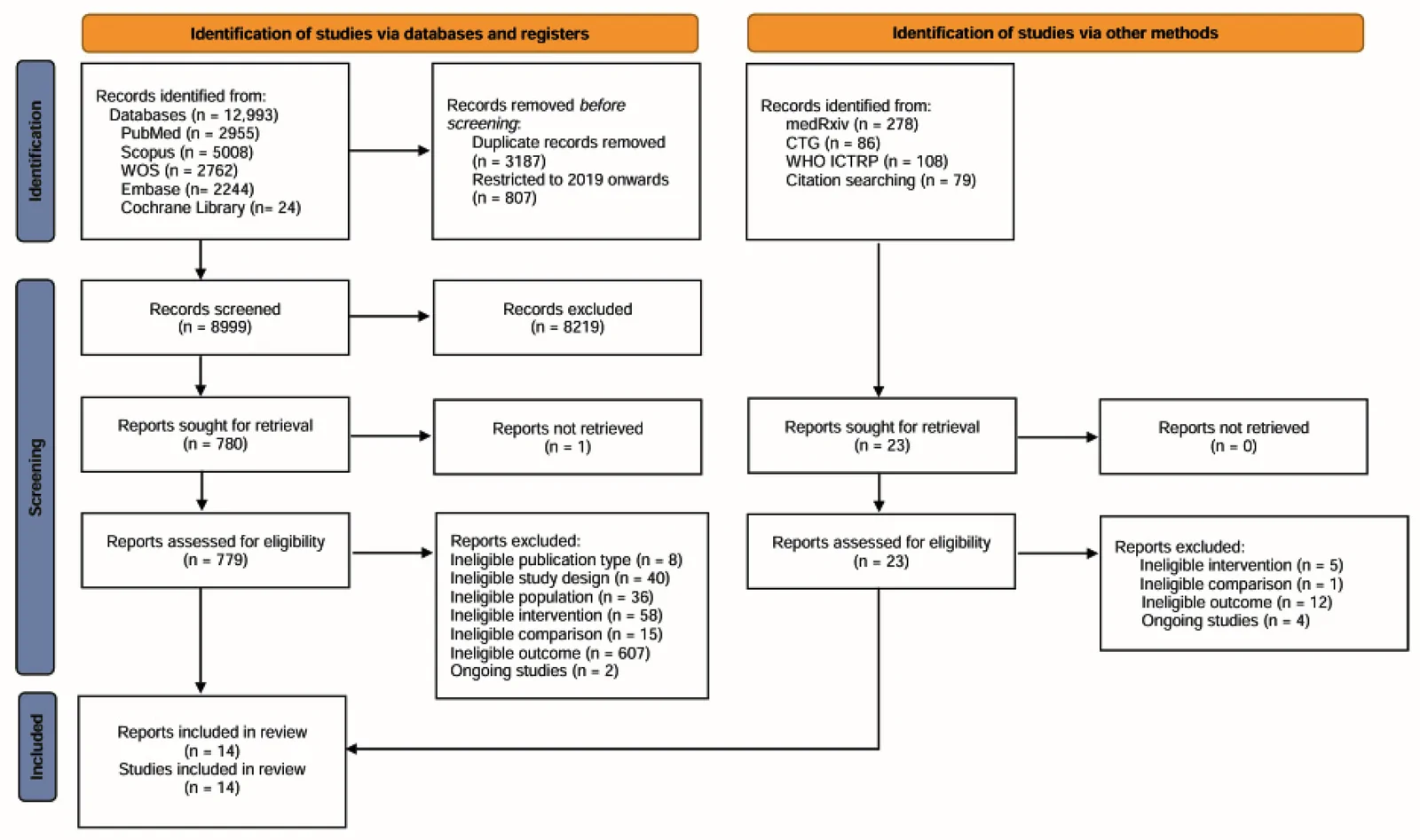
PRISMA 2020 flowchart diagram of study identification, screening, eligibility, and inclusion.

The literature search identified 12,993 records. After the removal of duplicates and screening, 14 studies met the eligibility criteria following full-text review and were included in the analysis (Supplementary Section S9). The study selection process is detailed in the PRISMA flowchart (Figure 1).

The included studies comprised seven pharmacovigilance disproportionality analyses [21,22,23,24,25,26,27], six non-randomised observational cohort studies [28,29,30,31,32,33], and one randomised controlled trial safety analysis [34].

The characteristics of the included studies are provided in the Supplementary Materials (Section S4; Tables S3 and S4), with confounding variables considered across studies as summarised in Table S5. Outcomes were new-onset AIRDs as defined by the EULAR reference list; the full outcome set is summarised in Table S6, with detailed results presented in Supplementary Section S5 (pharmacovigilance) and Section S6 (cohort/RCT evidence). The Supplementary Materials lists all studies excluded at the full-text stage (Supplementary Section S10) and the corresponding reasons for exclusion, and provides a table with the ongoing trials (Supplementary Section S8, Table S20).

### 3.2. Pharmacovigilance (Disproportionality Analyses)

Seven disproportionality studies from the international and national pharmacovigilance databases, namely, VigiBase (global WHO, data up to 2022/2023) [23,24,26], EudraVigilance (Europe, 2021–2023) [21], JADER (Japan, 2004–2022) [25], France pharmacovigilance database (data up to 2022) [22], and VAERS (the US national passive vaccine safety surveillance system, up to March 2025) [27], were included (Table 1).

Overall mRNA vaccines (BNT162b2 and mRNA-1273) were evaluated using different reference sets for disproportionality analyses, including all other adverse event reports for any medicinal product (‘all other drugs’) (four studies) [23,24,25,26], all other COVID-19 vaccines (one study) [21], all other vaccines (one study) [27], and background incidences derived from the literature (one study) [22]. In addition, two studies [23,24] used influenza vaccines as reference comparator (Supplementary Table S3). Three studies used the same database (VigiBase) [23,24,26], but focused on distinct outcomes (Supplementary Table S6), while a restriction window was applied in only one study [22].

When mRNA vaccines were compared with all other therapies as the reference group, RORs exceeding 2 were observed for polymyalgia rheumatica, giant cell arteritis, IgA vasculitis with renal involvement, systemic sclerosis, and Sjögren’s syndrome, whereas more modest elevations (ROR < 2) were observed for microscopic polyangiitis, rheumatoid arthritis, systemic lupus erythematosus, Behçet’s syndrome, and ankylosing spondylitis (Table 2, Supplementary Table S7). In contrast, Jarrot et al. [22] reported lower crude incidence rates of giant cell arteritis and polymyalgia rheumatica among individuals vaccinated with BNT162b2 or mRNA-1273 compared with background literature data estimates across all doses, without reporting adjusted relative effect estimates; incidence rates varied by dose but did not indicate an increased signal following vaccination (Supplementary Table S8). When compared with all other vaccines, the RORs for SLE and lupus nephritis were lower [27].

In age-stratified disproportionality analyses using all reported medications as reference, one study reported variation in signal strength by age, with relatively stronger signals observed in younger (<18 years) as well as older (≥65 years) individuals (Supplementary Table S9) [26]. When compared with influenza vaccines, most previous associations were neutral or indicated a lower reporting frequency, except for Behçet’s disease, which retained a significant signal (ROR 4.2, CI95%: 1.3–13.2) (Table 2).

Two studies conducted direct head-to-head comparisons between mRNA vaccine [21,24] and showed a modestly higher tendency of systemic vasculitis with BNT162b2 [24] (Supplementary Table S7).

### 3.3. Population-Based Comparative Studies

A total of seven studies, including one RCT safety analysis [34] and six observational studies [28,29,30,31,32,33], were included. These studies leveraged large population-based datasets from South Korea, Israel, China, Norway, and the United States between 2020 and 2024. The largest cohorts were derived from national health databases in South Korea [28,29,30] and Israel [32], collectively encompassing several million individuals exposed to mRNA COVID-19 vaccines. Four of the studies focused exclusively on mRNA vaccines (BNT162b2, mRNA-1273), while the others reported additional data on adenoviral vector or inactivated vaccines. Comparators were derived from historical pre-vaccination cohorts in two studies [28,29], a contemporaneous unvaccinated cohort in one study [32], or both historical and contemporaneous cohorts in three studies [30,31,33]. Follow-up risk windows varied across studies and included 28 days [31], 30 days [33], 3 months [30], 12 months [32], or no prespecified restriction [28]. The randomised controlled trial used a placebo comparator with a 28-day safety window [34] (Table 3, Supplementary Table S4).

When mRNA vaccines were analysed collectively, a modest significant association was observed for systemic lupus erythematosus compared with a historical cohort (aHR 1.16, 95% CI 1.02–1.32), driven primarily by BNT162b2 recipients (aHR 1.18, 95% CI 1.02–1.36) (Jung et al., 2024) [29]. In contrast, Ju et al. (2023) and Bøås et al. (2026) found no significant association between mRNA vaccination and SLE [28,33] (Supplementary Tables S10–S12).

Gender-stratified analyses of both overall mRNA vaccines and BNT162b2, across multiple dosing regimens, showed no gender-based differences in risk, including for systemic lupus erythematosus (Supplementary Table S13). Age-stratified analysis identified an increased risk only for polymyalgia rheumatica in older adults after the second BNT162b2 dose (aHR 2.12, 95% CI 1.30–3.47) [32] (Supplementary Table S14). Among individuals with prior SARS-CoV-2 infection, systemic lupus erythematosus incidence was higher compared with infection-naive individuals (aHR 1.23, 95% CI 1.05–1.44) [29] (Supplementary Table S15). When divided by homologous and heterologous mRNA vaccination schedules, a modestly increased risk of systemic lupus erythematosus was noted for homologous regimens (aHR 1.24, 95% CI 1.08–1.43) [29] (Supplementary Table S16). Jung et al. [29] identified modest elevations in systemic lupus erythematosus risk in selected stratified analyses, but further booster-dose analyses indicated a potential increase in risk only for rheumatoid arthritis (aHR 1.14, 95% CI 1.08–1.21) (Supplementary Table S17). In analyses extending beyond the 28-day post-vaccination risk window, a modest increase in systemic lupus erythematosus incidence was reported, as presented in Supplementary Table S18 [31].

**Figure 2:**
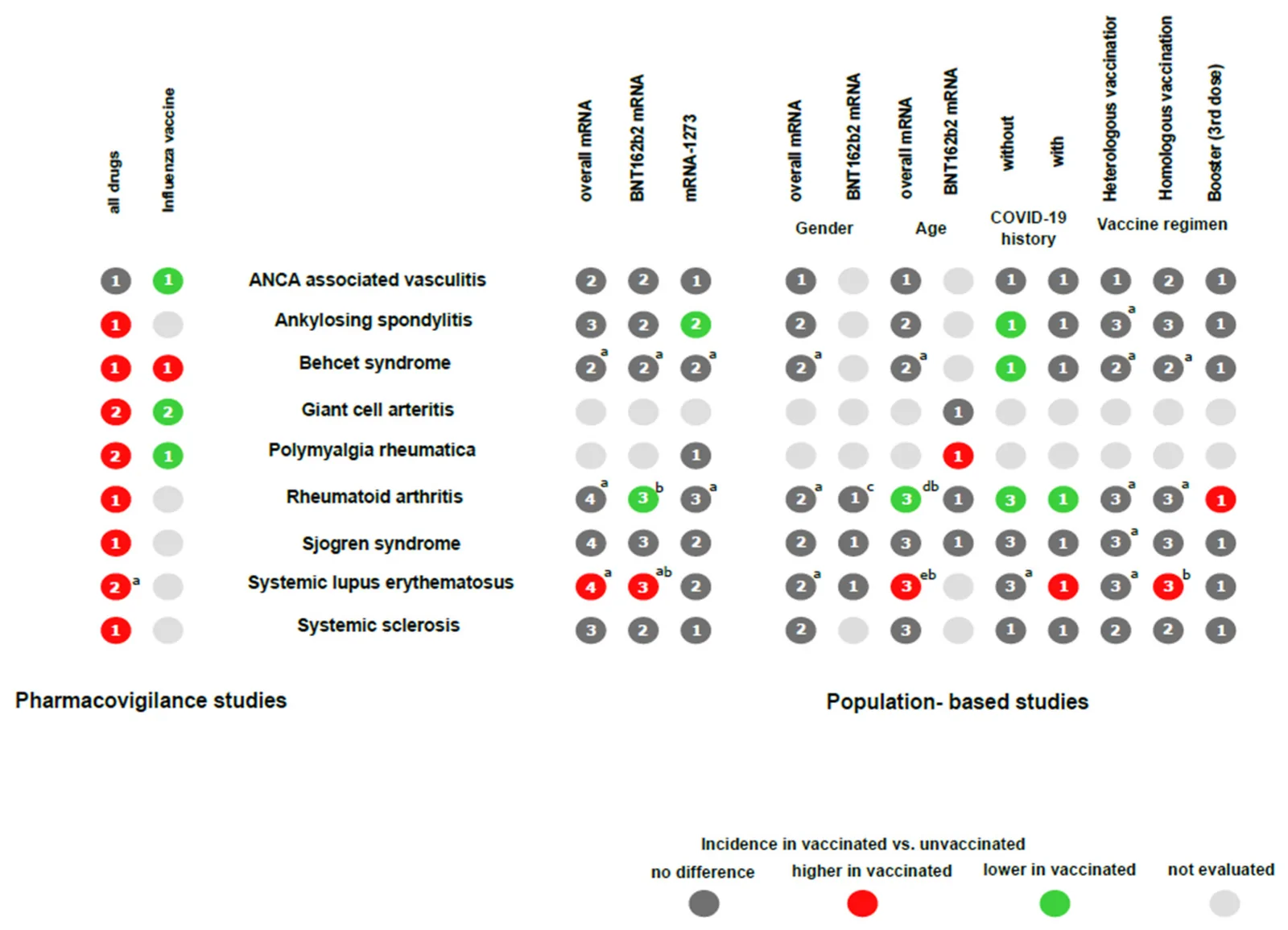
Summary of evidence on incident systemic autoimmune inflammatory rheumatic diseases following the mRNA COVID-19 vaccination. Disproportionality signals are shown by reference group (all other adverse event reports for any medicinal product (‘all other drugs’), all other vaccines or influenza vaccines), and population-based comparative studies by vaccine product and selected stratifiers. Circle colour indicates the direction of association (increased, null or decreased incidence/reporting), and pale grey indicates outcomes not evaluated. Numbers represent contributing analyses; superscripts indicate at least one study reporting lower incidence among vaccinated individuals (a) or no difference (b), lower incidence in females (c), and lower incidence in patients ≥ 40 years, with a neutral association in those <40 years (d). Findings were inconsistent between studies—Study 1: Lower incidence in patients < 40 years, and neutral association in those ≥40 years. Study 2: Higher incidence in patients ≥ 40 years, and neutral association in those <40 years (e). The reporting odds ratios quantify disproportionate reporting within spontaneous reporting systems and must not be interpreted as relative risks, incidence rate ratios, or measures of disease incidence.

Long-term evidence was scarce: only four studies from three countries (South Korea, Israel, and Norway) followed participants for up to approximately one year, and three of these reported at least one positive association—including systemic lupus erythematosus, post-booster rheumatoid arthritis, and polymyalgia rheumatica in older adults—whereas studies with primary risk windows of three months or less reported no increase. Figure 2 summarises the direction of findings across outcomes and study designs, highlighting that pharmacovigilance signals for selected conditions were comparator-dependent and were not consistently reproduced in population-based comparative analyses.

### 3.4. Risk of Bias

Overall, the pharmacovigilance studies performed well across most READUS-PV domains, with statistical analyses and 95% CIs consistently reported. However, key gaps included the lack of clear data selection flow diagrams and limited detail on data extraction or preprocessing. External validity and generalisability were only briefly addressed, constraining the interpretability of reported signals (Supplementary Table S19).

Cohort studies showed variable quality under ROBINS-I (Supplementary Figure S1). The main limitations were incomplete adjustment for confounders—particularly prior SARS-CoV-2 infection, previous vaccination, and underlying autoimmune disease—and heterogeneity in exposure and outcome definitions. Outcome misclassification and limited reporting on missing data contributed additional concerns.

The included RCT demonstrated generally low concerns in the randomisation process and adherence to assigned intervention. However, autoimmune outcomes were infrequent, not prespecified, and reported with limited detail, resulting in some concerns regarding outcome measurement and selective reporting (Supplementary Figure S2).

## 4. Discussion

In this systematic review, we used mRNA COVID-19 vaccination as the first population-scale exposure model for evaluating immune-mediated safety signals from an mRNA vaccine platform. Across international pharmacovigilance datasets and large population-based studies involving millions of vaccine recipients, we found no consistent evidence of a population-level increase in incident EULAR-defined systemic AIRDs after BNT162b2 or mRNA-1273 vaccination. The findings were heterogeneous and sensitive to methodological choices, particularly comparator selection and risk-window definition. Disproportionality analyses suggested increased reporting of polymyalgia rheumatica and giant cell arteritis, and less consistently selected vasculitides and connective tissue diseases, when all other drugs were the reference; however, most signals were null when influenza vaccines were used as comparators, with Behçet’s disease as a notable exception. As disproportionality metrics reflect reporting behaviour rather than incidence, these context-dependent signals are most plausibly explained by reporting bias, differential reporting, stimulated reporting, and comparator heterogeneity, rather than reflecting a causal inference.

Across cohort and trial data, the reported associations were largely inconsistent. However modest increases in systemic lupus erythematosus were reported only in selected analyses, primarily among individuals with history of prior COVID-19, and in the context of homologous schedules and longer follow-up. Discrepant findings regarding systemic lupus erythematosus between the results reported on the same underlying cohort by Ju et al. (2023) [28] and Jung et al. (2024) [29] likely reflect methodological differences rather than true biological divergence. Most notably, Jung et al.’s longer follow-up period and larger sample size may have better captured the delayed onset of systemic lupus erythematosus and provided sufficient statistical power to detect even a modest association. In contrast, the earlier analysis made by Ju et al. may have been too underpowered to evaluate rare outcomes. Extended follow-up through December 2022 may also better reflect normalised healthcare utilisation, thereby reducing the likelihood of case under-detection during earlier phases of the pandemic.

Overall, across all included studies, new-onset systemic AIRDs were consistently rare, with background incidence rates typically in the range of a few cases per million persons per year [35,36,37]. The interpretation of baseline incidence during the COVID-19 pandemic, however, warrants caution, as SARS-CoV-2 infection itself has been associated with immune-mediated disease onset [38], which may inflate post-vaccination incidence estimates in observational studies. Supporting this interpretation, a population-based study from the United Kingdom observed a temporal increase in giant cell arteritis diagnoses during the early pandemic, with peaks in SARS-CoV-2 infection preceding increases in giant cell arteritis occurrence by approximately 40–45 days [39], suggesting infection-related immune activation rather than vaccine-specific effects.

Several biological mechanisms have been proposed to explain rare autoimmune events temporally following vaccination, including molecular mimicry, bystander immune activation [40] and type I interferon signalling induced by lipid nanoparticle–mediated mRNA delivery [41]. In addition, host-related factors may also play a role, as genetic susceptibility appears to influence immune responses; for example, carriers of HLA-DRB1*04, a known risk allele for giant cell arteritis and polymyalgia rheumatica, may exhibit heightened inflammatory responses [42] that could predispose to disease onset independent of vaccination.

Notably, most large cohort studies assessed mRNA vaccines as a class; although some reported product-specific incidence estimates for BNT162b2 and mRNA-1273, the lack of direct head-to-head comparative effect estimates precluded formal comparisons between individual mRNA vaccines.

To our knowledge, this is the first review to explicitly separate pharmacovigilance signal detection evidence from denominator-based comparative risk evidence for EULAR-defined systemic AIRDs after mRNA vaccination. The extensive prespecified search strategy, prospective protocol registration, and the restriction to EULAR-defined systemic AIRDs enhance the robustness, clinical relevance, and generalisability of the findings in the context of expanding mRNA platform use beyond COVID-19 [43]. However, several limitations should be considered when interpreting the systematic review findings presented.

Pharmacovigilance systems are inherently constrained by underreporting, incomplete clinical information, and lack of standardised diagnostic validation [44]. Moreover, a proportion of reports are submitted by consumers rather than clinicians [45], which may further compromise diagnostic precision. For example, the pharmacovigilance-derived signal for polymyalgia rheumatica in younger individuals [26] is inconsistent with established disease epidemiology and likely reflects the diagnostic misclassification of non-specific inflammatory arthritis or coding artefacts rather than true early-onset disease. However, in the review by Irani et al. [46], reported PMR/GCA cases were most frequent after BNT162b2 vaccination, followed by ChAdOx1-S, mRNA-1273, and Ad26.COV2.S; fewer cases were described after zoster, influenza, hepatitis B, and tetanus vaccines. These descriptive findings support continued signal monitoring but cannot determine whether vaccination increases polymyalgia rheumatica incidence above its already high background incidence in older adults, and they remain particularly susceptible to temporal coincidence. Accordingly, signals identified through disproportionality analyses should be interpreted as hypothesis-generating rather than causal evidence [47].

Registry-based cohort studies provide more standardised case ascertainment, yet they remain susceptible to residual confounding. Adjustment for key confounders—including prior SARS-CoV-2 infection, previous vaccine regimens, dose numbers, and underlying autoimmune disease—was inconsistent, with only several cohort studies adequately controlling for these factors.

Follow-up periods varied substantially, ranging from short, predefined windows to extended or unrestricted surveillance, which may introduce time-related biases. Most events would be expected to appear within the first weeks after vaccination, consistent with the commonly used 21-day risk window [48]; however, only three studies clearly defined the disease onset intervals [24,25,31], limiting temporal interpretation. Narrow windows carry their own limitation, as autoimmune diseases are subject to delayed recognition and prolonged diagnostic pathways, so cases diagnosed later may be missed. Conversely, only a small number of studies followed participants for approximately 12 months and their findings were not fully concordant [28,29,32,33]—most notably the discordant systemic lupus erythematosus estimates obtained from the same Korean NHIS cohort by Ju et al. and Jung et al. Observed post-vaccination events comprise both temporally coincident background disease and any potentially vaccine-associated events. Because background cases accumulate approximately in proportion to person-time, whereas a vaccine-triggered effect would be expected to cluster within a biologically plausible interval after exposure, longer undifferentiated windows may dilute short-term signals while increasing opportunities for residual confounding. Conversely, very short windows may miss diseases with delayed clinical recognition and prolonged diagnostic pathways. Small delayed or disease-specific associations cannot be excluded, as illustrated by the higher systemic lupus erythematosus estimates obtained with longer follow-up [31], but long observation windows remain particularly susceptible to residual confounding and to the dilution of a temporally concentrated effect.

Two recent self-controlled case series directly tested the two strongest pharmacovigilance signals in our review. A South Korean analysis found no increased risk of incident polymyalgia rheumatica after COVID-19 vaccination, with stable estimates across 14-, 42-, and 60-day risk windows [49]. Shetty et al., using two independent Australian datasets, found no increased risk of incident giant cell arteritis within 1–42 days of mRNA vaccination (relative incidences 0.78, 95% CI 0.58–1.08 and 0.78, 95% CI 0.65–0.94), with consistent findings across vaccine types, ages, sexes, and shorter risk windows [50]. Both designs use self-controlled rather than unvaccinated comparators and therefore fall outside our PICO, but their concordant null findings reinforce the analytical evidence above. In addition, several cases initially attributed to vaccination were subsequently determined to be unrelated [51], underscoring the potential for time-to-onset misclassification.

Pharmacovigilance datasets generally lack reliable exposure denominators [16] and, without an unvaccinated comparator, cannot distinguish coincidental temporal clustering from vaccine-related events. Even if the cohort studies partially mitigate this limitation, they remain vulnerable to misclassification of onset timing, diagnostic coding variability, and unmeasured confounding [52].

In addition, dose-specific evidence was sparse: few studies stratified risk by dose, and only one compared booster recipients with non-recipients [29]. Given the potential for cumulative immune effects, this lack of multi-dose and booster data remains a key evidence gap.

It is also notable that the geographical representation is uneven, with most cohort studies conducted in upper-middle- and high-income countries, particularly in Asia, which may limit generalisability to lower-resource settings. Although pharmacovigilance systems capture reports from a broader range of regions, substantial variability in reporting practices introduces additional uncertainty [53]. Generalisability is limited by geographic concentration and by cross-setting differences in coding practices, access to speciality care, and the completeness and timeliness of administrative data capture. Differences in rollout timing and pandemic-related shifts in healthcare utilisation may also have affected the probability and timing of AIRD diagnosis, complicating comparisons across countries and calendar periods.

Lastly, due to substantial heterogeneity in outcome measures, vaccine types, dosing regimens, and comparator groups, a meta-analysis could not be conducted.

Future studies should prioritise active comparators, harmonised AIRD case definitions, validated diagnostic algorithms, standardised risk windows, and adjustment for prior SARS-CoV-2 infection. Self-controlled designs may be particularly useful for rare outcomes because they reduce confounding by stable individual characteristics. Multinational linked-data collaborations will be needed to evaluate disease-specific risks with adequate precision, especially as mRNA platforms expand beyond COVID-19. The present review was restricted to incident disease and does not address flares or worsening of established AIRDs, but the frequency and determinants of post-vaccination flares constitute a distinct clinical question requiring separate systematic assessment [54].

## 5. Conclusions

Pharmacovigilance signals for incident systemic AIRDs after mRNA COVID-19 vaccination were strongly comparator-dependent and were generally not corroborated by analytical evidence. The available data do not support a consistent population-level increase in systemic AIRDs after mRNA vaccination, although small or subgroup-specific effects remain possible. Delayed-onset disease nonetheless remains poorly characterised, as only four of the included studies extended observation towards twelve months; more robust evidence on this association will require studies designed with a minimum of one year of follow-up. The central lesson for rheumatology is that autoimmune safety signals from spontaneous reporting systems should be treated as hypothesis-generating and tested against denominator-based, comparator-controlled evidence before informing clinical counselling or regulatory interpretation.

## Figures and Tables

**Table 1:** Pharmacovigilance (Disproportionality analyses).

Author, Year Funding	Database Period Analysed	Cases	Non-Cases	Number of Reports Involving Exposure	Reference Set Used for Disproportionality	Risk Window
Fraenza et al., 2025 [21] Funding: NR	Eudra Vigilance 1 January 2021 to 23 October 2023	Rheumatic and immune-mediated diseases adverse events	All other adverse events	Tozinameran (BNT162b): 24,590 Elasomeran (mRNA-1273): 7703 ChAd: 10,619 Ad26.Cov2.S: 1814	All other COVID vaccines included in the analysis	No risk window
Jarrot et al. 2024 ^1^ [22] Funding: No	France pharmacovigilance database 27 December 2020 to 30 August 2022	PMR, GCA	None	BNT162b2 mRNA Overall: 50,587,266 doses 1st: 17,554,205 doses 2nd: 17,851,086 doses 3rd: 15,181,975 doses mRNA-1273 Overall: 11,924,026 doses 1st: 2,731,426 doses 2nd: 3,354,939 doses 3rd: 5,837,661 doses	Literature data of unvaccinated population	1 month risk window
Mettler et al., 2021 [23] Funding: No	VigiBase until 30 June 2021	Giant cell arteritis and polymyalgia rheumatica	All other adverse events	mRNA and viral vector vaccines: 1,295,482	All drugs: 24,950,901 Influenza vaccine: 317,687	No risk window
Mettler et al., 2022 [24] Funding: No	VigiBase until 31 March 2022	Systemic vasculitis	All other events	mRNA vaccines: 2,499,457	Any drugs: 30,031,000 Influenza vaccine: NR	No risk window
Nakao et al., 2023 [25] Funding: No	Japanese Adverse Drug Event Report (JADER) April 2004 to May 2022	IgA nephropathy, lupus nephritis	All other adverse events	mRNA vaccine: 21,455	All other drugs: 697,885	No risk window
Oh et al., 2024 [26] Funding: Public/Government	VigiBase inception (1967) to 2023	Rheumatic disease	All other adverse events	mRNA vaccines: 4,009,826 Ad5 vectored vaccines: 1,266,581 inactive whole virus vaccine: 162,570 non-COVID vaccines: 2,447,710 *	All other medications: 123,368,731	No risk window
Xie et al., 2026 [27] Funding: None	VAERS 1990 to March 2025	Lupus/SLE adverse events	Other VAERS reports	mRNA vaccine: 986 lupus AEs (49.90%)	Other vaccine technology/platform categories	No risk window

Abbreviations: ROR = reporting odds ratio; IC = information component; mRNA vaccines = Tozinameran (BNT162b2, Pfizer–BioNTech), Elasomeran (mRNA-1273, Moderna); NR = non-reported. * Self-calculated values. ROR and IC are disproportionality measures used in pharmacovigilance signal detection; values > 1 (or positive IC) indicate higher-than-expected reporting. ^1^ This analysis differs methodologically from disproportionality-based PV studies. All drugs: the reference set was all other adverse event reports for any medicinal product (‘all other drugs’).

**Table 2:** Pharmacovigilance (disproportionality analyses).

Autoimmune Disease	mRNA vs.		Author, Year
	All Other Drugs	Influenza Vaccine	
ANCA associated vasculitis	0.9 (0.8–1.0)	0.4 (0.3–0.4)	Mettler et al., 2022 [24]
ANCA positive vasculitis	0.9 (0.7–1.1)	0.3 (0.2–0.5)	Mettler et al., 2022 [24]
Ankylosing spondylitis	1.44 (1.32–1.56)	NR	Oh et al., 2024 [26]
Behcet syndrome	1.7 (1.4–2.1)	4.2 (1.3–13.2)	Mettler et al., 2022 [24]
Cryoglobulinaemic vasculitis	1.2 (0.8–1.6)	0.6 (0.3–1.2)	Mettler et al., 2022 [24]
Eosinophilic granulomatosis with polyangiitis	0.5 (0.4–0.6)	0.4 (0.2–0.7)	Mettler et al., 2022 [24]
Giant cell arteritis	3.2 (2.5–4.1)	0.6 (0.4–0.8)	Mettler et al., 2021 [23]
	4.5 (4.0–5.0)	0.7 (0.6–0.9)	Mettler et al., 2022 [24]
Giant cell arteritis and Polymyalgia rheumatica	3.2 (1.4–7.3)	0.3 (0.1–0.5)	Mettler et al., 2021 [23]
Granulomatosis with polyangiitis	1.3 (1.0–1.7)	0.3 (0.2–0.4)	Mettler et al., 2022 [24]
IgA vasculitis (Henoch Schonlein purpura)	0.7 (0.6–0.8)	0.1 (0.1–0.1)	Mettler et al., 2022 [24]
IgA vasculitis (nephropathy)	6.49 (4.38–9.61)	NR	Nakao et al., 2023 [25]
Microscopic polyangiitis	2.6 (1.8–3.7)	0.5 (0.2–0.9)	Mettler et al., 2022 [24]
Polyarteritis nodosa	0.4 (0.2–0.5)	0.3 (0.1–0.5)	Mettler et al., 2022 [24]
Polymyalgia rheumatica	4.1 (3.6–4.7)	0.4 (0.4–0.5)	Mettler et al., 2021 [23]
	34.98 (32.63–37.49)	NR	Oh et al., 2024 [26]
Rheumatoid arthritis	1.99 (1.93–2.05)	NR	Oh et al., 2024 [26]
Sjogren’s syndrome	2.92 (2.65–3.23)	NR	Oh et al., 2024 [26]
Systemic lupus erythematosus	NR	NR	Fraenza et al., 2025 [21]
	1.79 (1.68–1.91)	NR	Oh et al., 2024 [26]
	0.62 (0.56–0.69)	NR	Xie et al., 2026 [27]
Systemic lupus erythematosus (nephritis)	0.59 (0.48–0.72)	NR	Nakao et al., 2023 [25]
	0.65 (0.41–1.03)	NR	Xie et al., 2026 [27]
Systemic sclerosis	3.55 (2.76–4.58)	NR	Oh et al., 2024 [26]
Takayasu arteritis	0.5 (0.3–1.0)	NR ^c^	Mettler et al., 2022 [24]

Disproportionality results are expressed as reporting odds ratios (RORs) with 95% confidence intervals. Overall mRNA refers to both BNT 162b2 (Pfizer) and mRNA-1273 (Moderna) vaccination. Disproportionality estimates depend on the reference set and are not directly comparable across columns. Cell colours indicate statistically significant disproportionality of reporting: ∎ Lower reporting (significant), ∎ Higher-than-expected reporting (significant), □ No disproportionality detected. ^c^ insufficient cases observed with the studied drugs. Abbreviations: ANCA = anti-neutrophil cytoplasmic antibodies; mRNA = messenger ribonucleic acid, NR = not reported.

**Table 3:** Population-based comparative studies.

Author, Year Study Type Funding	Vaccines Evaluated by Article	Vaccinated Year and Source Sample Size	Unvaccinated Group Year and Source Sample Size	Outcome Operational Outcome Definitions	Authors Conclusions	Time Window
El Sahly et al., 2021 [34] USA COVE trial; NCT04470427 Funding: Mixed (Public–Private: HHS/BARDA and Moderna Inc.)	mRNA-1273	Adults (≥18 years) across 99 U.S. sites-COVE trial dataset (randomised 27 July to 23 October 2020) N = 15,209 participants	Placebo (randomised 27 July to 23 October 2020) N = 15,206 participants	Safety outcomes—vaccine-related adverse events MedDRA	Autoimmune adverse events infrequent and not prespecified; no signal for systemic AIRDs versus placebo.	28 days restriction
Li et al., 2022 [31] China Retrospective cohort Funding: Public (Food and Health Bureau, HKSAR)	BNT162b2 mRNA CoronaVac	Hong Kong vaccination records of all residents ≥ 16 years (23 February 2021 to 30 June 2021) 3.9 million Hong Kong residents BNT162b2 1st dose: 579,998 participants 2nd dose: 388,881 participants	Historical (pre-vaccine) and concurrent: electronic medical record from the Hospital Authority of all residents ≥ 16 years. (1 January 2018 to 30 June 2021) 1st dose: 2,816,133 participants 2nd dose: 1,892,783 participants	Hospital admission related to 16 pre-specified autoimmune diseases ICD-9 CM	No increased risk of the 16 pre-specified autoimmune diseases within 28 days of dose 1 or 2.	28 days after 1st or 2nd dose
Ju et al., 2023 [28] Republic of Korea (South Korea) Retrospective cohort Funding: Public + Institutional (NRF Korea, Korea Medical Institute)	BNT162b2 mRNA mRNA-1273	Korea Disease Control and Prevention Agency-COVID-19-National Health Insurance Service (NHIS) cohort. (index date to 31 December 2021) at least 1 dose of mRNA vaccine N = 3,838,120 participants	Historical (pre-vaccine): Korea Disease Control and Prevention Agency-COVID-19-National Health Insurance Service (NHIS) cohort. (shifted back by 1 year-index date to 21 December 2020) matched by age and gender. N = 3,834,804 participants	Autoimmune adverse events (connective tissue disorders) ICD-10	No significant association between mRNA vaccination and autoimmune skin and connective-tissue/autoimmune disorders, including SLE (aHR < 1).	No restriction (end of follow-up)
Jung et al., 2024 [29] Republic of Korea (South Korea) Retrospective cohort Funding: No	BNT162b2 mRNA mRNA-1273	Korea Disease Control and Prevention Agency-COVID-19-National Health Insurance Service (NHIS) cohort. (index date to 31 December 2022) at least 1 dose of mRNA vaccine N = 4,445,333 participants	Historical (pre-vaccine): Korea Disease Control and Prevention Agency-COVID-19-National Health Insurance Service (NHIS) cohort. (follow-up period up to 31 December 2020) N = 4,444,932 participants	Autoimmune connective tissue diseases ICD-10	Most systemic AIRDs were not associated with mRNA vaccination; Modest SLE increase (aHR 1.16, 1.02–1.32), driven by BNT162b2; RA increase after booster (aHR 1.14, 1.08–1.21); higher SLE with prior SARS-CoV-2 and homologous schedules.	Follow-up of at least 365 days
Kim et al., 2024 [30] Republic of Korea (South Korea-Seoul) Retrospective cohort Funding: No	BNT162b2 mRNA mRNA-1273 AZD1222 JNJ-78436735	National Health Insurance service database adult participants ≥ 20 years (1 January 2020 to 31 December 2021) N = 2,086,709 participants (all vaccines) BNT162b2 mRNA 1st dose: 1,172,254 participants 2nd dose: 1,308,228 participants mRNA-1273 1st dose: 30,277 participants 2nd dose: 30,305 participants	Historical (pre-vaccine) and concurrent: National Health Insurance service database adult participants ≥ 20 years (1 January 2020 to 31 December 2021) N = 343,311 participants	Autoimmune adverse events ICD-10	Autoimmune adverse events assessed at 1 week–3 months; no consistent increase across regimens.	1 week, 2 weeks, 1 month and 3 months restriction after vaccination
Shani M, 2024 [32] Israel Retrospective cohort Funding: No	BNT162b2 mRNA	CHS members aged 12 years or older (December 2020 to December 2021) two doses of BNT162b2 mRNA N = 2,455,207 participants	Concurrent: CHS members aged 12 years or older unvaccinated (December 2020 to December 2021) N = 594,879	24 immune-mediated diagnoses ICD-9	Age-stratified increase in PMR after the second BNT162b2 dose in older adults (aHR 2.12, 1.30–3.47); psoriasis, colitis, and vitiligo also increased.	4 to 12 months after vaccination
Bøås et al., 2026 [33] Norway Retrospective nationwide cohort Funding: None	overall mRNA vaccination	BeredtC19 emergency preparedness register for COVID-19; all Norwegian residents aged 18–64 residing in Norway since 1 January 2017. N = 3,115,221 participants. 1st dose: 89,833 participants 2nd dose: 863,667 participants 3rd dose: 2,161,712 participants	Historical (pre-vaccine) and concurrent: time-varying unexposed person-time within the same nationwide cohort. After 180 days post exposure, participants returned to unexposed reference until the next mRNA vaccination or SARS-CoV-2 infection. N = 334,859 participants	New-onset immune-mediated diseases. ICD-10	mRNA vaccination was associated with IBD, coeliac disease, inflammatory arthritis, erythema nodosum, arthralgia, agranulocytosis, and acute disseminated encephalomyelitis, alongside a reduced risk of dermatopolymyositis	Primary risk windows: 0–30 days and 30–180 days. Secondary restriction window: 0–30 days and 30–365 days.

Abbreviations: ZD1222, ChAdOx1 nCoV-19 adenoviral vector vaccine (Oxford–AstraZeneca COVID-19 vaccine); BARDA, Biomedical Advanced Research and Development Authority; BNT162b2, Pfizer–BioNTech mRNA COVID-19 vaccine; CDC: Centers for Disease Control; CHS, Clalit Health Services; COVE trial, Coronavirus Efficacy (COVE) phase 3 trial of mRNA-1273; COVID-19, Coronavirus Disease 2019; GCA: giant cell arteritis; HHS, U.S. Department of Health and Human Services; HKSAR, Hong Kong Special Administrative Region; ICD: International Classification of Diseases; JNJ-78436735, Ad26.COV2.S adenoviral vector vaccine (Johnson & Johnson/Janssen); PMR, polymyalgia rheumatica; mRNA, messenger ribonucleic acid; mRNA-1273, Moderna mRNA COVID-19 vaccine; N, number of participants; NCT, National Clinical Trial identifier (ClinicalTrials.gov); NHIS, National Health Insurance Service (Republic of Korea); NRF, National Research Foundation of Korea; VAERS, Vaccine Adverse Event Reporting System; USA, United States of America.

## Data Availability

No new primary data were generated in this study. All data analyzed in this systematic review were obtained from previously published studies and are available in the original articles cited in the manuscript. The data extracted and analyzed for this review are included in the article and/or its Supplementary Materials.

## Supplementary Materials

The following supporting information can be downloaded at: https://www.mdpi.com/article/10.3390/vaccines14080706/s1, Table S1. Search strings for each database; Table S2. PICO Framework for Study Selection (Inclusion and Exclusion Criteria); Table S3. Pharmacovigilance (Disproportionality analyses); Table S4. Population-based comparative studies; Table S5. Confounding variables considered across the included studies; Table S6. Evidence map of assessed outcomes across included studies; Table S7. Potential signals within disproportionality analyses; Table S8. Dose stratified analyses; Table S9. Age stratified analyses; Table S10. Overall mRNA vaccines; Table S11. BNT162b2 mRNA (Pfizer) vaccination; Table S12. mRNA-1273 (Moderna) vaccination; Table S13. Gender stratified analyses; Table S14. Age stratified analyses; Table S15. COVID-19 diagnosis; Table S16. Incidence risk following homologous and heterologous mRNA vaccination relative to unvaccinated; Table S17. Booster vaccination (3rd dose); Table S18. Follow-up restriction window; Table S19. Disproportionality analyses (READUS-PV reporting framework); Table S20. Characteristics of ongoing clinical trials evaluating COVID-19 mRNA vaccines; Figure S1. Cohort studies (ROBINS I version 2); Figure S2. RCTs (RoB 2).

## Abbreviations

The following abbreviations are used in this manuscript:

COVID-19	Coronavirus Disease 2019
mRNA	Messenger ribonucleic acid
AIRDs	Autoimmune inflammatory rheumatic diseases
EULAR	European Alliance of Associations for Rheumatology
SARS-CoV-2	Severe Acute Respiratory Syndrome Coronavirus 2
PV	Pharmacovigilance
VAERS	Vaccine Adverse Event Reporting System
PRISMA	Preferred Reporting Items for Systematic Reviews and Meta-Analyses
ICTPR	International Clinical Trials Registry Platform
STROBE	Strengthening the Reporting of Observational Studies in Epidemiology
READUS-PV	Reporting of Evidence-based Disproportionality Analyses for Pharmacovigilance
ROBINS-I	Risk Of Bias In Non-randomized Studies-of Interventions
RoB2	Risk of Bias 2
RCT	Randomised controlled trials
ROR	Reporting odds ratio
PRR	Proportional reporting ratio
IC	Information component
SLE	Systemic lupus erythematosus
