# Supplementary material for: Comparator-Dependent Safety Signals for Incident Systemic Autoimmune Rheumatic Diseases After mRNA COVID-19 Vaccination: A Systematic Review
Source: Vaccines (Basel). 2026 Aug 17;14(8):706. doi: 10.3390/vaccines14080706 (PMC13517954; doi:10.3390/vaccines14080706)
Supplement: Supplementary file 1 [file vaccines-14-00706-s001.zip › vaccines-4443099-supplementary.pdf]

Comparator-Dependent Safety Signals for Incident Systemic Autoimmune Rheumatic Diseases After mRNA COVID-19 Vaccination: A Systematic Review

Supplementary material

Table of contents

Section S1. Search strategy

Section S2. List of pathologies considered for evaluation

Section S3. PICO Framework for Study Selection (Inclusion and Exclusion Criteria)

Section S4. Characteristics of included studies
Table S3. Pharmacovigilance (Disproportionality analyses)
Table S4. Population-based comparative studies
Table S5. Confounding variables considered across the included studies
Table S6. Evidence map of assessed outcomes across included studies

Section S5. Pharmacovigilance (Disproportionality analyses): Reporting of new-onset autoimmune inflammatory rheumatic diseases
Table S7. Potential signals within disproportionality analyses
Table S8. Dose stratified analyses
Table S9. Age stratified analyses

Section S6. Population-based comparative studies: Incidence of new-onset autoimmune inflammatory rheumatic diseases
Table S10. Overall mRNA vaccines
Table S11. BNT162b2 mRNA (Pfizer) vaccination
Table S12. mRNA-1273 (Moderna) vaccination
Table S13. Gender stratified analyses
Table S14. Age stratified analyses
Table S15. COVID-19 diagnosis
Table S16. Incidence risk following homologous and heterologous mRNA vaccination relative to unvaccinated
Table S17. Booster vaccination (3rd dose)
Table S18. Follow-up restriction window

Section S7. Risk of bias for the included studies
Table S19. Disproportionality analyses (READUS-PV reporting framework)
Figure S1. Cohort studies (ROBINS I version 2)
Figure S2. RCTs (RoB 2)

Section S8. Ongoing trials

Section S9. List of included articles

Section S10. List of excluded articles as full text screening with reasons

Interpretation note:
The supplementary tables present extracted data from included studies for transparency and completeness. Pharmacovigilance disproportionality measures (e.g., reporting odds ratios, information components) reflect differential reporting patterns and are intended for hypothesis generation only; they do not estimate incidence, relative risk, or causality. Incidence measures reported across studies use heterogeneous denominators (e.g., per vaccinated persons, per administered doses, person-years) and are not directly comparable. Supplementary tables should not be interpreted in isolation or used to draw causal inferences. Interpretation of findings is provided exclusively in the main manuscript using a predefined framework that distinguishes signal detection from risk estimation.

Color legend:
Shading is used solely to indicate data availability or the presence of a reported estimate. Colors do not represent direction, magnitude, or clinical relevance of association and should not be interpreted as indicating increased or decreased risk.

Section S1. Search strategy

Table S1. Search strings for each database

**MEDLINE (PubMed)**<br>Initial search (3 April 2024): 2334 results<br>Last search update (25 April 2026): 2955 results			
Search line	Concept	Records (n)	Query
#1	mRNA vaccines	21,956	"rna, messenger"[MeSH Terms] OR ("rna"[All Fields] AND "messenger"[All Fields]) OR "messenger rna"[All Fields] OR "mrna"[All Fields]) AND "vaccine*"[All Fields]
#2	Adverse events	4,431,394	((("adverse"[All Fields] OR "adversely"[All Fields] OR "adverses"[All Fields]) AND "reaction*"[All Fields]) OR (("adverse"[All Fields] OR "adversely"[All Fields] OR "adverses"[All Fields]) AND "event*"[All Fields]) OR "complication*"[All Fields])
#3	Immunization/vaccination	1,886,524	("immune"[All Fields] OR "immuned"[All Fields] OR "immunes"[All Fields] OR "immunisation"[All Fields] OR "vaccination"[MeSH Terms] OR "vaccination"[All Fields] OR "immunization"[All Fields] OR "immunization"[MeSH Terms] OR "immunisations"[All Fields] OR "immunizations"[All Fields] OR "immunise"[All Fields] OR "immunised"[All Fields] OR "immuniser"[All Fields] OR "immunisers"[All Fields] OR "immunising"[All Fields] OR "immunities"[All Fields] OR "immunity"[MeSH Terms] OR "immunity"[All Fields] OR "immunization s"[All Fields] OR "immunize"[All Fields] OR "immunized"[All Fields] OR "immunizer"[All Fields] OR "immunizers"[All Fields] OR "immunizes"[All Fields] OR "immunizing"[All Fields])
#4	Combined	2955	# 1 AND #2 AND #3

A supplementary MEDLINE search was conducted on September 8th 2025 using expanded terms (omicron or "BA.1" or "BA.4" or "BA.5" or "BA.4-5" or "XBB.1.5" or "XBB.1.16" or "KP.2" or "JN.1" or "JN.1) to identify studies of Omicron-adapted mRNA vaccines, which frequently omit "mRNA" from indexing. The search retrieved no additional eligible studies.

**Web Of Science**<br>Initial search (3 April 2024): 2204 results<br>Last search update (26 April 2026): 2762 results			
Concept	Search line	Records (n)	Query
mRNA vaccines	#1	20,173	(ALL=(mRNA)) AND ALL=(vaccine*)
Adverse events	#2	6,186,885	((((ALL=(adverse)) AND ALL=(immune)) AND ALL=(complication*)) OR ALL=(event*)) OR ALL=(reaction*)
Combined	#3	3502	#1 AND #2
WOS category	#4	3355	Results for #5 AND #4 and Veterinary Sciences or Chemistry Applied or Mathematical Computational Biology or Plant Sciences or Materials Science Biomaterials or Food Science Technology or Zoology or Marine Freshwater Biology or Materials Science Multidisciplinary or Environmental Sciences or Agriculture Dairy Animal Science or Economics or Physics Condensed Matter or Social Issues or Social Sciences Biomedical or Agriculture Multidisciplinary or Business or Management or Polymer Science or Oceanography (Exclude – Web of Science Categories)
Citation Meso	#5	2762	Results for #5 AND #4 and Veterinary Sciences or Chemistry Applied or Mathematical Computational Biology or Plant Sciences or Materials Science Biomaterials or Food Science Technology or Zoology or Marine Freshwater Biology or Materials Science Multidisciplinary or Environmental Sciences or Agriculture Dairy Animal Science or Economics or Physics Condensed Matter or Social Issues or Social Sciences Biomedical or Agriculture Multidisciplinary or Business or Management or Polymer Science or Oceanography (Exclude – Web of Science Categories) and 6.146 Anthropology or 3.91 Contamination & Phytoremediation or 1.100 Substance Abuse or 1.136 Autism & Development Disorders or 1.258 Zoonotic Diseases or 6.27 Political Science or 1.184 Physiology & Metals or 3.4 Crop Science or 2.76 2d Materials or 3.40 Forestry or 3.87 Paper & Wood Materials Science or 4.13 Telecommunications or 5.20 Astronomy & Astrophysics or 6.153 Climate Change or 8.124 Environmental Sciences (Exclude – Citation Topics Meso) and Exclude Reviews or Retracted articles or Book chapters

**EMBASE (Elsevier)**<br>Initial search (3 April 2024): 1616 results<br>Last search update (25 April 2026): 2244 results			
Concept	Search line	Records (n)	Query
mRNA vaccines	#1	31,239	('mrna'/exp OR mrna) AND vaccine*
Adverse events	#2	176,862	adverse AND ('immune'/exp OR immune)
Immunization/vaccination	#3	10,669,467	(complication* OR event* OR reaction*)
Combined	#4	2244	# 1 AND #2 AND #3

**Scopus**<br>Initial search (3 April 2024): 3707 results<br>Last search update (25 April 2026): 5008 results			
Concept	Search line	Records (n)	Query
Search syntax	#1	17,299	(ALL (complication) OR ALL (event*) OR ALL (reaction*)) AND ALL (mrna) AND ALL<br>(vaccine*) AND ALL (adverse) AND ALL (immune)
Filter Subject area	#2	12,339	Limit to Medicine
Filter Document type	#3	6762	Exclude Review articles or Book chapters or Books or Retracted publications
Filter Keywords	#4	5008	Exclude Animals or Animal or Nonhuman or Animal Experiment or Human tissue or Mouse

**Cochrane Library**<br>Search date: 25 April 2026: 24 results			
Search line	Concept	Records (n)	Query
#1	mRNA vaccines	6,211	"mRNA" in all text
#2	Pathologies considered	60,088	"Rheumatoid Arthritis OR Spondyloarthritis OR Ankylosing Spondylitis OR Reactive Arthritis OR Psoriatic Arthritis OR Connective Tissue Diseases OR Antiphospholipid Syndrome OR Systemic Lupus Erythematosus OR Sjogren's Syndrome OR Systemic Sclerosis OR Dermatomyositis OR Polymyositis OR Still's Disease OR Anti-Neutrophil Cytoplasmic Antibody OR Polyarteritis Nodosa OR Takayasu Arteritis OR Giant Cell Arteritis OR Polymyalgia Rheumatica OR Behcet's Syndrome OR IgA Vasculitis OR Cryoglobulinemia OR Cogan's Syndrome" in All Text
#3	Combined	300	#1 AND #2
#4	Limit	24	#1 AND #2 limit to Trials, 2019 onwards

**medRxiv**<br>Search date: 25 April 2026: 278 results		
Search syntax	4560	"("COVID-19" OR "SARS-CoV-2") AND ("mRNA vaccine" OR "Pfizer" OR "BNT162b2" OR "Moderna" OR "mRNA-1273" OR "COVID-19 vaccination"
Filter	4560	and abstract or title "onset" (match all words) –
Filter Fields	278	in Rheumatology / allergy and immunology
**ClinicalTrials.gov**<br>Search date: 25 April 2026: 86 results		
Search syntax	1124	(COVID-19 OR SARS-CoV-2) AND (mRNA vaccine OR Pfizer OR BNT162b2 OR Moderna OR mRNA-1273 OR COVID-19 vaccination)
Limit to	86	Phase IV
**WHO ICTRP**<br>Search date: 25 April 2026: 108 results		
Search syntax	17536	(COVID-19 OR SARS-CoV-2) in the condition AND (mRNA vaccine OR Pfizer OR BNT162b2 OR Moderna OR mRNA-1273) in the intervention
Filter	108	limit to Phase IV

Section S2. List of pathologies considered for evaluation

In this systematic review, we define systemic autoimmune rheumatic diseases (AIRDs) as chronic, immune-mediated disorders characterized by autoantibody production or other evidence of autoimmunity, inflammation involving multiple organ systems (including, but not limited to, joints, skin, kidneys, vasculature, muscle), and established by disease-specific EULAR/ACR classification criteria (or validated diagnostic definitions).

- Rheumatoid Arthritis
- Spondyloarthritis (Ankylosing Spondylitis, Reactive Arthritis, Psoriatic Arthritis)
- Mixed Connective Tissue Disease
- Antiphospholipid Syndrome
- Systemic Lupus Erythematosus
- Sjogren's Syndrome
- Systemic Sclerosis/ Scleroderma
- Dermatomyositis/Polymyositis
- Still's Disease
- Anti-Neutrophil Cytoplasmic Antibody (ANCA)-Associated Vasculitis
- Polyarteritis Nodosa
- Large vessel vasculitis (Takayasu Arteritis, Giant Cell Arteritis, Polymyalgia Rheumatica)
- Behcet's Syndrome
- IgA Vasculitis (Henoch-Schönlein purpura)
- Cryoglobulinemic vasculitis
- Cogan's Syndrome

Section S3. Table S2. PICO Framework for Study Selection (Inclusion and Exclusion Criteria)

	Include	Exclude
**Population**	- General adult population (≥18 years)<br>- Regardless of prior COVID-19 history (both with and without prior infection)<br>-For cohort studies: minimum sample size ≥500 participants.<br>- Mixed populations included if ≥80% adults; otherwise excluded	- Studies focusing only on individuals with:<br>– autoimmune diseases (not limited to rheumatologic or systemic)<br>–immunosuppression regardless of indication<br>- Specific recruitment limitation (e.g., only ICU or only pregnant women)
**Intervention**	-mRNA COVID-19 vaccines (tozinameran [BNT162b2, Pfizer–BioNTech], elasomeran [mRNA-1273, Moderna]) — all doses included (primary, booster)<br>- Subgroup analysis for studies with mix-and-match vaccine boosters	- Any non-mRNA vaccine (e.g., viral vector, protein subunit, inactivated)<br>- Vaccine type not specified<br>- Without stratified results for mRNA vaccines (i.e., data combined with other vaccine types)<br>- Concomitant administration with other vaccine types
**Comparator**	- No intervention<br>- Placebo<br>- Historical background rates (including literature data)<br>- Head-to-head comparisons of BNT162b2 and mRNA-1273[1]	-Non mRNA vaccines
**Outcomes**	-New-onset systemic autoimmune rheumatic diseases , as defined in Appendix Section 2.	- Exacerbation or flare of pre-existing autoimmune diseases.<br>- Non-systemic or non-rheumatic autoimmune diseases (including e.g., overall autoimmune diseases without further data, single organ vasculitis, isolated musculoskeletal symptoms without a confirmed autoimmune disease diagnosis)<br>- Other unrelated outcomes
**Timing**	No minimum follow-up required	None
**Context**	Studies from all countries (classified by World Bank income level) [1]	None
**Study designs**	- Pharmacovigilance database studies<br>- Surveillance data analyses<br>- Randomized controlled trials (RCTs)- post-marketing or Phase III/IV with safety outcomes<br>- Prospective/retrospective cohort studies<br>- Case-control studies<br>- Cross-sectional studies.	- Case series, case reports<br>- Reviews, editorials, opinion pieces<br>- Experimental animal or in vitro studies
**Document types**	- Peer-reviewed, full-text articles.<br>- Preprints	- Abstracts, posters, letters, books, theses, chapters, or editorial materials
**Language**	No restrictions	None

Abbreviations: COVID-19: Coronavirus disease of 2019

[1] Head-to-head comparisons of BNT162b2 and mRNA-1273 were included only when effect estimates were provided in the primary studies.

[2]https://datahelpdesk.worldbank.org/knowledgebase/articles/906519-world-bank-country-and-lending-grou

Section S4. Characteristics of included studies

Table S3. Pharmacovigilance (Disproportionality analyses)

Author, year<br>Funding	Database<br>Period analysed	Cases	Non-cases	Number of reports involving exposure	Reference set used for disproportionality	Disproportion Measure**	Signal Definition<br>Risk Window	Confounders Considered	Subgroup Analysis
Fraenza et al., 2025 (1)<br><br>Funding:<br>NR	Eudra Vigilance<br><br>1st January 2021 to 23rdOctober 2023	Rheumatic and immune-mediated diseases adverse events	All other adverse events	Tozinameran (BNT162b): 24,590<br>Elasomeran (mRNA-1273): 7703<br>ChAd: 10,619<br>Ad26.Cov2.S: 1814	All other COVID vaccines included in the analysis	ROR	ROR ≥ 2 with lower boundary 95% of ROR > 1 or Chi-squared test of at least four (threshold value at one degree of freedom with $p < 0.05$ in a 2 × 2 table)<br><br>No risk window	Underlining rheumatic diseases	Yes (but not applicable for our systematic review)
Jarrot et al. 2024[1] (2)<br><br>Funding: No	France pharmacovigilance database<br><br>December 27th, 2020 to August 30th, 2022	PMR, GCA	None	**•BNT162b2 mRNA**<br>Overall: 50,587,266 doses<br>1st: 17,554,205 doses<br>2nd: 17,851,086 doses<br>3rd: 15,181,975 doses<br>**•mRNA-1273**<br>Overall: 11,924,026 doses<br>1st: 2,731,426 doses<br>2nd: 3,354,939 doses<br>3rd: 5,837,661 doses	Literature data of unvaccinated population	Reporting rate (Incidence rate)	Reporting rate (Incidence rate) reported to 10^6 administered doses<br><br>**1 month** risk window	None	Yes:<br>vaccine type, dose
Mettler et al., 2021 (3)<br><br>Funding:<br>No	VigiBase<br><br>until 30 June 2021	Giant cell arteritis and polymialgia rheumatica	All other adverse events	mRNA and viral vector vaccines: 1,295,482	All drugs:<br>24,950,901<br><br>Influenza vaccine:<br>317,687	ROR	NR<br><br>No risk window	Age "similar findings were retrieved after adjusting for age"	Yes:<br>vaccine type (mRNA, viral vector)
Mettler et al., 2022 (4)<br><br>Funding:<br>No	VigiBase<br><br>until 31 March 2022	Systemic vasculitis	All other events	mRNA vaccines:<br>2,499,457	Any drugs:<br>30,031,000<br><br>Influenza vaccine:<br>NR	ROR	Lower boundary 95% for ROR > 1 and<br>Number of cases > 3<br><br>No risk window	None	Yes:<br>vaccine type (tozinameran/BNT162b vs elasomeran/mRNA-1273)
Nakao et al., 2023 (5)<br><br>Funding:<br>No	Japanese Adverse Drug Event Report (JADER)<br><br>April 2004 to May 2022	IgA nephropathy, lupus nephritis	All other adverse events	mRNA vaccine:<br>21,455	All other drugs:<br>697,885	ROR	Lower boundary 95% of ROR>1 or positive value for IC_{025}<br><br>No risk window	Age, gender<br>.	Yes:<br>age, gender
Oh et al., 2024 (6)<br><br>Funding:	VigiBase<br><br>inception (1967) to	Rheumatic disease	All other adverse events	mRNA vaccines: 4,009,826<br>Ad5 vectored vaccines: 1,266,581<br>inactive whole virus vaccine:	All other medications:<br>123,368,731	ROR	Positive value for IC_{025} and lower boundary 95% for ROR ≥ 1	Age, gender	Yes:<br>vaccine type (mRNA, others)

Public/Government	2023			162,570<br>non-COVID vaccines: 2,447,710*			No risk window		age groups
Xie et al., 2026 (7)<br><br>Funding: None	VAERS<br><br>1990 to March 2025	Lupus/SLE adverse events	Other VAERS reports/adverse events not matching lupus/SLE	mRNA vaccine: 986 lupus AEs (49.90%)	Other vaccine technology/platform categories	ROR	ROR lower 95% CI >1; PRR ≥2 with chi-square ≥4 and ≥3 cases; IC025 >0<br><br>No risk window	None	Yes:<br>vaccine type (mRNA, others)

Abbreviations: ROR = reporting odds ratio; IC = information component; mRNA vaccines = Tozinameran (BNT162b2, Pfizer–BioNTech), Elasomeran (mRNA-1273, Moderna).
*Self-calculated values.
**ROR and IC are disproportionality measures used in pharmacovigilance signal detection; values >1 (or positive IC) indicate higher-than-expected reporting.
[1]This analysis differs methodologically from disproportionality-based PV studies.

Table S4. Population-based comparative studies

Author, year<br>Study type<br>Funding	Vaccines evaluated by article	Vaccinated<br>Year and source<br>Sample size	Unvaccinated group<br>Year and source<br>Sample size	Outcome	Measure of occurence<br>Time window	Secondary/<br>Subgroup analysis
Bøås et al., 2026 (8)<br>Norway<br>Retrospective nationwide cohort<br><br>Funding: None	mRNA vaccination (BNT162b2 mRNA, mRNA-1273)	BeredtC19 emergency preparedness register for COVID-19; all Norwegian residents aged 18-64 residing in Norway since January 1st , 2017.<br><br>N = 3,115,221 participants.<br>1st dose: 89,833 participants<br>2nd dose: 863,667 participants<br>3rd dose: 2,161,712 participants	**Historical (pre-vaccine) and concurrent:** time-varying unexposed person-time within the same nationwide cohort. After 180 days post exposure, participants returned to unexposed reference until the next mRNA vaccination or SARS-CoV-2 infection.<br><br>N = 334,859 participants	New-onset immune-mediated diseases.	Cox regression adjusted HRs.<br><br>Primary risk windows: 0-30 days and 30-180 days.<br>Secondary restriction window: 0-30 days and 30-365 days.	Yes:<br>age-stratified analyses 18-39 and 40-65 years; SARS-CoV-2 infection; follow-up restriction window.
El Sahly et al., 2021 (9)<br>USA<br>COVE trial;<br>NCT04470427<br>Funding:Mixed (Public–Private: HHS/BARDA and Moderna Inc.)	•mRNA-1273	Adults (≥18 years) across 99 U.S. sites- COVE trial dataset<br>(randomized July 27th to October 23rd, 2020)<br><br>N= 15,209 participants	**Placebo**<br>(randomized July 27th to October 23rd, 2020)<br><br>N= 15,206 participants	Safety outcomes – vaccine-related adverse events	Cumulative incidence rates reported/10^5 persons<br><br>**28 days restriction**	No
Ju et al., 2023 (10)<br>Republic of Korea (South Korea)<br>Retrospective cohort<br><br>Funding: Public + Institutional (NRF Korea, Korea Medical Institute)	•BNT162b2 mRNA<br>•mRNA-1273	Korea Disease Control and Prevention Agency-COVID-19-National Health Insurance Service (NHIS) cohort. (index date to December 31st, 2021)<br><br>at least 1 dose of mRNA vaccine<br><br>N = 3,838,120 participants	**Historical (pre-vaccine):** Korea Disease Control and Prevention Agency-COVID-19-National Health Insurance Service (NHIS) cohort. (shifted back by 1 year- index date to December 21st, 2020)<br>matched by age and gender.<br><br>N = 3,834,804 participants	Autoimmune adverse events (connective tissue disorders)	Cumulative incidence rates reported /10^4 person-years.<br>Adjusted Hazard ratios<br><br>**No restriction** (end of follow-up)<br>Median follow-up (days):<br>vaccinated: 100.7 (90.3)<br>control: 100.7 (88.5)	Yes:<br>secondary analysis by age, sex, type of mRNA vaccine and history of prior non-mRNA vaccination
Jung et al., 2024 (11)<br>Republic of Korea (South Korea)<br>Retrospective cohort<br><br>Funding: None	•BNT162b2 mRNA<br>•mRNA-1273	Korea Disease Control and Prevention Agency-COVID-19-National Health Insurance Service (NHIS) cohort. (index date to December 31st, 2022)<br><br>at least 1 dose of mRNA vaccine<br>N = 4,445,333 participants	**Historical (pre-vaccine):** Korea Disease Control and Prevention Agency-COVID-19-National Health Insurance Service (NHIS) cohort. (follow-up period up to December 31st, 2020)<br><br>N= 4,444,932 participants	Autoimmune connective tissue diseases	Cumulative incidence for events reported/10^4 person-years.<br>Hazard ratios<br><br>**Follow-up of at least 365 days**	Yes:<br>secondary analysis by gender, age group, type of vaccine
Kim et al., 2024 (12)<br>Republic of Korea (South Korea- Seoul)<br><br>Retrospective cohort	•BNT162b2 mRNA<br>•mRNA-1273<br>•AZD1222<br>•JNJ-78436735	National Health Insurance service database adult participants ≥ 20 years (January 1st, 2020 to December 31st, 2021)<br><br>N = 2,086,709 participants (all vaccines)	**Historical (pre-vaccine) and concurrent:** National Health Insurance service database adult participants ≥ 20 years<br>(January 1st, 2020 to December 31st, 2021)	Autoimmune adverse events	Cumulative incidence for adverse events reported/10^4 persons;<br>Hazard ratios for autoimmune adverse events	Yes:<br>subgroup analysis by sex, age, vaccine type, diabetes mellitus, hypertension, hyperlipemia, chronic obstructive pulmonary

Funding: None		**•BNT162b2 mRNA**<br>1st dose: 1,172,254 participants<br>2nd dose: 1,308,228 participants<br>**•mRNA-1273**<br>1st dose: 30,277 participants<br>2nd dose: 30,305 participants	N = 343,311 particpants		**1 week, 2 weeks, 1 month and 3 months restriction after vaccination**	disease
Li et al., 2022 (13)<br>China<br>Retrospective cohort<br><br>Funding: Public (Food and Health Bureau, HKSAR)	•BNT162b2 mRNA<br>•CoronaVac	Hong Kong vaccination records of all residents ≥16 years.<br>(February 23rd, 2021 to June 30th, 2021)<br><br>3.9 million Hong Kong residents<br>**•BNT162b2**<br>1st dose: 579,998 participants<br>2nd dose: 388,881 participants	**Historical (pre-vaccine) and concurrent:** electronic medical record from the Hospital Authority of all residents ≥16 years.<br>(January 1st, 2018 to June 30th, 2021)<br><br>1st dose: 2,816,133 participants<br>2nd dose: 1,892,783 participants	Hospital admission related to 16 pre-specifiedautoimmune diseases	Age standardized incidence rates reported/10^5 person-years;<br>Computation of age standardized incidence ratios.<br><br>**28 days after 1st or 2nd dose**	Yes:<br>secondary analysis without 28 days restriction after vaccine dose, sex-stratified and age standardized for each dose
Shani M, 2024 (14)<br>Israel<br>Retrospective cohort<br>Funding: None	•BNT162b2 mRNA	CHS members aged 12 years or older<br>(December 2020 to December 2021)<br><br>two doses of BNT162b2 mRNA<br>N= 2,455,207 participants	**Concurrent:** CHS members aged 12 years or older unvaccinated<br>(December 2020 to December 2021)<br><br>N = 594,879	24 immune-mediated diagnoses	Cumulative incidence rates reported/10^5person-years.<br><br>**4 to 12 months after vaccination**	Yes:<br>subgroup analysis based on age intervals (12–17,<br>18–44, 45–64, 65 years or older)

Abbreviations: CDC: Center of Disease Control; GCA: giant cell arteritis; PMR: polymyalgia rheumatica; VAERS: Vaccine adverse event reporting System.

Table S5. Confounding variables considered across the included studies

Author, year	Previous SARS-CoV-2 Infection	Non-mRNA Vaccination	Vaccine Dose Restriction	Autoimmune Comorbidities Restriction	Adjusted For Variables	Age
**Pharmacovigilance (Disproportionality analyses)**						
Fraenza et al., 2025 (1)	Not reported	Not reported	Not reported	Not reported	Subgroup analysis with or without underling rheumatic disease	(2.2%) was of the pediatric patients (aged between 2 months and 17 years)
Jarrot et al., 2024 (2)	Not reported	No	No<br>(stratified analysis for vaccine dose)	No<br>(excluded only positive ANCA)	Not applicable	Age ≥ 50 years
Mettler et al., 2021 (3)	Not reported	Not reported	Not reported	Not reported	Not applicable	Adults<br>(median 71.5, IQR: 64–78)
Mettler et al., 2022 (4)	Not reported	Not reported	Not reported	Not reported	Not applicable	Adults<br>(median 58 years, IQR: 38–72)
Nakao et al., 2023 (5)	Not reported	Not reported	Dose-specific effects were not analyzed	Not reported	Not applicable	Also individuals < 20 years (9/30 IgAN cases)
Oh et al., 2024 (6)	Not reported	Not reported	Not reported	Not reported	Not applicable	All ages included<br>(median 51 years)
Xie et al., 2026 (7)	Not reported	Included non-mRNA vaccines as comparators	Not reported	excluded PTs containing 'lupus' that were not associated with lupus disease and excluded history/current illness with 'lupus'.	None	All ages included<br>(NR for mRNA)
**Population-based comparative studies**						
Bøås et al., 2026 (8)<br>Norway	Analysis adjusted for previous SARS-CoV-2 infection.<br>Separate SARS-CoV-2 infection analysis reported.	Censored at non-mRNA COVID-19 vaccination.	Yes (risk periods after doses 1-3; censored at 4th vaccine dose. No dose-specific effect estimates reported.)	Yes (3-year washout period (2017-2020) to exclude prevalent cases. Incident outcome required first diagnosis followed by same diagnosis within 182 days.)	Age, sex, health region, municipality size, household size, household crowding, low family income, country of origin. Vaccination analysis additionally adjusted for previous COVID infection.	Adults 18-64 years.<br>Age-stratified: 18-39 and 40-65 years.
El Sahly et al., 2021 (9)<br>USA	Excluded participants with prior infection	No	No	No	Person-months (total time at risk) in each treatment group	Adults (≥18 years)

Ju et al., 2023 (10) Republic of Korea (South Korea)	Individuals with any evidence of SARS-CoV-2 infection before December 31, 2021, were excluded	Subgroup analyses based on history of non-mRNA vaccination prior to mRNA vaccination	No (included individuals who received at least one dose of mRNA vaccine)	No (adjusted for Hashimoto thyroiditis)	18 covariates, including demographic, socioeconomic, and comorbidity factors	Adults and individuals <20 years (< 10%)
Jung et al., 2024 (11) Republic of Korea (South Korea)	Stratified participants based on prior COVID-19 diagnosis	Stratified analyis with only mRNA vaccination and non-mRNA cross vaccination	No (stratified analysis for booster vaccination)	No (adjusted for Hashimoto thyroiditis)	Demographics and socioeconomic status (age, sex, insurance type), income level, area of residence (urban vs rural area), and general health (predefined underlining disease, drinking and smoking status)	Adults (≥ 18 years)
Kim et al., 2024 (12) Seoul, South Korea	Additional analysis for individuals with previous COVID-19	Secondary analyis with only mRNA vaccination and non-mRNA cross vaccination	Yes (excluded vaccination only one time)	Excluded from both grups	Not applicable	≥ 20 years
Li et al., 2022 (13) China	Additional analysis excluding individuals who had tested positive for SARS-CoV-2	Stratified analyis with only mRNA vaccination	No (both doses analysed)	Mentioned but not excluded from analysis ”vaccine recipients were less likely to have preexisting chronic diseases”	Not applicable (age-standardized incidence rate ratios and age-sex matching)	≥16 years (age-standardization using 5 year age bands.)
Shani et al., 2024 (14) Israel	Before vaccine excluded<br><br>In the case of a positive SARS-CoV-2 test during the follow-up period, participation was censored.	Only mRNA vaccination	Yes (at least one dose, but most analyses focus on those who received two doses)	Excluded	Age, sex, population sector, score for socioeconomic status, rate of peripheral residency, obesity, smoking, previous diagnoses with any immune-mediated disease, hypertension, hyperlipidemia, active malignant disease, depression and anxiety.	≥12 years (outcomes of interest for our study reported for ≥18)

Table S6. Evidence map of assessed outcomes across included studies

Disproportionality studies								Pathology and vaccine type	Population-based comparative studies							
Fraenza (1)	Jarrot (2)	Mettler (3)	Mettler (4)	Nakao (5)	Oh (6)	Xie (7)	No		No	Bøås (8)	El Sahly (9)	Ju et al., (10)	Jung (11)	Kim (12)	Li (13)	Shani (14)
overall mRNA								Vaccine type				BNT162b2 mRNA				
										mRNA-1273						
			x				1	ANCA-associated vasculitis	2			x	x			
			x				1	Ankylosing spondylitis	3			x	x	x		
							0	Antiphospholipid syndrome	1						x	
			x				1	Bechet syndrome	2			x	x			
			x				1	Mixed connective tissue disease	0							
			x				1	Cryoglobulinaemic vasculitis	0							
			x				1	Eosinophilic granulomatosis with polyangiitis	0							
	x	x	x				3	Giant cell arteritis	1							x
			x				1	Granulomatosis with polyangiitis	0							
			x	x			2	Henoch-Schönlein purpura	0							
			x				1	Microscopic polyangiitis	0							
							0	Poly/Dermatomyositis	3	x		x	x			
	x	x	x		x		4	Polymyalgia rheumatica	2		x					x
			x				1	Polyarteritis nodosa	0							
							0	Psoriatic arthritis	1						x	
							0	Reactive arthritis	1						x	
							0	Renal vasculitis	0							
					x		1	Rheumatoid arthritis	7	x	x	x	x	x	x	x
					x		1	Sjogren’s syndrome	6	x		x	x	x	x	x
							0	Spondyloarthritis	2	x					x	
x					x	x	3	Systemic lupus erythematosus	5	x		x	x	x	x	
				x		x	2	Lupus nephritis	0							
					x		1	Systemic sclerosis	3	x		x	x			
			x				1	Takayasu’s arteritis	0							
Eudra Vigilance	France PV database	VigiBase	VigiBase	JADER	VigiBase	VAERS		**Database or country**		Norway	USA	South Korea	South Korea	Seoul, South Korea	China	Israel

Section S5. Pharmacovigilance (Disproportionality analyses): Reporting of New-Onset Autoimmune Inflammatory Rheumatic Diseases

Table S7. Potential signals within disproportionality analyses

Autoimmune disease	mRNA[a] vs.		Tozinameran (BNT162b2, Pfizer) vs.		Elasomeran (mRNA-1273, Moderna) vs.	Author, year
	All other drugs	Influenza vaccine	Elasomeran (mRNA-1273)	Heterologous vaccination	Heterologous vaccination	
ANCA associated vasculitis	0.9 (0.8-1.0)	0.4 (0.3-0.4)	1.8 (1.3-2.5)	NR	NR	Mettler et al., 2022 (4)
ANCA positive vasculitis	0.9 (0.7-1.1)	0.3 (0.2- 0.5)	1.5 (0.8-2.7)	NR	NR	Mettler et al., 2022 (4)
Ankylosing spondylitis	1.44 (1.32-1.56)	NR	NR	NR	NR	Oh et al., 2024 (6)
Behcet syndrome	1.7 (1.4-2.1)	4.2 (1.3-13.2)	1.7 (1.0-2.9)	NR	NR	Mettler et al., 2022 (4)
Cryoglobulinemic vasculitis	1.2 (0.8-1.6)	0.6 (0.3-1.2)	NR[c]	NR	NR	Mettler et al., 2022 (4)
Eosinophilic granulomatosis with polyangiitis	0.5 (0.4-0.6)	0.4 (0.2-0.7)	1.5 (0.8-2.9)	NR	NR	Mettler et al., 2022 (4)
Giant cell arteritis	3.2 (2.5-4.1)	0.6 (0.4-0.8)	NR	NR	NR	Mettler et al., 2021 (3)
	4.5 (4.0-5.0)	0.7 (0.6-0.9)	1.6 (1.3-1.9)	NR	NR	Mettler et al., 2022 (4)
Giant cell arteritis and Polymyalgia rheumatica	3.2 (1.4-7.3)	0.3 (0.1-0.5)	NR	NR	NR	Mettler et al., 2021 (3)
Granulomatosis with polyangiitis	1.3 (1.0-1.7)	0.3 (0.2-0.4)	1.6 (0.9-2.7)	NR	NR	Mettler et al., 2022 (4)
IgA vasculitis (Henoch Schonlein purpura)	0.7 (0.6-0.8)	0.1 (0.1-0.1)	1.5 (1.1-2.1)	NR	NR	Mettler et al., 2022 (4)
IgA vasculitis (nephropathy)	6.49 (4.38-9.61)	NR	NR	NR	NR	Nakao et al., 2023 (5)
Microscopic polyangiitis	2.6 (1.8-3.7)	0.5 (0.2-0.9)	4.0 (1.2-13.0)	NR	NR	Mettler et al., 2022 (4)
Polyarteritis nodosa	0.4 (0.2-0.5)	0.3 (0.1-0.5)	1.5 (0.5-3.9)	NR	NR	Mettler et al., 2022 (4)
Polymyalgia rheumatica	4.1 (3.6-4.7)	0.4 (0.4-0.5)	NR	NR	NR	Mettler et al., 2021 (3)
	34.98 (32.63-37.49)	NR	NR	NR	NR	Oh et al., 2024 (6)
Rheumatoid arthritis	1.99 (1.93-2.05)	NR	NR	NR	NR	Oh et al., 2024 (6)
Sjogren's syndrome	2.92 (2.65-3.23)	NR	NR	NR	NR	Oh et al., 2024 (6)
Systemic lupus erythematosus	NR	NR	0.87 (0.80-0.95)	1.63 (1.18-2.26)	1.88 (1.35-2.61)	Fraenza et al., 2025 (1)
	1.79 (1.68-1.91)	NR	NR	NR	NR	Oh et al., 2024 (6)
	0.62 (0.56–0.69)[b]	NR	NR	NR	NR	Xie et al., 2026 (7)
Systemic lupus erythematosus (nephritis)	0.59 (0.48-0.72)	NR	NR	NR	NR	Nakao et al., 2023 (5)
	0.65 (0.41–1.03)[b]	NR	NR	NR	NR	Xie et al., 2026 (7)
Systemic sclerosis	3.55 (2.76-4.58)	NR	NR	NR	NR	Oh et al., 2024 (6)
Takayasu arteritis	0.5 (0.3-1.0)	NR[c]	NR[c]	NR	NR	Mettler et al., 2022 (4)

Disproportionality results are expressed as reporting odds ratios (ROR) with 95% confidence intervals. Disproportionality measures do not estimate incidence or relative risk.
Overall mRNA refers to both BNT 162b2 (Pfizer) and mRNA-1273 (Moderna) vaccination. Disproportionality estimates depend on the reference set and are not directly comparable across columns.
Cell colors indicate statistically significant disproportionality of reporting: Lower reporting (significant), Higher-than-expected reporting (significant), No disproportionality detected

[a]mRNA as overall mRNA-1273 and BNT162b2.
[b]Reference set: all other reports in VAERS (vaccine-only database)- the "All other drugs" reference set varies by source database.
[c]insufficient cases observed with the studied drugs.
Abbreviation: mRNA: messenger Ribonucleic Acid, NR: not reported.

Table S8. Dose stratified analyses

Autoimmune disease	Dose	Incidence vaccinated		Unvaccinated	Incidence unvaccinated	Reported effect size aHR (95%CI)/IRR[d]*	Author, year
		BNT 162b2	mRNA-1273				
Giant cell arteritis	overall doses	0.77	0.5	Literature data	NR	”lower incidence after COVID-19 vaccination”	Jarrot et al. (2024) (2)
	1st	$1.03/10^6$ doses	$0.73/10^6$ doses	Literature data	NR		
	2 [nd]	$0.73/10^6$ doses	$0.60/10^6$ doses	Literature data	NR		
	3rd	$0.53/10^6$ doses	$0.34/10^6$ doses	Literature data	NR		
Polymyalgia rheumatica	overall doses	2.53	1.84	Literature data	NR	”lower incidence after COVID-19 vaccination”	Jarrot et al. (2024) (2)
	1st	$2.11/10^6$ doses	$2.56/ 10^6$ doses	Literature data	NR		
	2 [nd]	$3.64/10^6$ doses	$2.98/10^6$ doses	Literature data	NR		
	3rd	$1.71/10^6$ doses	$0.86/10^6$ doses	Literature data	NR		

Table S9. Age stratified analyses

	**IC (IC0.25) based on age for mRNA vaccine vs. all medications** Oh J et al. 2024 (6)		
**Autoimmune disease**	**0-17**	**18-64**	**≥65**
Ankylosing spondylitis	1.40 (-2.38)	0.51 (0.34)*	1.59 (1.09)*
Polymyalgia rheumatica	2.76 (0.69)*	3.61 (3.49)*	4.38 (4.30)*
Rheumatoid arthritis	3.22 (2.49)*	0.03 (-0.04)	1.12 (1.02)*
Sjogren’s syndrome	2.63 (0.56)*	1.46 (1.27)*	0.91 (0.50)*
Systemic lupus erythematosus	4.68 (4.10)*	0.20 (0.07)*	1.20 (0.91)*
Systemic sclerosis	N/A	-1.13 (-1.62)	-1.39 (-2.33)

*considered statistically significant values (the value of IC0.25 is greater than 0.0 or the lower end of the ROR 95% CI greater than 1.0). Negative IC values indicate reporting below expected levels.
N/A = not available due to insufficient case counts.

Section S6. Population-based comparative studies: Incidence of New-Onset Autoimmune Inflammatory Rheumatic Diseases

Table S10. Overall mRNA vaccines

Autoimmune disease	Overall mRNA vaccine dose	Incidence vaccinated	Unvaccinated group from historical (pre-vaccine) [a]/ concurrent [b] cohort/ both periods [c]	Incidence unvaccinated	Reported effect size HR [c]/aHR[d](95%CI)	Author, year
ANCA- associated vasculitis	≥ 1	0.7/10^5	up to 21st December 2020 [a]	0.5/10^5	1.36 (0.60-3.08)[d]	Ju et al. (2023), Republic of Korea (10)
	≥ 1	1.0/10^5	up to 31st December 2020 [a]	0.9/10^5	1.09 (0.72-1.66) [d]	Jung et al. (2024), Republic of Korea (11)
Behcet disease	≥ 1	3.2/10^5	up to 21st December 2020 [a]	3.0/10^5	1.06 (0.75-1.50) [d]	Ju et al. (2023), Republic of Korea (10)
	≥ 1	4.8/10^5	up to 31st December 2020 [a]	6.6/10^5	0.69 (0.58-0.82) [d]	Jung et al. (2024), Republic of Korea (11)
Poly/Dermatomyositis	≥ 1	1.16/10^5	January 1st , 2017 onward [b]	1.13/10^5	0.56 (0.19-1.62)	Bøås et al. (2026), Norway (8)
	≥ 1	2.1/10^5	up to 21st December 2020 [a]	1.7/10^5	1.20 (0.77-1.88) [d]	Ju et al. (2023), Republic of Korea (10)
	≥ 1	2.2/10^5	up to 31st December 2020 [a]	2.0/10^5	1.02 (0.77-1.35) [d]	Jung et al. (2024), Republic of Korea (11)
Rheumatoid arthritis	≥ 1	28.52/10^5	January 1st , 2017 onward [b]	26.32/10^5	1.11 (0.90-1.37)	Bøås et al. (2026), Norway (8)
	≥ 1	111.1/10^5	up to 21st December 2020 [a]	114.9/10^5	0.92 (0.87-0.98) [d]	Ju et al. (2023), Republic of Korea (10)
	≥ 1	209/10^5	up to 31st December 2020 [a]	220/10^5	0.86 (0.84-0.89) [d]	Jung et al. (2024), Republic of Korea (11)
	≤ 3rd	NR	January 1st, 2020 to December 31st, 2021[c]	NR	0.98 (0.65-1.47)[c]	Kim et al. (2024), Seoul-South Korea (12)
Sjogren's syndrome	≥ 1	6.28/10^5	January 1st , 2017 onward [b]	5.37/10^5	1.20 (0.78-1.85)	Bøås et al. (2026), Norway (8)
	≥ 1	9.6/10^5	up to 21st December 2020 [a]	9.8/10^5	0.90 (0.74-1.10) [d]	Ju et al. (2023), Republic of Korea (10)
	≥ 1	17.4/10^5	up to 31st December 2020 [a]	14.5/10^5	1.07 (0.96-1.18) [d]	Jung et al. (2024), Republic of Korea (11)
	≤ 3rd	NR	January 1st, 2020 to December 31st, 2021[c]	NR	1.02 (0.54-1.93) [c]	Kim et al. (2024), Seoul- South Korea (12)
Spondyloarthritis (* only for ankylosing spondylitis)	≥ 1	2.15/10^5	January 1st , 2017 onward [b]	3.58/10^5	0.48 (0.23-0.99)	Bøås et al. (2026), Norway (8)
	≥ 1*	15.2/10^5	up to 21st December 2020 [a]	14.9/10^5	1.00 (0.85-1.17) [d]	Ju et al. (2023), Republic of Korea (10)
	≥ 1*	22.6/10^5	up to 31st December 2020 [a]	22.4/10^5	0.95 (0.87-1.04) [d]	Jung et al. (2024), Republic of Korea (11)
	≤ 3rd*	NR	January 1st, 2020 to December 31st, 2021[c]	NR	0.79 (0.46-1.37) [c]	Kim et al. (2024), Seoul- South Korea (12)
Systemic lupus erythematosus	≥ 1	2.64/10^5	January 1st , 2017 onward [b]	2.53/10^5	1.30 (0.61-2.75)	Bøås et al. (2026), Norway (8)
	≥ 1	4.6/10^5	up to 21st December 2020 [a]	6.0/10^5	0.75 (0.58-0.99) [d]	Ju et al. (2023), Republic of Korea (10)
	≥ 1	11.3/10^5	up to 31st December 2020 [a]	9.1/10^5	1.16 (1.02-1.32) [d]	Jung et al. (2024), Republic of Korea (11)

	≤ 3rd	NR	January 1st, 2020 to December 31st, 2021[c]	NR	0.62 (0.30-1.29) [c]	Kim et al. (2024), Seoul- South Korea (12)
Systemic sclerosis	≥ 1	$2.15/10^5$	January 1st, 2017 onward [b]	$2.07/10^5$	1.06 (0.51-2.22)	Bøås et al. (2026), Norway (8)
	≥ 1	$1.1/10^5$	up to 21st December 2020 [a]	$1.2/10^5$	0.82 (0.46-1.45) [d]	Ju et al. (2023), Republic of Korea (10)
	≥ 1	$1.8/10^5$	up to 31st December 2020 [a]	$1.6/10^5$	1.01 (0.73-1.38) [d]	Jung et al. (2024), Republic of Korea (11)

Cell color legend indicating incidence in vaccinated compared to unvaccinated: Reduced risk (significant), Increased risk (significant), No association / neutral
Overall mRNA refers to both BNT 162b2 (Pfizer) and mRNA-1273 (Moderna) vaccination.

Table S11. BNT162b2 mRNA (Pfizer) vaccination

Autoimmune disease	BNT 162b2 dose	Incidence vaccinated	Unvaccinated group from historical (pre-vaccine) [a]/ concurrent [b] cohort / both periods [c]	Incidence unvaccinated	Reported effect size aHR (95%CI)/IRR[d]*	Author, year
ANCA- associated vasculitis	≥ 1	NR	up to 21st December 2020 [a]	NR	1.54 (0.68-3.50)	Ju et al. (2023), Republic of Korea (10)
	≥ 1	NR	up to 31st December 2020 [a]	NR	1.05 (0.65-1.69)	Jung et al. (2024), Republic of Korea (11)
Anti-phospholipid antibody syndrome	1st	$0.27/10^5$	January 2018 to June 2021[c]	$0.19/10^5$	1.42* (NR)	Li et al. (2022), China (13)
	2 nd	$0/10^5$	January 2018 to June 2021[c]	$0.32/10^5$	0* (NR)	
Behcet disease	≥ 1	NR	up to 21st December 2020 [a]	NR	1.08 (0.75-1.55)	Ju et al. (2023), Republic of Korea (10)
	≥ 1	NR	up to 31st December 2020 [a]	NR	0.75 (0.62-0.91)	Jung et al. (2024), Republic of Korea (11)
Poly/Dermatomyositis	≥ 1	NR	up to 21st December 2020 [a]	NR	1.29 (0.81-2.04)	Ju et al. (2023), Republic of Korea (10)
	≥ 1	NR	up to 31st December 2020 [a]	NR	0.99 (0.72-1.35)	Jung et al. (2024), Republic of Korea (11)
Psoriatic arthritis	1st	$0.26/10^5$	January 2018 to June 2021[c]	$0.38/10^5$	0.68* (NR)	Li et al. (2022), China (13)
	2 nd	$0.20/10^5$	January 2018 to June 2021[c]	$0.37/10^5$	0.54* (NR)	
Reactive arthritis	1st	$4.12/10^5$	January 2018 to June 2021[c]	$8.49/10^5$	0.48* (NR)	Li et al. (2022), China (13)
	2 nd	$7.24/10^5$	January 2018 to June 2021[c]	$8.56/10^5$	0.84* (NR)	
Rheumatoid arthritis	≥ 1	NR	up to 21st December 2020 [a]	NR	0.96 (0.90-1.02)	Ju et al. (2023), Republic of Korea (10)
	≥ 1	NR	up to 31st December 2020 [a]	NR	0.88 (0.85-0.91)	Jung et al. (2024), Republic of Korea (11)
	1st	$0.34/10^5$	January 2018 to June 2021[c]	$1.60/10^5$	0.21* (NR)	Li et al. (2022), China (13)
	2 nd	$0.36/10^5$	January 2018 to June 2021[c]	$1.69/10^5$	0.21* (NR)	
Sjogren's syndrome	≥ 1	NR	up to 21st December 2020 [a]	NR	0.86 (0.70-1.05)	Ju et al. (2023), Republic of Korea (10)
	≥ 1	NR	up to 31st December 2020 [a]	NR	1.06 (0.95-1.19)	Jung et al. (2024), Republic of Korea (11)
	1st	$1.14/10^5$	January 2018 to June 2021[c]	$0.13/10^5$	8.76* (NR)	Li et al. (2022), China (13)
	2 nd	$0/10^5$	January 2018 to June 2021[c]	$0.12/10^5$	0* (NR)	
Spondyloarthritis (* only for ankylosing spondylitis)	≥ 1*	NR	up to 21st December 2020 [a]	NR	1.08 (0.91-1.27)	Ju et al. (2023), Republic of Korea (10)
	≥ 1*	NR	up to 31st December 2020 [a]	NR	1.02 (0.92-1.12)	Jung et al. (2024), Republic of Korea (11)
	1st	$0.14/10^5$	January 2018 to June 2021[c]	$0.40/10^5$	0.35* (NR)	Li et al. (2022), China (13)
	2 nd	$0/10^5$	January 2018 to June 2021[c]	$0.42/10^5$	0* (NR)	
Systemic lupus erythematosus	≥ 1	NR	up to 21st December 2020 [a]	NR	0.72 (0.54-0.96)	Ju et al. (2023), Republic of Korea (10)
	≥ 1	NR	up to 31st December 2020 [a]	NR	1.18 (1.02-1.36)	Jung et al. (2024), Republic of Korea (11)
	1st	$0.15/10^5$	January 2018 to June 2021[c]	$1.21/10^5$	0.12* (NR)	Li et al. (2022), China (13)
	2 nd	$0.20/10^5$	January 2018 to June 2021[c]	$1.21/10^5$	0.16* (NR)	
Systemic sclerosis	≥ 1	NR	up to 21st December 2020 [a]	NR	1.03 (0.58-1.83)	Ju et al. (2023), Republic of Korea (10)
	≥ 1	NR	up to 31st December 2020 [a]	NR	1.06 (0.75-1.49)	Jung et al. (2024), Republic of Korea (11)

[d]*self-calculated IRR values

Cell color legend indicating incidence in vaccinated compared to unvaccinated: Reduced risk (significant), Increased risk (significant), No association / neutral

Table S12. mRNA-1273 (Moderna) vaccination

Autoimmune disease	mRNA-1273 dose	Incidence vaccinated	Unvaccinated group from historical (pre-vaccine) [a]/ concurrent [b] cohort	Incidence unvaccinated	Reported effect size aHR (95%CI)	Author, year
ANCA- associated vasculitis	≥ 1	NR	up to 31st December 2020 [a]	NR	1.25 (0.69-2.25)	Jung et al. (2024), Republic of Korea (11)
Behcet disease	≥ 1	NR	up to 21st December 2020 [a]	NR	0.92 (0.49-1.71)	Ju et al. (2023), Republic of Korea (10)
	≥ 1	NR	up to 31st December 2020 [a]	NR	0.53 (0.39-0.72)	Jung et al. (2024), Republic of Korea (11)
Poly/Dermatomyositis	≥ 1	NR	up to 21st December 2020 [a]	NR	1.04 (0.42-2.54)	Ju et al. (2023), Republic of Korea (10)
	≥ 1	NR	up to 31st December 2020 [a]	NR	1.11 (0.75-1.65)	Jung et al. (2024), Republic of Korea (11)
Polymyalgia rheumatica	2	$0/10^5$	Placebo (July to October 2020[b])	$6.59/10^5$	NR	El Sahly et al. (2021), USA (9)
Rheumatoid arthritis	2	$6.58/10^5$	Placebo (July to October 2020[b])	$0/10^5$	NR [1]	El Sahly et al. (2021), USA (9)
	≥ 1	NR	up to 21st December 2020 [a]	NR	0.75 (0.67-0.85)	Ju et al. (2023), Republic of Korea (10)
	≥ 1	NR	up to 31st December 2020 [a]	NR	0.81 (0.78-0.85)	Jung et al. (2024), Republic of Korea (11)
Sjogren's syndrome	≥ 1	NR	up to 21st December 2020 [a]	NR	1.18 (0.83-1.67)	Ju et al. (2023), Republic of Korea (10)
	≥ 1	NR	up to 31st December 2020 [a]	NR	1.05 (0.90-1.22)	Jung et al. (2024), Republic of Korea (11)
Spondyloarthritis (* only for ankylosing spondylitis)	≥ 1*	NR	up to 21st December 2020 [a]	NR	0.68 (0.50-0.93)	Ju et al. (2023), Republic of Korea (10)
	≥ 1*	NR	up to 31st December 2020 [a]	NR	0.81 (0.71-0.93)	Jung et al. (2024), Republic of Korea (11)
Systemic lupus erythematosus	≥ 1	NR	up to 21st December 2020 [a]	NR	0.78 (0.48-1.25)	Ju et al. (2023), Republic of Korea (10)
	≥ 1	NR	up to 31st December 2020 [a]	NR	1.08 (0.89-1.32)	Jung et al. (2024), Republic of Korea (11)
Systemic sclerosis	≥ 1	NR	up to 31st December 2020 [a]	NR	0.89 (0.54-1.45)	Jung et al. (2024), Republic of Korea (11)

*self-calculated

Cell color legend indicating incidence in vaccinated compared to unvaccinated: Reduced risk (significant), Increased risk (significant), No association / neutral

[1] self-calculated effect estimate (RR (95%CI): 0.732 to 230.722) for vaccinated vs placebo showed no difference between groups.

Table S13. Gender stratified analysis

Stratified analyses of the risks of incident autoimmune disorders in the mRNA-based COVID-19 vaccination cohort compared with the control cohort according to gener.

Autoimmune disease	Vaccine type	Dose	Unvaccinated group from historical (pre-vaccine) [a]/ concurrent [b] cohort/ both periods[c]	Male aHR[a]/IRR[b] (95%CI)	Female aHR[a]/IRR[b] (95%CI)	Author, year
ANCA-associated vasculitis	overall mRNA	≥ 1	up to 31st December 2020 [a]	1.01 (0.52–1.94) [a]	1.15 (0.66–2.00) [a]	Jung et al. (2024), Republic of Korea (11)
Anti-phospholipid antibody syndrome	BNT 162b2	1st	January 2018 to June 2021[c]	3.48 (0.41-23.41)[b]	0 (0-11.06) [b]	Li et al. (2022), China (13)
		2nd	January 2018 to June 2021[c]	0 (0-15.64) [b]	0 (0-4.46) [b]	
Behcet disease	overall mRNA	≥ 1	up to 21st December 2020 [a]	0.74 (0.4-1.37) [a]	1.26 (0.83-1.92) [a]	Ju et al. (2023),Republic of Korea (10)
	overall mRNA	≥ 1	up to 31st December 2020 [a]	0.62 (0.46–0.82) [a]	0.74 (0.59–0.92) [a]	Jung et al. (2024), Republic of Korea (11)
Poly/Dermatomyositis	overall mRNA	≥ 1	up to 21st December 2020 [a]	1.36 (0.73-2.54) [a]	1.06 (0.56-2.00) [a]	Ju et al. (2023), Republic of Korea (10)
	overall mRNA	≥ 1	up to 31st December 2020 [a]	0.83 (0.55–1.23) [a]	1.25 (0.84–1.85) [a]	Jung et al. (2024), Republic of Korea (11)
Psoriatic arthritis	BNT 162b2	1st	January 2018 to June 2021[c]	0.75 (0.03-4.82) [b]	0 (0-4.18) [b]	Li et al. (2022), China (13)
		2nd	January 2018 to June 2021[c]	0 (0-5.02) [b]	1.09 (0.05-7.84) [b]	
Reactive arthritis	BNT 162b2	1st	January 2018 to June 2021[c]	0.48 (0.27-0.81) [b]	0.44 (0.25-0.74) [b]	Li et al. (2022), China (13)
		2nd	January 2018 to June 2021[c]	0.67 (0.38-1.10) [b]	1.21 (0.82-1.75) [b]	
Rheumatoid arthritis	overall mRNA	≥ 1	up to 21st December 2020 [a]	0.94 (0.86-1.04) [a]	0.91 (0.84-0.98) [a]	Ju et al. (2023), Republic of Korea (10)
	overall mRNA	≥ 1	up to 31st December 2020 [a]	0.88 (0.84–0.92) [a]	0.85 (0.82–0.88) [a]	Jung et al. (2024), Republic of Korea (11)
	BNT 162b2	1st	January 2018 to June 2021[c]	0 (0-1.35) [b]	0.43 (0.11-1.23) [b]	Li et al. (2022), China (13)
		2nd	January 2018 to June 2021[c]	0 (0-1.78) [b]	0.19 (0.01-0.98) [b]	
Systemic lupus erythematosus	overall mRNA	≥ 1	up to 21st December 2020 [a]	0.52 (0.31-0.88) [a]	0.86 (0.63-1.18) [a]	Ju et al. (2023), Republic of Korea (10)
	overall mRNA	≥ 1	up to 31st December 2020 [a]	1.23 (0.94–1.60) [a]	1.13 (0.98–1.31) [a]	Jung et al. (2024), Republic of Korea (11)
	BNT 162b2	1st	January 2018 to June 2021[c]	0 (0-8.95) [b]	0.21 (0.01-1.09) [b]	Li et al. (2022), China (13)
		2nd	January 2018 to June 2021[c]	0 (0-7.72) [b]	0.27 (0.01-1.47) [b]	
Systemic sclerosis	overall mRNA	≥ 1	up to 21st December 2020 [a]	1.44 (0.59-3.54) [a]	0.54 (0.25-1.18) [a]	Ju et al. (2023), Republic of Korea (10)
	overall mRNA	≥ 1	up to 31st December 2020 [a]	1.30 (0.63–2.69) [a]	0.94 (0.66–1.34) [a]	Jung et al. (2024), Republic of Korea (11)

Sjogren syndrome	overall mRNA	≥ 1	up to 21st December 2020 [a]	0.92 (0.59-1.43) [a]	0.90 (0.72-1.11) [a]	Ju et al. (2023), Republic of Korea (10)
	overall mRNA	≥ 1	up to 31st December 2020 [a]	1.12 (0.90–1.41) [a]	1.05 (0.93–1.18) [a]	Jung et al. (2024), Republic of Korea (11)
	BNT 162b2	1st	January 2018 to June 2021[c]	NA (0-inf) [b]	1.61 (0.07-12.84) [b]	Li et al. (2022), China (13)
		2nd	January 2018 to June 2021[c]	NA (0- inf) [b]	0 (0-9.31) [b]	
Spondyloarthritis (* only for ankylosing spondylitis)	overall mRNA*	≥ 1	up to 21st December 2020 [a]	1.11 (0.90-1.36) [a]	0.87 (0.68-1.10) [a]	Ju et al. (2023), Republic of Korea (10)
	overall mRNA*	≥ 1	up to 31st December 2020 [a]	0.96 (0.85–1.08) [a]	0.94 (0.83–1.07) [a]	Jung et al. (2024), Republic of Korea (11)
	BNT 162b2	1st	January 2018 to June 2021[c]	0.52 (0.02- 3.11)	0 (0-22.40)	Li et al. (2022), China (13)
		2nd	January 2018 to June 2021[c]	0 (0-2.41)	0 (0-103.15)	

Overall mRNA refers to both BNT 162b2 (Pfizer) and mRNA-1273 (Moderna) vaccination
Cell color legend indicating incidence in vaccinated compared to unvaccinated: Reduced risk (significant), Increased risk (significant), No association / neutral

Table S14. Age stratified analysis

Stratified analyses of the risks of incident autoimmune disorders in the mRNA-based COVID-19 vaccination cohort compared with the unvaccinated cohort according to age.

Autoimmune disease	Vaccine	Dose	Unvaccinated group from historical (pre-vaccine) [a]/ concurrent [b] cohort	< 40 years aHR (95%CI)	≥ 40 years aHR (95%CI)	45 - 64 years aHR (95%CI)	> 65 years aHR (95%CI)	Author, year
ANCA-associated vasculitis	overall mRNA	≥ 1	up to 31st December 2020 [a]	0.79 (0.19–3.28)	1.12 (0.72–1.75)	NR	NR	Jung et al. (2024), Republic of Korea (11)
Behcet disease	overall mRNA	≥ 1	up to 21st December 2020 [a]	0.58 (0.33-1.03)	1.55 (0.99-2.42)	NR	NR	Ju et al. (2023), Republic of Korea (10)
	overall mRNA	≥ 1	up to 31st December 2020 [a]	0.69 (0.46–1.05)	0.68 (0.56–0.83)	NR	NR	Jung et al. (2024), Republic of Korea (11)
Giant cell arteritis	BNT 162b2	2nd	December 2020 to December 2021 [b]	NR	NR	NR	1.88 (0.82–4.3)	Shani et al. (2024), Israel (14)
Polymyalgia rheumatica	BNT 162b2	2nd	December 2020 to December 2021 [b]	NR	NR	NR	2.12 (1.3–3.47)	Shani et al. (2024), Israel (14)
Poly/Dermatomyositis	overall mRNA	≥ 1	January 1st, 2017 onward [b]	1.73 (0.43-7.02)	0.26 (0.06-1.19)	NR	NR	Bøås et al. (2026), Norway (8)
	overall mRNA	≥ 1	up to 21st December 2020 [a]	0.66 (0.18-2.33)	1.32 (0.82-2.13)	NR	NR	Ju et al. (2023), Republic of Korea (10)
	overall mRNA	≥ 1	up to 31st December 2020 [a]	1.43 (0.55–3.71)	0.99 (0.73–1.32)	NR	NR	Jung et al. (2024), Republic of Korea (11)
Rheumatoid arthritis	overall mRNA	≥ 1	January 1st, 2017 onward [b]	0.84 (0.53-1.34)	1.15 (0.90-1.48)	NR	NR	Bøås et al. (2026), Norway (8)
	overall mRNA	≥ 1	up to 21st December 2020 [a]	0.93 (0.81-1.07)	0.92 (0.86-0.98)	NR	NR	Ju et al. (2023), Republic of Korea (10)
	overall mRNA	≥ 1	up to 31st December 2020 [a]	0.96 (0.87–1.06)	0.85 (0.83–0.88)	NR	NR	Jung et al. (2024), Republic of Korea (11)
	BNT 162b2	2nd	December 2020 to December 2021 [b]	NR	NR	1.21 (0.88–1.68)	1.26 (0.9–1.79)	Shani et al. (2024), Israel (14)
Systemic lupus erythematosus	overall mRNA	≥ 1	January 1st, 2017 onward [b]	0.87 (0.23-3.33)	1.50 (0.56-4.00)	NR	NR	Bøås et al. (2026), Norway (8)
	overall mRNA	≥ 1	up to 21st December 2020 [a]	0.56 (0.34-0.92)	0.86 (0.62-1.18)	NR	NR	Ju et al. (2023), Republic of Korea (10)
	overall mRNA	≥ 1	up to 31st December 2020 [a]	1.09 (0.79–1.50)	1.16 (1.00–1.33)	NR	NR	Jung et al. (2024), Republic of Korea (11)
Systemic sclerosis	overall mRNA	≥ 1	January 1st, 2017 onward [b]	0.34 (0.04-2.74)	1.30 (0.59-2.88)	NR	NR	Bøås et al. (2026), Norway (8)
	overall mRNA	≥ 1	up to 21st December 2020 [a]	2.16 (0.39-11.99)	0.72 (0.39-1.33)	NR	NR	Ju et al. (2023), Republic of Korea (10)
	overall mRNA	≥ 1	up to 31st December 2020 [a]	1.36 (0.41–4.51)	0.98 (0.71–1.36)	NR	NR	Jung et al. (2024), Republic of Korea (11)
Sjogren syndrome	overall mRNA	≥ 1	January 1st, 2017 onward [b]	0.91 (0.39-2.09)	1.22 (0.73-2.04)	NR	NR	Bøås et al. (2026), Norway (8)
	overall mRNA	≥ 1	up to 21st December 2020 [a]	1.27 (0.74-2.17)	0.86 (0.70-1.06)	NR	NR	Ju et al. (2023), Republic of Korea (10)
	overall mRNA	≥ 1	up to 31st December2020 [a]	0.97 (0.64–1.45)	1.07 (0.96–1.19)	NR	NR	Jung et al. (2024), Republic of Korea (11)

	BNT 162b2	2nd	December 2020 to December 2021 [b]	NR	NR	NR	1.05 (0.52–2.12)	Shani et al. (2024), Israel (14)
Spondyloarthritis (* only for ankylosing spondylitis)	overall mRNA	≥ 1	January 1st, 2017 onward [b]	0.34 (0.12-1.02)	0.60 (0.20-1.75)	NR	NR	Bøås et al. (2026), Norway (8)
	overall mRNA*	≥ 1	up to 21st December 2020 [a]	1.05 (0.81-1.36)	0.97 (0.80-1.18)	NR	NR	Ju et al. (2023), Republic of Korea (10)
	overall mRNA*	≥ 1	up to 31st December2020 [a]	1.15 (0.94–1.41)	0.91 (0.83–1.00)	NR	NR	Jung et al. (2024), Republic of Korea (11)

Overall mRNA refers to both BNT 162b2 (Pfizer) and mRNA-1273 (Moderna) vaccination
Cell color legend indicating incidence in vaccinated compared to unvaccinated: Reduced risk (significant), Increased risk (significant), No association / neutral

Among adults aged 18–65 years, risk windows were defined as 0–30 days and 30–365 days after vaccination. Hazard ratios were adjusted for age, sex, health region, municipality size, household size, household crowding, low family income, country of origin, and previous COVID-19 infection. For rheumatoid arthritis, the adjusted hazard ratio at 30–365 days was 1.30 (95% CI 1.10–1.54).- Bøås et al. (2026), Norway (8)

Table S15. COVID-19 diagnosis

	Incidence vaccinated			Incidence unvaccinated			Reported effect size before removing SARS-CoV-2 infection aHR(95%CI)[c]/ IRR*	Reported effect size after removing SARS-CoV-2 infection aHR(95%CI)[c]/ IRR*	Reported effect size in the subgroup with SARS-CoV-2 infection aHR(95%CI)[c]/ IRR	Author, year
**Autoimmune disease**	**vaccine type (dose)**	**28-30 day cumulative incidence[a]/365 days[b]**	**after removing SARS-CoV-2 infection**	**Historical (pre-vaccine) cohort [a]/ both periods [b]**	**28-30 day cumulative incidence[a]/365 days**	**after removing SARS-CoV-2 infection**				
ANCA associated vasculitis	overall mRNA (≥ 1)	1.0/10^5	0.013/10^5	up to December 2020[a]	0.9/10^5	0.009/10^5	1.09 (0.72-1.66)[c]	1.24 (0.77-1.99)[c]	0.88 (0.49-1.59)[c]	Jung et al. (2024), Republic of Korea (11)
Anti-phospholipid antibody syndrome	BNT 162b2 (1st)	0.27/10^5 [a]	6.20/10^5	January 2018 to June 2021[b]	0.19/10^5[a]	3.42/10^5	1.42* (NR)	1.18* (NR)	NR	Li et al. (2022), China (13)
	BNT 162b2 (2nd)	0/10^5 [a]	0/10^5	January 2018 to June 2021[b]	0.32/10^5[a]	5.76/10^5	0* (NR)	0* (NR)	NR	Li et al. (2022), China (13)
Behçet disease	overall mRNA (≥ 1)	4.8/10^5	0.038/10^5	up to December 2020 [a]	6.6/10^5	0.066/10^5	0.69 (0.58-0.82)[c]	0.56 (0.45-0.71)[c]	0.84 (0.68-1.03)[c]	Jung et al. (2024), Republic of Korea (11)
Dermato/polymyositis	overall mRNA (≥ 1)	2.2/10^5	0.021/10^5	up to December 2020 [a]	2.0/10^5	0.020/10^5	1.02 (0.77-1.35)[c]	0.92 (0.65-1.29)[c]	1.16 (0.82-1.64)[c]	Jung et al. (2024), Republic of Korea (11)
Psoriatic arthritis	BNT 162b2 (1st)	0.26/10^5 [a]	5.22/10^5	January 2018 to June 2021[b]	0.38/10^5[a]	7.41/10^5	0.68* (NR)	0.70* (NR)	NR	Li et al. (2022), China (13)
	BNT 162b2 (2nd)	0.20/10^5 [a]	3.26/10^5	January 2018 to June 2021[b]	0.37/10^5[a]	7.41/10^5	0.54* (NR)	0.43* (NR)	NR	Li et al. (2022), China (13)
Reactive arthritis	BNT 162b2 (1st)	4.12/10^5 [a]	95.83/10^5	January 2018 to June 2021[b]	8.49/10^5[a]	203.11/10^5	0.48* (NR)	0.47* (NR)	NR	Li et al. (2022), China (13)
	BNT 162b2 (2nd)	7.24/10^5 [a]	193.38/10^5	January 2018 to June 2021[b]	8.56/10^5[a]	210.82/10^5	0.84* (NR)	0.91* (NR)	NR	Li et al. (2022), China (13)
Rheumatoid arthritis	BNT 162b2 (1st)	0.34/10^5 [a]	9.59/10^5	January 2018 to June 2021[b]	1.60/10^5[a]	33.10/10^5	0.21* (NR)	0.28* (NR)	NR	Li et al. (2022), China (13)
	BNT 162b2 (2nd)	0.36/10^5 [a]	4.24/10^5	January 2018 to June 2021[b]	1.69/10^5[a]	31.32/10^5	0.21* (NR)	0.13* (NR)	NR	Li et al. (2022), China (13)
	overall mRNA (≥ 1)	209/10^5	2.06/10^5	up to December 2020 [a]	220/10^5	2.20/10^5	0.86 (0.84-0.89)[c]	0.81 (0.78-0.84)[c]	0.93 (0.89-0.96)[c]	Jung et al. (2024), Republic of Korea (11)
Sjogren's syndrome	BNT 162b2 (1st)	1.14/10^5 [a]	2.91/10^5	January 2018 to June 2021[b]	0.13/10^5[a]	2.28/10^5	8.76* (NR)	1.27* (NR)	NR	Li et al. (2022), China (13)
	BNT 162b2 (2nd)	0/10^5 [a]	0/10^5	January 2018 to June 2021[b]	0.12/10^5[a]	2.47/10^5	0* (NR)	0* (NR)	NR	Li et al. (2022), China (13)
	overall mRNA (≥ 1)	17.4/10^5	0.17/10^5	up to December 2020 [a]	14.5/10^5	0.14/10^5	1.07 (0.96-1.18)[c]	1.04 (0.92-1.17)[c]	1.11 (0.98-1.26)[c]	Jung et al. (2024), Republic of Korea (11)
Spondyloarthritis	BNT 162b2	0.14/10^5 [a]	2.44/10^5	January 2018 to June 2021[b]	0.40/10^5[a]	6.84/10^5	0.35* (NR)	0.35* (NR)	NR	Li et al. (2022), China

(* only for ankylosing spondylitis)	(1st)									(13)
	BNT 162b2 (2nd)	$0/10^5$ [a]	$0/10^5$	January 2018 to June 2021[b]	$0.42/10^5$[a]	$6.59/10^5$	0* (NR)	0* (NR)	NR	Li et al. (2022), China (13)
	overall mRNA (≥ 1) *	$22.6/10^5$	$0.21/10^5$	up to December 2020[a]	$22.4/10^5$	$0.22/10^5$	0.95 (0.87-1.04)[c]	0.87 (0.78-0.97)[c]	1.04 (0.94-1.16)[c]	Jung et al. (2024), Republic of Korea (11)
Systemic lupus erythematosus	BNT 162b2 (1st)	$0.15/10^5$ [a]	$3.89/10^5$	January 2018 to June 2021[b]	$1.21/10^5$[a]	$19.39/10^5$	0.12* (NR)	0.20* (NR)	NR	Li et al. (2022), China (13)
	BNT 162b2 (2nd)	$0.20/10^5$ [a]	$2.62/10^5$	January 2018 to June 2021[b]	$1.21/10^5$[a]	$18.94/10^5$	0.16* (NR)	0.13* (NR)	NR	Li et al. (2022), China (13)
	overall mRNA (≥ 1)	$11.3/10^5$	$0.10/10^5$	up to December 2020[a]	$9.1/10^5$	$0.091/10^5$	1.16 (1.02-1.32)[c]	1.09 (0.93-1.28)[c]	1.23 (1.05-1.44)[c]	Jung et al. (2024), Republic of Korea (11)
Systemic sclerosis	overall mRNA (≥ 1)	$1.8/10^5$	$0.019/10^5$	up to December 2020[a]	$1.6/10^5$	$0.016/10^5$	1.01 (0.73-1.38)[c]	1.06 (0.73-1.53)[c]	0.94 (0.63-1.41)[c]	Jung et al. (2024), Republic of Korea (11)

Overall mRNA refers to both BNT 162b2 (Pfizer) and mRNA-1273 (Moderna) vaccination
Cell color legend indicating incidence in vaccinated compared to unvaccinated: Reduced risk (significant), Increased risk (significant), No association / neutral
*self-calculated IRR values

Table S16. Incidence Risk Following Homologous and Heterologous mRNA Vaccination Relative to Unvaccinated

Autoimmune disease	mRNA vaccine dose	Unvaccinated group from historical (pre-vaccine)[a]/ concurrent [b] cohort / both periods [c]	Heterologous (non-mRNA cross-vaccination) aHR (95%CI)	Homologous (only mRNA cross-vaccination) aHR[c]/HR[d] (95%CI)	Author, year
ANCA- associated vasculitis	≥ 1	up to 21st December 2020[a]	NR	1.46 (0.64-3.31)[c]	Ju et al. (2023), Republic of Korea (10)
	≥ 1	up to 31st December 2020[a]	1.45 (0.85-2.48)	0.91 (0.55-1.51)[c]	Jung et al. (2024), Republic of Korea (11)
Behcet disease	≥ 1	up to 21st December 2020[a]	0.85 (0.35-2.03)	1.08 (0.76-1.53)[c]	Ju et al. (2023), Republic of Korea (10)
	≥ 1	up to 31st December 2020[a]	0.70 (0.54-0.92)	0.69 (0.57-0.84)[c]	Jung et al. (2024), Republic of Korea (11)
Poly/Dermatomyositis	≥ 1	up to 21st December 2020[a]	0.45 (0.10-2.02)	1.35 (0.86-2.11)[c]	Ju et al. (2023), Republic of Korea (10)
	≥ 1	up to 31st December 2020[a]	0.83 (0.55-1.26)	1.11 (0.82-1.52)[c]	Jung et al. (2024), Republic of Korea (11)
Rheumatoid arthritis	≥ 1	up to 21st December 2020[a]	0.61 (0.52-0.72)	0.97 (0.91-1.03)[c]	Ju et al. (2023), Republic of Korea (10)
	≥ 1	up to 31st December 2020[a]	0.80 (0.76-0.83)	0.90 (0.87-0.93)[c]	Jung et al. (2024), Republic of Korea (11)
	≤ 3rd	January 1st, 2020 to December 31st, 2021[c]	1.45 (0.80-2.61)	0.98 (0.65-1.47)[d]	Kim et al. (2024), Seoul- South Korea (12)
Sjogren’s syndrome	≥ 1	up to 21st December 2020[a]	0.40 (0.21-0.77)	0.97 (0.80-1.18)[c]	Ju et al. (2023), Republic of Korea (10)
	≥ 1	up to 31st December 2020[a]	1.02 (0.88-1.18)	1.10 (0.98-1.23)[c]	Jung et al. (2024), Republic of Korea (11)
	≤ 3rd	January 1st, 2020 to December 31st, 2021[c]	1.39 (0.54-3.54)	1.02 (0.54-1.93)[d]	Kim et al. (2024), Seoul- South Korea (12)
Spondyloarthritis (* only for ankylosing spondylitis)	≥ 1*	up to 21st December 2020[a]	0.71 (0.47-1.06)	1.04 (0.89-1.22)[c]	Ju et al. (2023), Republic of Korea (10)
	≥ 1*	up to 31st December 2020[a]	0.85 (0.75-0.97)	1.01 (0.91-1.11)[c]	Jung et al. (2024), Republic of Korea (11)
	≤ 3rd*	January 1st, 2020 to December 31st, 2021[c]	1.56 (0.75-3.26)	0.79 (0.46-1.37)[d]	Kim et al. (2024), Seoul- South Korea (12)
Systemic lupus erythematosus	≥ 1	up to 21st December 2020[a]	0.14 (0.03-0.57)	0.84 (0.64-1.10)[c]	Ju et al. (2023), Republic of Korea (10)
	≥ 1	up to 31st December 2020[a]	0.99 (0.81-1.21)	1.24 (1.08-1.43)[c]	Jung et al. (2024), Republic of Korea (11)
	≤ 3rd	January 1st, 2020 to December 31st, 2021[c]	0.65 (0.18-2.36)	0.62 (0.30-1.29)[d]	Kim et al. (2024), Seoul- South Korea (12)
Systemic sclerosis	≥ 1	up to 21st December 2020[a]	0.67 (0.16-2.87)	0.86 (0.47-1.54)[c]	Ju et al. (2023), Republic of Korea (10)
	≥ 1	up to 31st December 2020[a]	0.88 (0.55-1.41)	1.06 (0.75-1.50)[c]	Jung et al. (2024), Republic of Korea (11)

Each group’s aHR (95% CI) was calculated in reference to the unvaccinated population, serving as the baseline (reference) group.
Overall mRNA refers to both BNT 162b2 (Pfizer) and mRNA-1273 (Moderna) vaccination
Cell color legend indicating incidence in vaccinated compared to unvaccinated: Reduced risk (significant), Increased risk (significant), No association / neutral

Table S17. Booster vaccination

Autoimmune disease	aHR(95%CI) for with vs without booster vaccination (Jung et al., 2024) (11)
ANCA-associated vasculitis	0.71 (0.32–1.58)
Ankylosing spondylitis	1.08 (0.92–1.26)
Behçet disease	0.99 (0.70–1.39)
Dermato/polymyositis	0.89 (0.54–1.47)
Rheumatoid arthritis	1.14 (1.08–1.21)
Systemic lupus erythematosus	0.98 (0.78–1.24)
Systemic sclerosis	1.17 (0.65–2.12)
Sjögren's syndrome	1.14 (0.94–1.38)

Abbreviation: aHR(95%CI): Adjusted Hazard Ratio (95% Confidence Interval)
Booster vaccination was defined as 3rd dose of mRNA vaccine
Cell color legend indicating incidence in vaccinated compared to unvaccinated: Reduced risk (significant), Increased risk (significant), No association / neutral

Table S18. Follow-up restriction window

Autoimmune disease	Incidence vaccinated		Incidence unvaccinated		Reported effect size aHR (95%CI)		Author, year
	28-30 day follow-up	**without restriction**	28-30 day follow-up	**without restriction**	28-30 day follow-up	**without restriction**	
Anti-phospholipid antibody syndrome	$0/10^5$	$4.61/10^5$	$0.32/10^5$	$8.95/10^5$	0 NS	0.51 NS	**Li et al. (2022) China.** (13)
Dermato/polymyositis	NR	NR	NR	NR	0.56 (0.19 - 1.62)	0.43 (0.22 - 0.84)	Bøås et al. (2026), Norway (8)
Psoriatic arthritis	$0.20/10^5$	$1.59/10^5$	$0.37/10^5$	$7.83/10^5$	0.54 NS	0.20 NS	**Li et al. (2022) China.** (13)
Reactive arthritis	$7.24/10^5$	$211.85/10^5$	$8.56/10^5$	$235.62/10^5$	0.84 NS	0.90 NS	**Li et al. (2022) China.** (13)
Rheumatoid arthritis	NR	NR	NR	NR	1.11 (0.90 - 1.37)	1.13 (0.95 - 1.33)	Bøås et al. (2026), Norway (8)
	$0.36/10^5$	$17.96/10^5$	$1.69/10^5$	$27.25/10^5$	0.21 NS	0.65 NS	**Li et al. (2022) China.** (13)
Sjogren's syndrome	NR	NR	NR	NR	1.20 (0.78 - 1.85)	0.92 (0.65 - 1.29)	Bøås et al. (2026), Norway (8)
	$0/10^5$	$3.35/10^5$	$0.12/10^5$	$2.24/10^5$	0 NS	1.49 NS	**Li et al. (2022) China.** (13)
Spondyloarthritis	NR	NR	NR	NR	0.48 (0.23 - 0.99)	0.73 (0.45 - 1.20)	Bøås et al. (2026), Norway (8)
	$0/10^5$	$0/10^5$	$0.42/10^5$	$7.09/10^5$	0 NS	0 NS	**Li et al. (2022) China.** (13)
Systemic lupus erythematosus	NR	NR	NR	NR	1.30 (0.61 - 2.75)	1.15 (0.63 - 2.09)	Bøås et al. (2026), Norway (8)
	$0.20/10^5$	$1.22/10^5$	$1.21/10^5$	$14.92/10^5$	0.16 NS	**0.08 S**	**Li et al. (2022) China.** (13)
Systemic sclerosis	NR	NR	NR	NR	1.06 (0.51 - 2.22)	0.76 (0.42 - 1.40)	Bøås et al. (2026), Norway (8)

Standardized incidence rates up to the end date of data availability among second dose vaccine recipients and non-vaccinated individuals **from istorical and same period cohorts - Li et al. (2022) China.** (13)
Bøås et al. (2026), Norway (8) provided Hazard ratios adjusted for age, sex, health region, municipality size, household size, household crowding, low family income, country of origin, and previous COVID infection.

The authors did not report the effect size and 95% CI, as such we computed them, but from the graphs we could conclude that none of the associations were statistically significant for the risk favor of autoimmue related side effects.

NS-not significant

Section S7. Risk of bias for the included studies

Table S19. Disproportionality analyses (READUS-PV reporting framework)

Item (Criterion)	Purpose / What Should Be Reported (Score 0-2)	Fraenza et al. 2025 (1)	Jarrot et al.2024 (2)	Mettler et al 2021 (3)	Mettler C et al 2022 (4)	Nakao et al. 2023 (5)	Oh et al. 2024 (6)	Xie et al. 2026 (7)
1. Title Information	Title clearly conveys study focus, analysis type, database, and drug/event terms.	2	2	2	2	2	2	2
2. Background	Describe drug(s), event(s), rationale, and why ICSR databases and disproportionality analysis are suitable.	2	2	2	2	2	2	2
3. Objectives	State specific aims and research questions.	2	2	2	2	2	2	2
4. Study Design	Identify study as disproportionality analysis and clear outline of the design	2	2	2	2	2	2	2
5. Data Description, Access, and Pre-processing	Fully describe database(s), extraction, pre-processing, and transformations.	2	1- no explicit extraction method	1-no data pre-processing methods mentioned	1- no explicit extraction method presented	2	1-lacks extraction data	1 - limited preprocessing details
6. Variables Definition	Define population, drugs, events, confounders, modifiers, and grouping criteria.	2	2	2	2	2	2	2
7. Statistical Methods	Describe descriptive, disproportionality, sensitivity, and other biases providing rationale	2	2	2	2	2	2	2
8. Participants	Describe ICSR selection process and characteristics of included reports including a flow diagram	2	1- selection diagram not presented	2	1-selection diagram not presented	2	2	1 - selection diagram not presented
9. Results of Disproportionality Analyses	Present main disproportionality results with CIs and sensitivity analyses if applicable	2	2	2	2	2	2	2
10. Results of Case-by-Case Analysis	Present detailed individual-level results and causality assessment.	N/A	N/A	N/A	N/A	N/A	N/A	N/A
11. Key Results	Summarize main findings and interpret them in context of literature.	2	2	2	2	2	2	2
12. External Validity	Discuss generalizability, clinical implications, and future research-external validity	2	2	1-limited possibility of generalizability	1-limited discussion and generalizibility but letter to editor article	1-geographic context not fully explored	1-limited discussion about generalizability	1-limited possibility of generalizability
13. Limitations	Acknowledge general and specific study limitations.	2	2	2	2	2	2	2

Figure S1. Cohort studies (ROBINS I version 2)

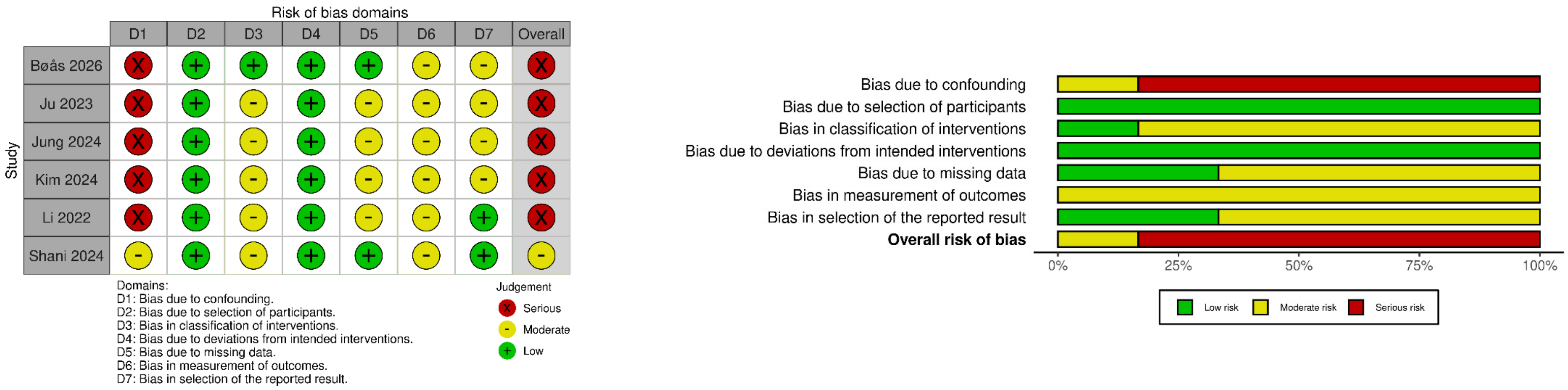

Figure S2. RCTs (RoB 2)

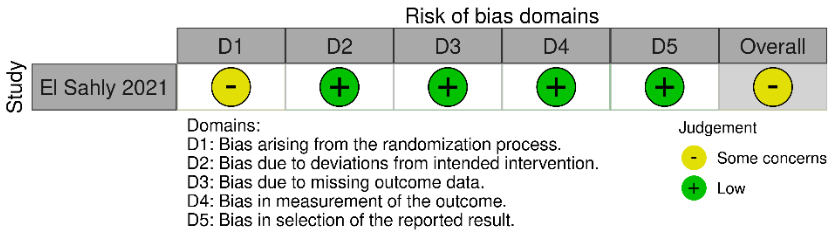

Section S8. Ongoing trials

Table S20. Characteristics of ongoing clinical trials evaluating COVID-19 mRNA vaccines

Trial ID	Study design<br>Phase	Study arms	Participants	Outcomes	Status	Estimated completion date
NCT06255626	Multicenter, RCT<br>Phase 1 and 2	Part 1<br>G1: Low dose CD40.RBDv vaccine non adjuvanted or mRNA vaccine (5:1 ratio)<br>G2: Low dose CD40.RBDv vaccine adjuvanted or mRNA vaccine (5:1 ratio)<br>G3: High dose CD40.RBDv vaccine non adjuvanted or mRNA vaccine (5:1 ratio)<br>G4: SC injection of High dose CD40.RBDv vaccine adjuvanted or mRNA vaccine (5:1 ratio)<br>Part 2<br>G1: Volunteers who received mRNA vaccine in Part 1 will not receive any vaccine<br>G2: Volunteers who received a first dose of CD40.RBDv vaccine (adjuvanted or not) in Part 1 but randomized (1:1) to receive no further dose of vaccine in Part 2.<br>G3: Volunteers who received a first dose of CD40.RBDv vaccine (adjuvanted or not) in Part 1 and randomized (1:1) to receive an additional dose of CD40.RBDv vaccine (adjuvanted or not)	215	The incidence of local/systemic or unsolicited AE, either mild or severe, immunogenicity (seroconversion rate, neutralizing antibodies, local/systemic or unsolicited AE) and efficacy of a Sub-unit Protein CD40.RBDv Bivalent COVID-19 Vaccine	Recruiting	September 2025
NCT05960097	Multicentric, Randomized-controlled study, triple blind<br>Phase 2	Part A<br>G1: CV0701 High dose<br>G2: CV0701 Medium dose<br>G3: CV0701 Low dose<br>G4: CV0601 High dose<br>G5: Control vaccine<br>Part B<br>G1: Baseline-control<br>Participants receive one dose of CV0801<br>G2: Intermediate storage<br>Participants receive one dose of CV0801<br>G3: Maximum storage conditions<br>Participants receive one dose of CV0801	692	The purpose of Part A of this study is to assess the immune response and safety of a booster dose of investigational COVID-19 mRNA vaccines in healthy adults.<br>The purpose of Part B of this study is to assess the immune response and safety of a booster dose of investigational COVID-19 mRNA vaccines in healthy adults.	Completed	August 2024
NCT05911087	Double-blinded , Parallel Controlled	Phase II<br>G1: A single 25 µg dose mRNA vaccine	800	Immunogenicity and safety of COVID-19 mRNA vaccine (SWIM816) compared with mRNA	Unknown status	February 2024

	Trial<br>Phase 2/3	SWIM816<br>G2: A single 25 µg dose mRNA vaccine SW-BIC-213<br>Phase III<br>G1: A single 25 µg dose mRNA vaccine SWIM816<br>G2: A single 30 µg dose mRNA Pfizer Bivalent vaccine		COVID-19 vaccine (Pfizer Bivalent vaccine)		
NCT05686161	Single center, RCT, double blind<br>Phase 3	G1: A single 25 µg dose mRNA vaccine SW-BIC-213<br>G2: A third dose of COVID-19 Inactivated vaccine<br>G3: A single 30µg dose mRNA vaccine Pfizer (BNT162b2)	600	Immunogenicity of mRNA vaccine SW-BIC-213	Unknown status	June 2023
NCT07266558	Randomized, observer-blind, placebo-controlled<br>Phase 4	G1: mRNA-1283 (variant-containing formulation)<br>G2: mRNA-1273 (variant-containing formulation)<br>G3: Placebo	30,000	Efficacy and safety of mRNA-1283 and variant-containing mRNA-1273 boosters<br>Adults aged 50–64 without high-risk conditions<br>Virologically confirmed COVID-19; unsolicited local and systemic adverse events	Active, not recruiting	June 2027
NCT04760132 (ENFORCE)	Open-label, non-randomized, parallel group, with historical controls<br>Phase 4	G1: Comirnaty (BNT162b2, BioNTech-Pfizer)<br>G2: COVID-19 Vaccine Moderna (mRNA-1273)<br>G3: COVID-19 Vaccine AstraZeneca (ChAdOx1 nCoV-19)<br>Vaccination administered as part of the Danish national COVID-19 vaccination programme	7,600	Effectiveness and safety of multiple EU-approved SARS-CoV-2 vaccines offered through the Danish National COVID-19 Vaccination Programme<br>Change from baseline in MPNAT, measured by antibody profiling against SARS-CoV-2 Spike epitopes at 24 months<br>Safety, durability of vaccine-induced immune response, and breakthrough infections	Completed	December 2023

Section S9. List of included articles

1. Fraenza F, Cagnotta C, Gaio M, Sportiello L, Scavone C, Capuano A, et al. Disproportionality analysis of European safety reports on autoimmune and rheumatic diseases following COVID-19 vaccination. Sci Rep [Internet]. 2025 Dec 1 [cited 2025 Dec 14];15(1). Available from: https://pubmed.ncbi.nlm.nih.gov/40289148/

2. Jarrot PA, Mirouse A, Ottaviani S, Cadiou S, Salmon JH, Liozon E, et al. Polymyalgia rheumatica and giant cell arteritis following COVID-19 vaccination: Results from a nationwide survey. Hum Vaccin Immunother [Internet]. 2024 [cited 2025 Dec 14];20(1). Available from: https://pubmed.ncbi.nlm.nih.gov/38563792/

3. Mettler C, Jonville-Bera AP, Grandvuillemin A, Treluyer JM, Terrier B, Chouchana L. Risk of giant cell arteritis and polymyalgia rheumatica following COVID-19 vaccination: a global pharmacovigilance study. Rheumatology (Oxford) [Internet]. 2022 Feb 1 [cited 2025 Dec 14];61(2):865–7. Available from: https://pubmed.ncbi.nlm.nih.gov/34626105/

4. Mettler C, Terrier B, Treluyer JM, Chouchana L. Risk of systemic vasculitis following mRNA COVID-19 vaccination: a pharmacovigilance study. Rheumatology (Oxford) [Internet]. 2022 Dec 1 [cited 2025 Dec 14];61(12):E363–5. Available from: https://pubmed.ncbi.nlm.nih.gov/35640943/

5. Nakao H, Koseki T, Kato K, Yamada S, Tsuboi N, Takahashi K, et al. COVID-19 mRNA vaccination is associated with IgA nephropathy: an analysis of the Japanese adverse drug event report database. J Pharm Pharm Sci [Internet]. 2023 [cited 2025 Dec 14];26:11453. Available from: https://pubmed.ncbi.nlm.nih.gov/37456806/

6. Oh J, Jo H, Park J, Lee H, Kim HJ, Lee H, et al. Global burden of vaccine-associated rheumatic diseases and their related vaccines, 1967–2023: A comprehensive analysis of the international pharmacovigilance database. Int J Rheum Dis [Internet]. 2024 Aug 1 [cited 2025 Dec 14];27(8):e15294. Available from: https://khu.elsevierpure.com/en/publications/global-burden-of-vaccine-associated-rheumatic-diseases-and-their-/

7. Xie R, Cheong LY, Zhang D, et al. Vaccine-associated lupus and related organ involvements: A pharmacovigilance analysis of VAERS database 1990-2025. Seminars in Arthritis and Rheumatism. 2026;77:152933.

8. Bøås H, Lopez-Doriga Ruiz P, Dahl J, Gulseth HL, Tapia G. New-onset autoimmune disease following SARS-CoV-2 infection and mRNA vaccination in Norway: A retrospective cohort study. Journal of Internal Medicine. 2026;299:271-282. Article copyright year: 2025.

9. El Sahly HM, Baden LR, Essink B, Doblecki-Lewis S, Martin JM, Anderson EJ, et al. Efficacy of the mRNA-1273 SARS-CoV-2 Vaccine at Completion of Blinded Phase. New England Journal of Medicine [Internet]. 2021 Nov 4 [cited 2025 Dec 14];385(19):1774–85. Available from: https://www.nejm.org/doi/pdf/10.1056/NEJMoa2113017

10. Ju HJ, Lee JY, Han JH, Lee JH, Bae JM, Lee S. Risk of autoimmune skin and connective tissue disorders after mRNA-based COVID-19 vaccination. J Am Acad Dermatol [Internet]. 2023 Oct 1 [cited 2025 Dec 14];89(4):685–93. Available from: https://pubmed.ncbi.nlm.nih.gov/37187424/

11. Jung SW, Jeon JJ, Kim YH, Choe SJ, Lee S. Long-term risk of autoimmune diseases after mRNA-based SARS-CoV2 vaccination in a Korean, nationwide, population-based cohort study. Nat Commun [Internet]. 2024 Dec 1 [cited 2025 Dec 14];15(1). Available from: https://pubmed.ncbi.nlm.nih.gov/39039113/

12. Kim HJ, Kim MH, Park SJ, Choi MG, Chun EM. Autoimmune adverse event following COVID-19 vaccination in Seoul, South Korea. Journal of Allergy and Clinical Immunology [Internet]. 2024 Jun 1 [cited 2025 Dec 14];153(6):1711–20. Available from: https://pubmed.ncbi.nlm.nih.gov/38520423/

13. Li X, Gao L, Tong X, Chan VKY, Chui CSL, Lai FTT, et al. Autoimmune conditions following mRNA (BNT162b2) and inactivated (CoronaVac) COVID-19 vaccination: A descriptive cohort study among 1.1 million vaccinated people in Hong Kong. J Autoimmun [Internet]. 2022 Jun 1 [cited 2025 Dec 14];130. Available from: https://pubmed.ncbi.nlm.nih.gov/35461018/

14. Shani M, Hermesh I, Feldhamer I, Reges O, Lavie G, Arbel R, et al. The association between BNT162b2 vaccinations and incidence of immune-mediated comorbidities. Vaccine [Internet]. 2024 Jul 11 [cited 2025 Dec 14];42(18):3830–7. Available from: https://pubmed.ncbi.nlm.nih.gov/38729910/

Section S10. List of excluded articles as full text screening with reasons

Ineligible publication type (n = 8)

1.Poh XY, Lee IR, Lim C, Teo J, Rao S, Chia PY, et al. Evaluation of the safety and immunogenicity of different COVID-19 vaccine combinations in healthy individuals: study protocol for a randomized, subject-blinded, controlled phase 3 trial [PRIBIVAC]. Trials. 2022 Jun 16;23(1):498. doi: 10.1186/s13063-022-06345-2. PMID: 35710572; PMCID: PMC9201789.
2. Li, N.L., Coates, P.T., & Rovin, B.H. (2021). COVID-19 vaccination followed by activation of glomerular diseases: Does association equal causation? Kidney International, 100(5), 959–965.
3. Lecler, A., Villeneuve, D., Vignal, C., & Sène, T. (2021). Increased rather than decreased incidence of giant-cell arteritis during the COVID-19 pandemic. Annals of the Rheumatic Diseases, 80(8), e89. https://doi.org/10.1136/annrheumdis-2020-218343.
4. Monti, S., & Montecucco, C. (2021). Response to: 'Increased rather than decreased incidence of giant-cell arteritis during the COVID-19 pandemic' by Lecler et al. Annals of the Rheumatic Diseases, 80(8), e90. https://doi.org/10.1136/annrheumdis-2020-218634.
5. Chinello P, Gavaruzzi F, Epifani AC, Taglietti F. Giant cell arteritis after COVID-19 vaccination/disease: suggestions for further shots? Vascular. 2024;32(2):476-8.
6. Guo M, Ahmed Z, Nguyen P, Zaidi K, Itzkowitz R, Chong E, et al. The impact of COVID-19 mRNA vaccines on immune-related adverse events in patients with cancer receiving immune checkpoint inhibitors. JOURNAL OF CLINICAL ONCOLOGY. 2024;42(16).
7. Klein CR, Heine A, Brossart P, Karakostas P, Schäfer VS. Anti-MDA5 autoantibodies predict clinical dynamics of dermatomyositis following SARS-CoV-2 mRNA vaccination: a retrospective statistical analysis of case reports. Rheumatol Int. 2024;44(10):2185-96.
8. Tyan Y-C, Chuang S-C, Ho T-C, Chuang K-P, Yang M-H. SARS-CoV-2 Vaccine Safety and Autoimmune Response. Vaccines. 2024;12(3).

Ineligible study design (n = 40)

1. Bandinelli F, Pagano M, Vallecoccia MS. Post-COVID-19 and Post-COVID-19 Vaccine Arthritis, Polymyalgia Rheumatica and Horton's Arteritis: A Single-Center Assessment of Clinical, Serological, Genetic, and Ultrasonographic Biomarkers. 2023;12(24).
2. Bettinger JA, Sadarangani M, De Serres G, Valiquette L, Vanderkooi OG, Kellner JD, et al. The Canadian National Vaccine Safety Network: Surveillance of adverse events following immunisation among individuals immunised with the COVID-19 vaccine, a cohort study in Canada. 2022;12(1).
3. Castellon-Lopez Y, Landovitz R, Ntekume E, Porter C, Bross R, Hilder R, et al. A Community Partnered Approach for Diversity in COVID-19 Vaccine Clinical Trials. 2022.
4. Chan ATP, Tang SCW. De novo and relapsing glomerulonephritis after COVID-19 vaccination: how much do we know? 2022;27(1):5-6.
5. Chandler RE. The International Society of Pharmacovigilance Vaccines Special Interest Group: Challenges and Opportunities. 2022;45(6):597-9.
6. Dhanda S, Osborne V, Lynn E, Shakir S. Postmarketing studies: Can they provide a safety net for COVID-19 vaccines in the UK? 2022;27(1):1-6.
7. Finckh A, Ciurea A, Raptis CE, Rubbert-Roth A. Susceptibility to COVID-19 and Immunologic Response to Vaccination in Patients With Immune-Mediated Inflammatory Diseases. J Infect Dis. 2023;228:S13-S23.
8. Heil M. Self-DNA driven inflammation in COVID-19 and after mRNA-based vaccination: lessons for non-COVID-19 pathologies. FRONTIERS IN IMMUNOLOGY. 2024;14.
9. K B, R W, S M, R B, V F, B E, et al. SARS-CoV-2 and allergy - what have we learned after two and a half years? Allergologie select. 2023;7:101-12.
10. Kälkner KM, Sundström A, Nurminen M-L, Larsson M, Ljung R, Arthurson V. Optimizing Safety Surveillance for COVID-19 Vaccines at the Swedish Medical Products Agency. 2023;46(3):319-21.
11. Kaur U, Chakrabarti SS. Need For Focused Pharmacovigilance in the Face of Rapid COVID-19 Vaccine Development. 2023;18(3):273-5.

12. Khehra N, Padda I, Jaferi U, Atwal H, Narain S, Parmar MS. Tozinameran (BNT162b2) Vaccine: The Journey from Preclinical Research to Clinical Trials and Authorization. AAPS PharmSciTech. 2021;22(5).
13. Kountouras J, Papaefthymiou A, Zavos C, Chatzopoulos D, Tzitiridou-Chatzopoulou M, Kavaliotis J, et al. Viewing the Current Puzzling Issue of COVID-19 Vaccination Safety in Older Adults. 2023;24(10):1612-3.
14. Lacroix C, Salvo F, Gras-Champel V, Gautier S, Massy N, Valnet-Rabier M-B, et al. French organization for the pharmacovigilance of COVID-19 vaccines: A major challenge. 2021;76(4):297-303.
15. Mahaux O, Powell G, Haguinet F, Sobczak P, Saini N, Barry A, et al. Identifying Safety Subgroups at Risk: Assessing the Agreement Between Statistical Alerting and Patient Subgroup Risk. 2023;46(6):601-14.
16. Mahroum N, Shoenfeld Y. COVID-19 vaccination can occasionally trigger autoimmune phenomena, probably via inducing age-associated B cells. 2022;25(1):5-6.
17. Manzo C, Castagna A, Nune A, Isetta M. Polymyalgia rheumatica and polymyalgia-like syndromes as adverse events following immunisation with COVID-19 vaccines: a 15 months update. 2023;61(5):408-9.
18. PL M, G C, N F, T H, B Z, P S, et al. Safety monitoring of bivalent mRNA COVID-19 vaccine among pregnant persons in the vaccine adverse event reporting System - United States, September 1, 2022 - March 31, 2023. Vaccine. 2024.
19. Sachinidis A, Garyfallos A. COVID-19 vaccination can occasionally trigger autoimmune phenomena, probably via inducing age-associated B cells. 2022;25(1):83-5.
20. Sprent J, King C. COVID-19 vaccine side effects: The positives about feeling bad. 2021;6(60).
21. Top KA, Chen RT, Levy O, Ozonoff A, Carleton B, Crawford NW, et al. Advancing the Science of Vaccine Safety during the Coronavirus Disease 2019 (COVID-19) Pandemic and Beyond: Launching an International Network of Special Immunization Services. Clin Infect Dis. 2022;75(1):S11-S7.
22. Ursini F, Ruscitti P, Raimondo V, De Angelis R, Cacciapaglia F, Pigatto E, et al. Spectrum of short-term inflammatory musculoskeletal manifestations after COVID-19 vaccine administration: a report of 66 cases. 2022;81(3):440-1.
23. V M-T, V S, J C-P, M M-U, A T, Z dSV, et al. Increased incidence of giant cell arteritis and associated stroke during the COVID-19 pandemic in Spain: A nation-wide population study. Autoimmunity reviews. 2023;22(6):103341.
24. Valnet-Rabier M-B, Tebacher M, Gautier S, Micallef J, Salvo F, Pariente A, et al. Pharmacovigilance signals from active surveillance of mRNA platform vaccines (tozinameran and elasomeran). THERAPIE. 2023;78(5):499-507.
25. Van Laar JA. Can SARS-CoV-2 vaccinations induce auto-inflammatory diseases? 2022;61:SI110-SI1.
26. Wilkinson B, Patel KS, Smith K, Walker R, Wang C, Greene AM, et al. A Brighton Collaboration standardized template with key considerations for a benefit/risk assessment for the Novavax COVID-19 Vaccine (NVX-CoV2373), a recombinant spike protein vaccine with Matrix-M adjuvant to prevent disease caused by SARS-CoV-2 viruses. VACCINE. 2023;41(45):6762-73.
27. Yamamoto K. Adverse effects of COVID-19 vaccines and measures to prevent them. 2022;19(1).
28. Ye X-F. Progress and thinking of signal detection methodology on post-marketing adverse drug reaction surveillance. 2022;43(2):117-22.
29. Yoshimi R, Nakajima H. COVID-19 Vaccination and the Development of Autoimmune Diseases. 2023;62(10):1387-8.
30. Zamora JI, López-Martínez M, Patricio Liebana M, Leon Román JC, Bermejo S, Vergara A, et al. Epidemiology of Immune-Mediated Glomerulopathies before and after SARS-CoV-2 Vaccination: A Tertiary Referral Hospital Experience. J Clin Med. 2023;12(6).
31. Paiva P, Antonio N, Dias P, Carvalho C, Jordao I, Prata I, et al. Acute adverse reactions to COVID-19 Comirnaty mRNA vaccine: a cross-sectional study in healthcare workers from a tertiary care hospital. EUROPEAN JOURNAL OF CLINICAL PHARMACOLOGY. 2022;78:S114.
32. A Phase 1, Open-Label, Comparator-Controlled, Dose-Escalation Study to Evaluate the Safety and Immunogenicity of a Single Dose of VRC H1ssF-3928 mRNA-LNP in Healthy Adults2023 2023.
33. Aoki R, Nihei Y, Matsuzaki K, Suzuki H, Kihara M, Ogawa A, et al. Gross Hematuria after the COVID-19 mRNA Vaccination: Nationwide Multicenter Prospective Cohort Study in Japan. 2024;5(9):1322-32.
34. L W, Z M, L Z, F S. Molecular Pathogenic Mechanisms of IgA Nephropathy Secondary to COVID-19 mRNA Vaccination. Kidney & blood pressure research. 2024;49(1):144-54.
35. Marks P. Defining the Rates of Rare COVID-19 Vaccine–Related Adverse Events. 2024;99(10):1543-4.
36. Pather S, Charpentier N, van den Ouweland F, Rizzi R, Finlayson A, Salisch N, et al. A Brighton Collaboration standardized template with key considerations for a benefit-risk assessment for the Comirnaty COVID-19 mRNA vaccine. Vaccine. 2024;42(22).

37. Á P, D D, M K, K S. Unexpected renal side effects of mRNA COVID-19 vaccines; a single-center experience and short review. The American journal of the medical sciences. 2025.
38. Intapiboon P, Seepathomnarong P, Ongarj J, Surasombatpattana S, Uppanisakorn S, Mahasirimongkol S, et al. Immunogenicity and Safety of an Intradermal BNT162b2 mRNA Vaccine Booster after Two Doses of Inactivated SARS-CoV-2 Vaccine in Healthy Population. VACCINES. 2021;9(12).
39. Laemmle-Ruff I, Fryk JJ, Shenton P, Clothier HJ, Parsons S, Iles L, et al. Detailed review of mortality reported following COVID-19 vaccination in Victoria, Australia: 2021-2023. 2024;42(26).
40. Watad A, De Marco G, Mahajna H, Druyan A, Eltity M, Hijazi N, et al. Immune-Mediated Disease Flares or New-Onset Disease in 27 Subjects Following mRNA/DNA SARS-CoV-2 Vaccination. VACCINES. 2021;9(5).

Ineligible population (n = 36)

1. A G-M, L B-N, R R-R, AS F-OC, M N-P, LM V. Short- and mid-term outcomes in systemic lupus erythematosus patients presenting with disease exacerbation after SARS-CoV-2 mRNA vaccination: A cohort study from Puerto Rico. Lupus. 2023;32(4):571-9.
2. Abhishek A, Peckham N, Pade C, Gibbons JM, Cureton L, Francis A, et al. Effect of a 2-week interruption in methotrexate treatment on COVID-19 vaccine response in people with immune-mediated inflammatory diseases (VROOM study): a randomised, open label, superiority trial. Lancet Rheumat. 2024;6(2):e92-e104.
3. Braverman G, Barbhaiya M, Nong M, Bykerk VP, Hupert N, Lewis C, et al. Association of COVID-19 Vaccinations With Flares of Systemic Rheumatic Disease: A Case-Crossover Study. 2024.
4. CA I, EE S, JL K, PC L, C E, K R, et al. An Argentinean cohort of patients with rheumatic and immune-mediated diseases vaccinated for SARS-CoV-2: the SAR-CoVAC Registry-protocol and preliminary data. Clinical rheumatology. 2022;41(10):3199-209.
5. Carubbi F, Alunno A, Santilli J, Natali L, Mancini B, Di Gregorio N, et al. Immune-mediated inflammatory diseases after anti-SARS-CoV-2 vaccines: new diagnoses and disease flares. 2022;8(2).
6. Chae SH, Park HJ, Radnaabaatar M, Park H, Jung J. Relationship Between Coronavirus Disease 2019 Vaccination Rates and Rare But Potentially Fatal Adverse Events: A Regression Discontinuity Analysis of Western Countries. JOURNAL OF KOREAN MEDICAL SCIENCE. 2023;38(11).
7. Copland E, Hirst J, Mi E, Patone M, Chen D, Coupland C, et al. Effectiveness and safety of COVID-19 vaccination in people with blood cancer. 2024;201.
8. García Ramírez P, Castilla García L, Aspa Cilleruelo JM. Worsening of immune thrombocytopenic purpura in SARS-CoV-2 vaccinated patients. 2022;158(10):497-8.
9. Govere-Hwenje S, Jarolimova J, Yan J, Khumalo A, Zondi G, Ngcobo M, et al. Willingness to accept COVID-19 vaccination among people living with HIV in a high HIV prevalence community. 2022;22(1).
10. Isnardi CA, Cerda OL, Schneeberger EE, Landi M, Calle Montoro C, Alfaro MA, et al. EFFICACY and SAFETY of SARS-COV-2 THIRD VACCINE in PATIENTS with RHEUMATOID ARTHRITIS WHO DID NOT RESPOND after PRIMARY TWO-DOSE REGIMEN. Ann Rheum Dis. 2022;81:1679.
11. Isnardi CA, Roberts K, Quintana R, Kreimer J, Echeverria C, Luna PC, et al. SAFETY and EFFICACY of VACCINES for SARS-COV-2 in PATIENTS with RHEUMATIC and IMMUNEMEDIATED INFLAMMATORY DISEASES: DATA from the ARGENTINEAN REGISTRY SAR-COVAC. Ann Rheum Dis. 2022;81:1665-6.
12. J L, M G, C H, Z I, S R, S L-T, et al. SARS-CoV-2 breakthrough infections among vaccinated individuals with rheumatic disease: results from the COVID-19 Global Rheumatology Alliance provider registry. RMD open. 2022;8(1).
13. JJY L, S B, JC K, Q L, TSH K, J W. Safety and Health Care Use Following COVID-19 Vaccination Among Adults With Rheumatoid Arthritis: A Population-Based Self-Controlled Case Series Analysis. The Journal of rheumatology. 2023.
14. JW K, JY J, CH S, HA K. Flare of adult-onset Still's disease following mRNA COVID-19 vaccination: a case report and review of literature. Clinical rheumatology. 2022;41(5):1583-9.
15. Kharouf F, Eviatar T, Braun M, Pokroy-Shapira E, Brodavka M, Zloof Y, et al. A deep look into the storm: Israeli multi-center experience of coronavirus disease 2019 (COVID-19) in patients with autoimmune inflammatory rheumatic diseases before and after vaccinations. FRONTIERS IN IMMUNOLOGY. 2023;14.
16. Kim S, Seok HY. Evaluation of the safety profile of COVID-19 vaccines in patients with MS, NMOSD, and MOGAD. NEUROLOGICAL SCIENCES. 2023;44(6):1841-8.

17. M W, N S, EV L, AA K, KD J, NN U, et al. COVID-19 Vaccination and New Onset Glomerular Disease: Results from the IRocGN2 International Registry. Kidney360. 2023;4(3):349-62.
18. MT L, W C, SH Y, S P, JY K, DR N, et al. Safety Profiles of mRNA COVID-19 Vaccines Using World Health Organization Global Scale Database (VigiBase): A Latent Class Analysis. Infectious diseases and therapy. 2023;12(2):443-58.
19. P R, S P. Vogt-Koyanagi-Harada disease following ChAdOx1 nCoV-19 and mRNA-1273 vaccination. Journal francais d'ophtalmologie. 2023;46(3):207-10.
20. Peet CJ, Papadopoulou C, Sombrito BRM, Wood MR, Lachmann HJ. COVID-19 and autoinflammatory diseases: prevalence and outcomes of infection and early experience of vaccination in patients on biologics. RHEUMATOLOGY ADVANCES IN PRACTICE. 2021;5(2).
21. Rella V, Busto G, Rotondo C, Fornaro M, Colia R, Corrado A, et al. SAFETY PROFILE of COVID VACCINES in ARTHRITIS PATIENTS. A TWO-CENTERS STUDY. Ann Rheum Dis. 2022;81:1710-1.
22. Rizzi M, Tonello S, Brinno C, Zecca E, Matino E, Cittone M, et al. SARS-CoV-2 infection risk is higher in vaccinated patients with inflammatory autoimmune diseases or liver transplantation treated with mycophenolate due to an impaired antiviral immune response: results of the extended follow up of the RIVALSA prospective cohort. FRONTIERS IN IMMUNOLOGY. 2023;14.
23. Shehab M, Alrashed F, Abdullah I, Alfadhli A, Ali H, Abu-Farha M, et al. Impact of BNT162b2 mRNA Vaccination on the Development of Short and Long-Term Vaccine-Related Adverse Events in Inflammatory Bowel Disease: A Multi-Center Prospective Study. FRONTIERS IN MEDICINE. 2022;9.
24. Simoncelli E, Conticini E, Colafrancesco S, Gattamelata A, Spinelli FR, Garufi C, et al. Multicentre case-control study evaluating the safety of anti-SARS-CoV-2 vaccines in a cohort of patients with systemic vasculitis. CLINICAL AND EXPERIMENTAL RHEUMATOLOGY. 2023;41(4):922-7.
25. Sran HK, Wong E, D'Costa MR, Lim A, Lim Q, Vathsala A. Adverse Renal Events After BNT162b2 Pfizer/BioNTech COVID-19 Vaccine in Kidney Transplant Recipients. Am J Transplant. 2022;22:1066-7.
26. SSY W. Post BNT162b2 mRNA COVID-19 vaccination Henoch-Schönlein Pupura. Postgraduate medical journal. 2022;98(1166):900-1.
27. Stastna D, Menkyova I, Drahota J, Hrnciarova T, Kubala Havrdova E, Vachova M, et al. To be or not to be vaccinated: The risk of MS or NMOSD relapse after COVID-19 vaccination and infection. 2022;65.
28. Stoll S, Desai S, Levit E. A retrospective evaluation of seroconversion after COVID-19 during the early Omicron wave in fully vaccinated multiple sclerosis patients receiving anti-CD20 therapies. 2023;71.
29. Visentini M, Gragnani L, Santini SA, Urraro T, Villa A, Monti M, et al. Flares of mixed cryoglobulinaemia vasculitis after vaccination against SARS-CoV-2. 2022;81(3):441-3.
30. Zulfiqar B, Bittar M, Austin D, Hunter A. COVID Vaccinations in Patients with Rheumatologic Diseasesperspectives and Disparities in a Safety-net Tertiary Care Hospital in Memphis, Tennessee. Arthritis Rheum. 2022;74:178-9.
31. Ahmad A, Baker DL. An unusual side effect of the COVID-19 vaccine: a possible trigger of Polymyalgia Rheumatica. Cureus. 2022;14. doi:10.7759/cureus.26617.
32. Ursini F, Ruscitti P, Addimanda O, Foti R, Raimondo V, Murdaca G, Caira V, Pigatto E, Cuomo G, Lo Gullo A, et al. Inflammatory rheumatic diseases with onset after SARS-CoV-2 infection or COVID-19 vaccination: a report of 267 cases from the COVID-19 and ASD group. RMD Open. 2023;9(2). doi:10.1136/rmdopen-2023-003022.
33. Diebold, M., Locher, E., Boide, P., Enzler-Tschudy, A., Faivre, A., Fischer, I., Helmchen, B., Hopfer, H., Kim, M.J., Moll, S., et al. (2022). Incidence of new onset glomerulonephritis after SARS-CoV-2 mRNA vaccination is not increased. Kidney International, 102(6), 1409–1419.
34. Chen Y, Tucker M, Beckermann K, Iams W, Rini B, Johnson D. COVID-19 mRNA vaccines and immune-related adverse events in cancer patients treated with immune checkpoint inhibitors. EUROPEAN JOURNAL OF CANCER. 2021;155:291-3.
35. Erol Ç, Yalçin T, Sari N, Bayraktar N, Soy E, Çolak M, et al. Differences in Antibody Responses Between an Inactivated SARS-CoV-2 Vaccine and the BNT162b2 mRNA Vaccine in Solid-Organ Transplant Recipients. EXPERIMENTAL AND CLINICAL TRANSPLANTATION. 2021;19(12):1334-40.
36. N G, A T, A H, S D, B Z, NM M, et al. Hybrid versus vaccine immunity of mRNA-1273 among people living with HIV in East and Southern Africa: a prospective cohort analysis from the multicentre CoVPN 3008 (Ubuntu) study. EClinicalMedicine. 2025;80:103054.

Ineligible intervention (n = 63)

1. A J, A S, M K, V B. Mild Adverse Events of Sputnik V Vaccine in Russia: Social Media Content Analysis of Telegram via Deep Learning. Journal of medical Internet research. 2021;23(11):e30529.
2. Al Kaabi N, Yang Y, Eldin Hussein S, Yang T, Abdalla J, Wang H, et al. Efficacy and Safety of a Booster Vaccination with Two Inactivated SARS-CoV-2 Vaccines on Symptomatic COVID-19 Infection in Adults: Results of a Double-Blind, Randomized, Placebo-Controlled, Phase 3 Trial in Abu Dhabi. 2023;11(2).
3. Ali MT, Johnson M, Irwin T, Henry S, Sugeng L, Kansal S, et al. Incidence of Severe Adverse Drug Reactions to Ultrasound Enhancement Agents in a Contemporary Echocardiography Practice. 2024;37(3):276-84.e3.
4. Ba Z, Yang A, Zhu S, Li Y, Ma J, Zhang Y, et al. Comprehensive evaluation of the effect of inactivated SARS-CoV-2 vaccination on female fertility: A retrospective cohort study. 2023;95(10).
5. Babicki M. Will Bivalent Vaccination against COVID-19 Increase the Desire for COVID-19 Vaccination among Poles? 2022;10(10).
6. Bosaeed M, Balkhy HH, Almaziad S, Aljami HA, Alhatmi H, Alanazi H, et al. Safety and immunogenicity of ChAdOx1 MERS vaccine candidate in healthy Middle Eastern adults (MERS002): an open-label, non-randomised, dose-escalation, phase 1b trial. 2022;3(1):e11-e20.
7. Cohen G, Jungsomsri P, Sangwongwanich J, Tawinprai K, Siripongboonsitti T, Porntharukchareon T, et al. Immunogenicity and reactogenicity after heterologous prime-boost vaccination with CoronaVac and ChAdox1 nCov-19 (AZD1222) vaccines. 2022;18(5).
8. Greenberg V, Vazquez-Benitez G, Kharbanda EO, Daley MF, Fu Tseng H, Klein NP, et al. Tdap vaccination during pregnancy and risk of chorioamnionitis and related infant outcomes. 2023;41(22):3429-35.
9. Heath PT, Galiza EP, Baxter DN, Boffito M, Browne D, Burns F, et al. Safety and efficacy of NVX-CoV2373 Covid-19 vaccine. 2021;385(13):1172-83.
10. Heitmann J, Tandler C, Marconato M, Habibzade T, Rittig SM, Tegeler CM, et al. Phase-I/II-Studie eines Peptid-basierten COVID-19-Impfstoffs zur Induktion von T-Zell-Antworten gegen SARS-CoV-2 bei Krebspatienten mit B-Zell-Defizienz. 2022;45:262-3.
11. Herman GA, O'Brien MP, Forleo-Neto E, Sarkar N, Isa F, Hou P, et al. Efficacy and safety of a single dose of casirivimab and imdevimab for the prevention of COVID-19 over an 8-month period: a randomised, double-blind, placebo-controlled trial. 2022;22(10):1444-54.
12. Hobbs FDR, Montgomery H, Padilla F, Simón-Campos JA, Kim K, Arbetter D, et al. Outpatient Treatment with AZD7442 (Tixagevimab/Cilgavimab) Prevented COVID-19 Hospitalizations over 6 Months and Reduced Symptom Progression in the TACKLE Randomized Trial. 2023;12(9):2269-87.
13. Hosain R, Aquino P, Baccarini C, Smolenov I, Li P, Qin H, et al. Six-month safety follow-up of an adjuvanted SARS-CoV-2 trimeric S-protein subunit vaccine (SCB-2019) in adults: A phase 2/3, double-blind, randomized study. 2023;41(13):2253-60.
14. Izumo T, Awano N, Kuse N, Sakamoto K, Takada K, Muto Y, et al. Efficacy and safety of sotrovimab for vaccinated or unvaccinated patients with mild-to-moderate COVID-19 in the omicron era. DRUG DISCOVERIES AND THERAPEUTICS. 2022;16(3):124-7.
15. Januszewski M, Ziuzia-Januszewska L, Jakimiuk AA, Wierzba W, Głuszko A, Żytyńska-Daniluk J, et al. Is the course of covid-19 different during pregnancy? A retrospective comparative study. 2021;18(22).
16. Jose M, Rajmohan P, Thomas J, Krishna S, Antony B, Gopinathan UU, et al. Active Symptom-Based Surveillance of Adverse Events Following Immunization Among Individuals Vaccinated with ChAdOx1 nCoV-19 Corona-virus Vaccine in a Tertiary Hospital of Kerala. 2022;17(4):327-34.
17. JS H, C T, M M, A N, T H, SM R, et al. Phase I/II trial of a peptide-based COVID-19 T-cell activator in patients with B-cell deficiency. Nature communications. 2023;14(1):5032.
18. Karron RA, Key NS, Sharfstein JM. Assessing a Rare and Serious Adverse Event following Administration of the Ad26.COV2.S Vaccine. 2021;325(24):2445-7.
19. Liu Y, Zhai G, Fu W, Zhang X, Xu J. A randomized, double-blind, placebo-controlled phase I trial of inhalation treatment of recombinant TFF2-IFN protein: A multifunctional candidate for the treatment of COVID-19. 2022;13.
20. Marfoh K, Samba A, Okyere E, Acheampong F, Owusu E, Darko DNA, et al. Adverse events following immunisation (AEFI) of COVISHIELD vaccination among healthcare workers in Ghana. 2023;13(6).
21. Micallef B, Dogné J-M, Sultana J, Straus SMJM, Nisticò R, Serracino-Inglott A, et al. An Exploratory Study of the Impact of COVID-19 Vaccine Spontaneous Reporting on Masking Signal Detection in EudraVigilance. 2023;46(11):1089-103.

22. Palassin P, Bres V, Hassan S, Alfonsi A, Massy N, Gras-Champel V, et al. Comprehensive description of adult-onset Still's disease after COVID-19 vaccination. JOURNAL OF AUTOIMMUNITY. 2023;134.
23. Park JY, Choi S-H, Chung J-W, Hwang M-H, Kim M-C. Systemic adverse events and use of antipyretics predict the neutralizing antibody positivity early after the first dose of chadox1 coronavirus disease vaccine. 2021;10(13).
24. Park S, Suh Y-K. A Comprehensive Analysis of COVID-19 Vaccine Discourse by Vaccine Brand on Twitter in Korea: Topic and Sentiment Analysis. 2023;25.
25. Pérez-Rodríguez S, de la Caridad Rodríguez-González M, Ochoa-Azze R, Climent-Ruiz Y, Alberto González-Delgado C, Paredes-Moreno B, et al. A randomized, double-blind phase I clinical trial of two recombinant dimeric RBD COVID-19 vaccine candidates: Safety, reactogenicity and immunogenicity. 2022;40(13):2068-75.
26. Schneider J, Sottmann L, Greinacher A, Hagen M, Kasper H-U, Kuhnen C, et al. Postmortem investigation of fatalities following vaccination with COVID-19 vaccines. 2021;135(6):2335-45.
27. Takalani A, Robinson M, Jonas P, Bodenstein A, Sambo V, Jacobson B, et al. Safety of a second homologous Ad26.COV2.S vaccine among healthcare workers in the phase 3b implementation Sisonke study in South Africa. 2024;42(6):1195-9.
28. Takuva S, Takalani A, Seocharan I, Yende-Zuma N, Reddy T, Engelbrecht I, et al. Safety evaluation of the single-dose Ad26. COV2.S vaccine among healthcare workers in the Sisonke study in South Africa: A phase 3b implementation trial. 2022;19(6).
29. Tayyar R, Wong LK, Dahlen A, Shu E, Pandey S, Liu AY. High-titer post-vaccine COVID-19 convalescent plasma for immunocompromised patients during the first omicron surge. 2023;25(2).
30. Tequare MH, Abraha HE, Adhana MT, Tekle TH, Belayneh EK, Gebresilassie KB, et al. Adverse events of Oxford/AstraZeneca's COVID-19 vaccine among health care workers of Ayder Comprehensive Specialized Hospital, Tigray, Ethiopia. 2021;1:124-9.
31. Toback S, Galiza E, Cosgrove C, Galloway J, Goodman AL, Swift PA, et al. Safety, immunogenicity, and efficacy of a COVID-19 vaccine (NVX-CoV2373) co-administered with seasonal influenza vaccines: an exploratory substudy of a randomised, observer-blinded, placebo-controlled, phase 3 trial. 2022;10(2):167-79.
32. Toledo-Romaní ME, García-Carmenate M, Valenzuela-Silva C, Baldoquín-Rodríguez W, Martínez-Pérez M, Rodríguez-González M, et al. Safety and efficacy of the two doses conjugated protein-based SOBERANA-02 COVID-19 vaccine and of a heterologous three-dose combination with SOBERANA-Plus: a double-blind, randomised, placebo-controlled phase 3 clinical trial. 2023;18.
33. Tukhvatulin AI, Dolzhikova IV, Shcheblyakov DV, Zubkova OV, Dzharullaeva AS, Kovyrshina AV, et al. An open, non-randomised, phase 1/2 trial on the safety, tolerability, and immunogenicity of single-dose vaccine “Sputnik Light” for prevention of coronavirus infection in healthy adults. 2021;11.
34. Valim V, Martins-Filho OA, Gouvea MDPG, Camacho LAB, Villela DAM, de Lima SMB, et al. Effectiveness, safety, and immunogenicity of half dose ChAdOx1 nCoV-19 COVID-19 Vaccine: Viana project. 2022;13.
35. Walach H, Ofner M, Ruof V, Herbig M, Klement RJ. Why do people consent to receiving SARS-CoV-2 vaccinations? A representative survey in Germany. 2022;12(8).
36. Wang JY, Roehrl MW, Roehrl VB, Roehrl MH. A master autoantigen-ome links alternative splicing, female predilection, and COVID-19 to autoimmune diseases. 2022;5.
37. Westrop SJ, Whitaker HJ, Powell AA, Power L, Whillock C, Campbell H, et al. Real-world data on immune responses following heterologous prime-boost COVID-19 vaccination schedule with Pfizer and AstraZeneca vaccines in England. JOURNAL OF INFECTION. 2022;84(5):692-700.
38. Wilson E, Goswami J, Baqui AH, Doreski PA, Perez-Marc G, Zaman K, et al. Efficacy and Safety of an mRNA-Based RSV PreF Vaccine in Older Adults. NEW ENGLAND JOURNAL OF MEDICINE. 2023;389(24):2233-44.
39. Wong LP, Alias H, Danaee M, Ahmed J, Lachyan A, Cai CZ, et al. COVID-19 vaccination intention and vaccine characteristics influencing vaccination acceptance: a global survey of 17 countries. 2021;10(1).
40. Wu Z, Hu Y, Xu M, Chen Z, Yang W, Jiang Z, et al. Safety, tolerability, and immunogenicity of an inactivated SARS-CoV-2 vaccine (CoronaVac) in healthy adults aged 60 years and older: a randomised, double-blind, placebo-controlled, phase 1/2 clinical trial. 2021;21(6):803-12.
41. Zeng G, Wu Q, Pan H, Li M, Yang J, Wang L, et al. Immunogenicity and safety of a third dose of CoronaVac, and immune persistence of a two-dose schedule, in healthy adults: interim results from two single-centre, double-blind, randomised, placebo-controlled phase 2 clinical trials. 2022;22(4):483-95.

42. Zhang R, Chan K-H, Wang P, Zhou R, Yau HK-C, Wong CK-W, et al. A Phase 1, Randomized, Double-Blinded, Placebo-Controlled and Dose-Escalation Study to Evaluate the Safety and Immunogenicity of the Intranasal DelNS1-nCoV-RBD LAIV for COVID-19 in Healthy Adults. 2023;11(4).
43. Zhao Y, Zhao Y, Su X, Zhou Y, Zhang Z, Zhang Y, et al. No association of vaccination with inactivated COVID-19 vaccines before conception with pregnancy complications and adverse birth outcomes: A cohort study of 5457 Chinese pregnant women. 2023;95(4).
44. Zhu F, Jin P, Zhu T, Wang W, Ye H, Pan H, et al. Safety and Immunogenicity of a Recombinant Adenovirus Type-5-Vectored Coronavirus Disease 2019 (COVID-19) Vaccine with a Homologous Prime-Boost Regimen in Healthy Participants Aged ≥6 Years: A Randomized, Double-Blind, Placebo-Controlled, Phase 2b Trial. 2022;75(1):E783-E91.
45. Zhu F, Zhuang C, Chu K, Zhang L, Zhao H, Huang S, et al. Safety and immunogenicity of a live-attenuated influenza virus vector-based intranasal SARS-CoV-2 vaccine in adults: randomised, double-blind, placebo-controlled, phase 1 and 2 trials. 2022;10(8):749-60.
46. Zhu Q, Zhang Y, Kang J, Chen Z, Peng M, Chen M, et al. Weakened humoral and cellular immune response to the inactivated COVID-19 vaccines in Chinese individuals with obesity/overweight. 2023;10(2):608-17.
47. Song Y, Pan S, Tian J, Yu Y, Wang S, Qiu Q, et al. Activation of CD14+ Monocytes via the IFN-γ Signaling Pathway Is Associated with Immune-Related Adverse Events in Hepatocellular Carcinoma Patients Receiving PD-1 Inhibition Combination Therapy. Biomedicines. 2024;12(6).
48. Bonzano L, Djuric O, Mancuso P, Fares L, Brancaccio R, Ottone M, et al. Incidence and Characteristics of Adverse Events after COVID-19 Vaccination in a Population-Based Programme. VACCINES. 2022;10(7).
49. Shan J, Hu X, Chen T, Wang Y, Huang B, Xin Y, et al. COVID-19 vaccination and the risk of autoimmune diseases: a Mendelian randomization study. Frontiers in Public Health. 2024;12.
50. Prontskus V et al., COVID-19 Vaccination and the Incidence of De Novo or Recurrent Rheumatoid Arthritis: A French and International (VigiBase) Signal Detection Study. 2023; 113(5): 1107- 16.

51. An Investigator Initiated, Randomized, Double-blinded, Placebo-controlled Clinical Trial to Evaluate the Safety, Immunogenicity and Efficacy of the Recombinant Two-component COVID-19 Vaccine (CHO Cell) in Adults Aged 18 Years and Older 2023 2023.
52. Interior Mot, Health D. A Phase IV Vaccine Study Under the National Cohort Study of Effectiveness and Safety of SARS-CoV-2 (COVID-19) Vaccines (ENFORCE PLUS)2021 2021-7-1.
53. Kim HW, Kim EH, Roh YH, Joo YS, Eom M, Kim HS, et al. Glomerulonephritis following COVID-19 infection or vaccination: a multicenter study in South Korea. Kidney Res Clin Pract. 2024;43(2):165-76.
54. Västerbotten R, Värmland County Council S, Landsting ÖL, Sormland County Council S, Härjedalen RJ. CoVacc - Immune Response to Vaccination Against Covid-19, an Open Multicenter Phase IV Study2021 2021-5-31.
55. D H, A J, A P, R J, K M, HL C, et al. Safety and immunogenicity of mRNA-based seasonal influenza vaccines formulated to include multiple A/H3N2 strains with or without the B/Yamagata strain in US adults aged 50-75 years: a phase 1/2, open-label, randomised trial. United States2025 2025-1. 25-35 p.
56. Hannawi S, Saifeldin L, Abuquta A, Alamadi A, Mahmoud S, Hassan A, et al. Safety and immunogenicity of a bivalent SARS-CoV-2 protein booster vaccine, SCTV01C, in adults previously vaccinated with mRNA vaccine: a randomized, double-blind, placebo-controlled phase 1/2 clinical trial. EBIOMEDICINE. 2023;87.
57. Li Y, Li J, Dang Y, Chen Y, Tao C. Adverse Events of COVID-19 Vaccines in the United States: Temporal and Spatial Analysis. 2024;10.
58. RJ L, X Q, RN L, JL G, A H, M M, et al. Safety, tolerability, and immunogenicity of PIKA-adjuvanted recombinant SARS-CoV-2 spike protein subunit vaccine in healthy adults: an open-label randomized phase I clinical trial. Clinical and experimental vaccine research. 2024;13(4):315-28.
59. EbolaCov: A Phase IV, Single-centre, Single-blinded, Randomized Controlled Trial to Assess Safety and Immunogenicity of rVSVΔG-ZEBOV-GP Vaccination When Dosed Concurrent With mRNA COVID-19 Vaccine Booster Doses in Healthy African Adults2024 2024.
60. Francisco PS, Centre RB, Sharp M, LLC D. EbolaCov: A Phase IV, Single-centre, Single-blinded, Randomized Controlled Trial to Assess Safety and Immunogenicity of rVSVΔG-ZEBOV-GP Vaccination When Dosed Concurrent With mRNA COVID-19 Vaccine Booster Doses in Healthy African Adults2024 2024-9-4.

61. Ciaffi J, Torrigiani G, Ruscitti P, Zambon A, Ursini F. COVID-19 vaccination and systemic autoimmune rheumatic diseases: No evidence of disproportionately increased reporting in VAERS. Semin Arthritis Rheum. 2026;77:152911.
62. Frydrysiak-Brzozowska A, Haor B, Pluta A, Głowacka M. Population-Based Analysis of Vaccination Status and Post-Vaccination Adverse Events in Adults Aged 55 and Older. Journal of Clinical Medicine. 2025;14(12).
63. Kim JA, Park S, Cho H, Jeong NY, Choi NK. Sex differences in adverse event reporting rates following COVID-19 vaccination in South Korea during the pandemic. International Journal of Infectious Diseases. 2026;162.

Ineligible comparison (n = 16)

1. mRNA Vaccination to Boost Antibodies Against SARS-CoV-2 in Recipients of Inactivated Vaccines (the "mBoost" Study)2021 2021.
2. A Clinical Trial to Assess the Safety and Immunogenicity of LNP-nCOV saRNA-02, a Self-amplifying Ribonucleic Acid (saRNA) Vaccine, in SARS-CoV-2 Seronegative and Seropositive Uganda Population2021 2021.
3. A Single Center, Open Label Clinical Study to Evaluate the Safety and Immunogenicity of SARS-CoV-2 mRNA Vaccine (SYS6006) as Heterologous Booster in Participants Aged 18 Years and Older Vaccinated With Inactivated SARS-CoV-2 Vaccine2022 2022.
4. A Phase 3, Open-label, Randomized, Controlled Study to Evaluate the Immune Response, Safety and Reactogenicity of RSVPreF3 OA Investigational Vaccine When Co-administered With a COVID-19 mRNA Vaccine (Omicron XBB.1.5) in Adults Aged 50 Years and Above2024 2024.
5. AK RS, I W, W D, J C, K J, I E-F, et al. Immunogenicity and Safety of Influenza and COVID-19 Multicomponent Vaccine in Adults ≥50 Years: A Randomized Clinical Trial. United States2025 2025-6-10. 1977-87 p.
6. Indonesia NAIC. Safety Profile Following Moderna COVID-19 Primary Vaccine in Healthy Adults Aged ≥ 18 Years in Indonesia2023 2023-8-22.
7. Vettoretti L, Bouaskeur C, Magy-Bertrand N, Gautier S, Rabier MBV. Relapse and New-Onset of Autoimmune or Inflammatory Diseases Following Vaccination With SPIKEVAX: Pharmacovigilance Overview From the French Spontaneous Reported System. Fundam Clin Pharmacol. 2025 Aug;39(4):e70027. doi: 10.1111/fcp.70027. PMID: 40452215.
8. Shumnalieva R, Ravichandran N, Hannah J, Javaid M, Darooka N, Roy D, Gonzalez DE, Velikova T, Milchert M, Kuwana M, Joshi M, Gracia-Ramos AE, Boyd P, Yaadav P, Cheng K, Kobert L, Cavagna L, Sen P, Day J, Makol A, Gutiérrez CET, Caballero-Uribe CV, Saha S, Parodis I, Dey D, Nikiphorou E, Distler O, Kadam E, Tan AL, Shinjo SK, Ziade N, Knitza J, Chinoy H, Aggarwal R, Agarwal V, Gupta L; COVAD Study Group. Characteristics of emerging new autoimmune diseases after COVID-19 vaccination: A sub-study by the COVAD group. Int J Rheum Dis. 2024 May;27(5):e15178.
9. Alves K, Kotloff K, McClelland RS, Hammershaimb EA, Kouassi A, Plested JS, et al. Phase 2/3 open-label study on NVX-CoV-2601 (XBB.1.5) vaccine in previously COVID-19 mRNA vaccinated and vaccine-naive, SARS-CoV-2–seropositive participants: A 6-month follow-up. Vaccine X. 2025;27.
10. Ito S, Tsuchida N, Kusunoki S, Kaneko Y, Naito T, Hori S, et al. Safety comparison between Pfizer BNT162b2, Moderna mRNA-1273, and AstraZeneca AZD1222 in a Nationwide prospective cohort survey at the beginning of the severe acute respiratory syndrome coronavirus 2 vaccination in Japan. VACCINE. 2025;49.
11. Golstein MA. Post-COVID-19 Vaccine Hyperproduction of Anti-Spike Antibodies and Rheumatological Manifestations. Vaccines (Basel). 2025 Sep 30;13(10):1028. doi: 10.3390/vaccines13101028. PMID: 41150416; PMCID: PMC12567560.
12. Baden LR, Long-term safety and effectiveness of mRNA-1273 vaccine in adults: COVE trial open-label and booster phases. Nat Commun. 2024 Aug 29;15(1):7469

13. Nazar W, Romantowski J, Nazar G, Niedoszytko M, Braun-Dullaeus R, Danilowicz-Szymanowicz L. Serious adverse drug reactions associated with anti-SARS-CoV-2 vaccines and their reporting trends in the EudraVigilance database. Sci Rep. 2025;15(1). (*without effect estimates BNT162b2 and mRNA-1273 head-to-head comparison*)

14. Kim SJ, Rhee TG, Shim SR. Autoimmune and auto-inflammatory adverse events after COVID-19 vaccination in the United States. Clin Immunol. 2024 Feb;259:109882. doi: 10.1016/j.clim.2023.109882. Epub 2023 Dec 27. PMID: 38158168. (*without effect estimates BNT162b2 and mRNA-1273 head-to-head comparison*)
15. Kim S, Bea S, Choe S-A, Choi N-K, Shin J-Y. Autoimmune disorders reported following COVID-19 vaccination: A disproportionality analysis using the WHO database. Eur J Clin Pharmacol. 2024;80(3):445-53. (*without effect estimates BNT162b2 and mRNA-1273 head-to-head comparison*)
16. Shetty, A.N., Mallard, J.H., Hennessy, D. et al. Giant Cell Arteritis Following COVID-19 Vaccination: A Consumer-Stimulated Analysis. Drug Saf 49, 705–715 (2026). https://doi.org/10.1007/s40264-026-01650-0. (*The analytical component used a self-controlled case series design comparing risk and control periods within vaccinated individuals; the pharmacovigilance component used all other vaccines as the disproportionality reference.)*

Ineligible outcome (n = 619)

1. Safety, immunogenicity, and efficacy of the mRNA vaccine CS-2034 as a heterologous booster versus homologous booster with BBIBP-CorV in adults aged ≥18 years: a randomised, double-blind, phase 2b trial. LANCET INFECTIOUS DISEASES. 2023;23(9):1020-30.
2. A B, G U, M M, M B, K A, N S-M, et al. Vaccine Side Effects in Health Care Workers after Vaccination against SARS-CoV-2: Data from TüSeRe:exact Study. Viruses. 2022;15(1).
3. Ab Rahman N, Lim MT, Lee FY, Lee SC, Ramli A, Saharudin SN, et al. Risk of serious adverse events after the BNT162b2, CoronaVac, and ChAdOx1 vaccines in Malaysia: A self-controlled case series study. 2022;40(32):4394-402.
4. Abbas S, Abbas B, Amir S, Wajahat M. Evaluation of adverse effects with covid-19 vaccination in pakistan. 2021;37(7):1-6.
5. Abdel-Qader DH, Abdel-Qader H, Silverthorne J, Kongkaew C, Meslamani AZA, Hayajneh W, et al. Real-World Effectiveness of Four Types of COVID-19 Vaccines. 2023;11(5).
6. Abukhalil AD, Shatat SS, Abushehadeh RR, Al-Shami N, Naseef HA, Rabba A. Side effects of Pfizer/BioNTech (BNT162b2) COVID-19 vaccine reported by the Birzeit University community. 2023;23(1).
7. Agrati C, Castilletti C, Goletti D, Sacchi A, ra, Bordoni V, et al. Persistent Spike-specific T cell immunity despite antibody reduction after 3 months from SARS-CoV-2 BNT162b2-mRNA vaccine. SCIENTIFIC REPORTS. 2022;12(1).
8. Akaishi T, Kushimoto S, Katori Y, Sugawara N, Igarashi K, Fujita M, et al. Effectiveness of mRNA COVID-19 Vaccines in Japan during the Nationwide Pandemic of the Delta Variant. TOHOKU JOURNAL OF EXPERIMENTAL MEDICINE. 2022;257(1):1-6.
9. Akaishi T, Onodera T, Takahashi T, Harigae H, Ishii T. Reports of acute adverse events in mRNA COVID-19 vaccine recipients after the first and second doses in Japan. SCIENTIFIC REPORTS. 2022;12(1).
10. AL A, M D, G S, JT C, M D, TS C. A Multicenter Cohort Study on the Adverse Effects Evaluation After Messenger RNA COVID-19 Vaccination Among Pregnant Healthcare Employees in Penang General Hospitals. Frontiers in public health. 2022;10:876966.
11. AL B, ND P, XE B, JM C, F L, JE O, et al. Analysis of COVID-19 Vaccine Type and Adverse Effects Following Vaccination. JAMA network open. 2021;4(12):e2140364.
12. Al-Sawalmeh K, Pandes M, Niño JA, Avila-Casado C. Acute kidney injury after Pfizer COVID-19 vaccine due to crescentic fibrillary glomerulonephritis. Clin Nephrol. 2022;98(4):205-8.
13. Aldali HJJ, Aldali JAA, Alotaibi BAA, Alasiri GAA, Alromih AMM, Elsokkary EMM, et al. Evaluating the Adverse Events Associated with Three Doses of the COVID-19 Vaccination in Adults in the Western Region of Saudi Arabia: A Cross-Sectional Study. VACCINES. 2023;11(2).
14. Alghamdi AN, Alotaibi I, Mohammed, Alqahtani AS, Al Aboud D, Abdel-Moneim AS. BNT162b2 and ChAdOx1 SARS-CoV-2 Post-vaccination Side-Effects Among Saudi Vaccinees. FRONTIERS IN MEDICINE. 2021;8.
15. Alharbi NK, Al-Tawfiq JA, Alghnam S, Alwehaibe A, Alasmari A, Alsagaby SA, et al. Outcomes of single dose COVID-19 vaccines: Eight month follow-up of a large cohort in Saudi Arabia. 2022;15(5):573-7.

16. Allahgholipour A, Safavi-Naini SAA, Shekarriz Foomany Z, Eskandari A, Nazari Rostami H, Honarvar MJ, et al. COVID-19 vaccines breakthrough infection and adverse reactions in medical students: a nationwide survey in Iran. 2024;12.
17. Almughais ES, Alharbi AH, Aldarwish HA, Alshammari AF, Alsuhaymi RS, Almuaili JA, et al. Side-effects of COVID-19 vaccines among the Saudi population. 2022;43(4):386-93.
18. Alogaili MH, Mohammed LK, Alsaffar AJ, Alsallami AA. Adverse Effects Following the First and Second Dose in Individuals Receiving the Pfizer/BioNTech COVID-19 Vaccine: A Cohort Study. 2022;10(6):642-8.
19. AlOtaibi I, Almuhizi F, Ton-Leclerc S, Fein M, Tsoukas C, Garvey LH, et al. Anaphylaxis induced by mRNA COVID-19 vaccines: follow-up and booster dose after previous desensitization. FRONTIERS IN ALLERGY. 2023;4.
20. AM A, H A, B A, B A, A A, K A, et al. Acute unsolicited adverse events following BNT162b2 vaccine in Saudi Arabia, a real-world data. Vaccine. 2022;40(3):477-82.
21. AM H, J B, P M, TR M, JR S, PG B, et al. Safety Monitoring of COVID-19 Vaccine Booster Doses Among Adults - United States, September 22, 2021-February 6, 2022. MMWR Morbidity and mortality weekly report. 2022;71(7):249-54.
22. AM H, P M, B Z, TR M, J G, JR S, et al. Safety Monitoring of Bivalent COVID-19 mRNA Vaccine Booster Doses Among Persons Aged ≥12 Years - United States, August 31-October 23, 2022. MMWR Morbidity and mortality weekly report. 2022;71(44):1401-6.
23. Amodio E, Minutolo G, Casuccio A, Costantino C, Graziano G, Mazzucco W, et al. Adverse Reactions to Anti-SARS-CoV-2 Vaccine: A Prospective Cohort Study Based on an Active Surveillance System. 2022;10(3).
24. Andersson NW, Thiesson EM, Hansen JV, Hviid A. Safety of BA.4-5 or BA.1 bivalent mRNA booster vaccines: nationwide cohort study. BMJ-BRITISH MEDICAL JOURNAL. 2023;382.
25. Andersson NW, Thiesson EM, Hviid A. Adverse Events After XBB.1.5-Containing COVID-19 mRNA Vaccines. JAMA-JOURNAL OF THE AMERICAN MEDICAL ASSOCIATION. 2024.
26. Andersson NW, Thiesson EM, Laursen MV, Mogensen SH, Kjær J, Hviid A. Safety of heterologous primary and booster schedules with ChAdOx1-S and BNT162b2 or mRNA-1273 vaccines: nationwide cohort study. 2022.
27. Anjorin AA, Odetokun IA, Nyandwi JB, Elnadi H, Awiagah KS, Eyedo J, et al. Public Health Surveillance for Adverse Events Following COVID-19 Vaccination in Africa. 2022;10(4).
28. Ascaso-del-Rio A, García-Pérez J, Pérez-Olmeda M, Arana-Arri E, Vergara I, Pérez-Ingidua C, et al. Immune response and reactogenicity after immunization with two-doses of an experimental COVID-19 vaccine (CVnCOV) followed by a third-fourth shot with a standard mRNA vaccine (BNT162b2): RescueVacs multicenter cohort study. 2022;51.
29. Asefa A, Derjachew N, Belete AM, Talargia F, Melese DM, Getachew B. Adverse reactions following COVID-19 vaccine among healthcare professionals working in Ethiopia: a facility-based cross-sectional study. 2023;11.
30. Ashizawa N, Takazono T, Umeda M, Yamamoto K, Kawakami A, Mukae H. Macrophage activation syndrome after BNT162b2 mRNA vaccination successfully treated with corticosteroids. 2022;40(5):1060.
31. Ashrafian F, Bagheri Amiri F, Bavand A, Zali M, Sadat Larijani M, Ramezani A. A Comparative Study of Immunogenicity, Antibody Persistence, and Safety of Three Different COVID-19 Boosters between Individuals with Comorbidities and the Normal Population. 2023;11(8).
32. Auster O, Finkel U, Dagan N, Barda N, Laufer A, Balicer RD, et al. Short-term Adverse Events After the Third Dose of the BNT162b2 mRNA COVID-19 Vaccine in Adults 60 Years or Older. JAMA NETWORK OPEN. 2022;5(4).
33. Ayoubkhani D, Bermingham C, Pouwels KB, Glickman M, Nafilyan V, Zaccardi F, et al. Trajectory of long covid symptoms after covid-19 vaccination: community based cohort study. BMJ-BRITISH MEDICAL JOURNAL. 2022;377.
34. Azzolini E, Mollura M, Pozzi C, Ubaldi L, Mantovani A, Selmi C, et al. Adverse Events to Comirnaty Vaccine Are Linked to Sex, Age and BMI: Should We Consider Reducing the Dose for Females? 2023;11(3).
35. B C-M, G D, E L, M M. A comparative analysis on serious adverse events reported for COVID-19 vaccines in adolescents and young adults. Frontiers in public health. 2023;11:1145645.
36. BA D, AL M, H G, KE K, JK W, MJ FM, et al. Comparative Safety of BNT162b2 and mRNA-1273 Vaccines in a Nationwide Cohort of US Veterans. JAMA internal medicine. 2022;182(7):739-46.

37. Bardenheier BH, Gravenstein S, Blackman C, Gutman R, Sarkar IN, Feifer RA, et al. Adverse Events Following One Dose of mRNA COVID-19 Vaccination Among US Nursing Home Residents With and Without a Previous SARS-CoV-2 Infection. JOURNAL OF THE AMERICAN MEDICAL DIRECTORS ASSOCIATION. 2021;22(11):2228-32.
38. Bardenheier BH, Gravenstein S, Blackman C, Gutman R, Sarkar IN, Feifer RA, et al. Adverse events following mRNA SARS-CoV-2 vaccination among US nursing home residents. VACCINE. 2021;39(29):3844-51.
39. Bardenheier BH, White EM, Blackman C, Gravenstein S, Gutman R, Sarkar IN, et al. Adverse events following third dose of mRNA COVID-19 vaccination among nursing home residents who received the primary series. JOURNAL OF THE AMERICAN GERIATRICS SOCIETY. 2022;70(6):1642-7.
40. Barzen G, Rieber F, Stangl K, Hahn K, Spethmann S. mRNA vaccines for COVID-19 are safe and clinically effective in patients with cardiac amyloidosis. VACCINE. 2024;42(4):723-6.
41. Beccia F, Regazzi L, Marziali E, Beccia V, Pascucci D, Mores N, et al. BNT162b2 COVID-19 Vaccine Safety among Healthcare Workers of a Tertiary Hospital in Italy. 2023;11(2).
42. Benkebil M, Gautier S, Gras-Champel V, Massy N, Micallef J, Valnet Rabier M-B. COVID-19 vaccines surveillance in France: a global response to a major national challenge. Anaesth Crit Care Pain Med. 2021;40(3).
43. Berar-Yanay N, Freiman S, Shapira M, Saffoury A, Elemy A, Hamze M, et al. Waning humoral response 3 to 6 months after vaccination with the sars-cov-2 bnt162b2 mrna vaccine in dialysis patients. 2022;11(1).
44. Bergman P, Blennow O, Hansson L, Mielke S, Nowak P, Chen P, et al. Safety and efficacy of the mRNA BNT162b2 vaccine against SARS-CoV-2 in five groups of immunocompromised patients and healthy controls in a prospective open-label clinical trial. EBIOMEDICINE. 2021;74.
45. Bookstein Peretz S, Regev N, Novick L, Nachshol M, Goffer E, Ben-David A, et al. Short-term outcome of pregnant women vaccinated with BNT162b2 mRNA COVID-19 vaccine. 2021;58(3):450-6.
46. Borghi MO, Bombaci M, Bodio C, Lonati PA, Gobbini A, Lorenzo M, et al. Anti-Phospholipid Antibodies and Coronavirus Disease 2019: Vaccination Does Not Trigger Early Autoantibody Production in Healthcare Workers. 2022;13.
47. Bots SHH, Riera-Arnau J, Belitser SVV, Messina D, Aragon M, Alsina E, et al. Myocarditis and pericarditis associated with SARS-CoV-2 vaccines: A population-based descriptive cohort and a nested self-controlled risk interval study using electronic health care data from four European countries. FRONTIERS IN PHARMACOLOGY. 2022;13.
48. Bouleftour W, Bonjean P, Grangeon K, Magné N. COVID-19 Vaccine-Related Adverse Events in Solid Cancer Patients Treated with Immunotherapy. 2022;40(9):760-6.
49. Bouwmans P, Messchendorp AL, Sanders JS, Hilbrands L, Reinders MEJ, Vart P, et al. Long-term efficacy and safety of SARS-CoV-2 vaccination in patients with chronic kidney disease, on dialysis or after kidney transplantation: a national prospective observational cohort study. 2022;23(1).
50. Braeye T, Catteau L, Brondeel R, van Loenhout JAF, Proesmans K, Cornelissen L, et al. Vaccine effectiveness against onward transmission of SARS-CoV2-infection by variant of concern and time since vaccination, Belgian contact tracing, 2021. VACCINE. 2022;40(22):3027-37.
51. Burn E, Li X, Kostka K, Stewart HM, Reich C, Seager S, et al. Background rates of five thrombosis with thrombocytopenia syndromes of special interest for COVID-19 vaccine safety surveillance: Incidence between 2017 and 2019 and patient profiles from 38.6 million people in six European countries. 2022;31(5):495-510.
52. Bürzle O, Menges D, Maier JD, Schams D, Puhan MA, Fehr J, et al. Adverse effects, perceptions and attitudes related to BNT162b2, mRNA-1273 or JNJ-78436735 SARS-CoV-2 vaccines: Population-based cohort. 2023;8(1).
53. Bush C, Esposito D, Patel D, Gelman A, Syed S, Dharia P, et al. The impact of data truncation on rates of vaccine-associated adverse events utilizing open and closed claims: Lessons from post-marketing safety surveillance of the SARS-COV-2 MRNA-1273 vaccine. PHARMACOEPIDEMIOLOGY AND DRUG SAFETY. 2022;31(2):79.
54. C A, C M, AP D. COVID-19 Vaccines Adverse Reactions Reported to the Pharmacovigilance Unit of Beira Interior in Portugal. Journal of clinical medicine. 2022;11(19).
55. C R, JN B, Y G, S S, F A, R B, et al. Safety and immunogenicity of booster vaccination and fractional dosing with Ad26.COV2.S or BNT162b2 in Ad26.COV2.S-vaccinated participants. PLOS Global Public Health. 2024;4(4):e0002703.

56. Camacho Moll ME, Salinas Martínez AM, Tovar Cisneros B, García Onofre JI, Navarrete Floriano G, Bermúdez de León M. Extension and Severity of Self-Reported Side Effects of Seven COVID-19 Vaccines in Mexican Population. 2022;10.
57. Caocci G, Fanni D, Porru M, Greco M, Nemolato S, Firinu D, et al. Kikuchi-Fujimoto disease associated with hemophagocytic lymphohistiocytosis following the BNT162b2 mRNA COVID-19 vaccination. 2022;107(5):1222-5.
58. Cari L, Alhosseini MN, Fiore P, Pierno S, Pacor S, Bergamo A, et al. Cardiovascular, neurological, and pulmonary events following vaccination with the BNT162b2, ChAdOx1 nCoV-19, and Ad26.COV2.S vaccines: An analysis of European data. 2021;125.
59. Cari L, Fiore P, Naghavi Alhosseini M, Sava G, Nocentini G. Blood clots and bleeding events following BNT162b2 and ChAdOx1 nCoV-19 vaccine: An analysis of European data. 2021;122.
60. Chan CCH, Choi C-H, Lui WT, Ip B, Ma KKY, Ma SH, et al. A cross-sectional study of COVID-19 vaccination patterns among patients with epilepsy in Hong Kong. EPILEPSIA OPEN. 2022;7(4):570-7.
61. Chauhan MZ, Eleiwa TK, Abdelnaem S, Kwok A, Hunter DG, Phillips PH, et al. Risk of abducens nerve palsy following COVID-19 vaccination. 2024.
62. Chen C, Xie D, Xiao J. Real-world evidence of autoimmune hepatitis following COVID-19 vaccination: A population-based pharmacovigilance analysis. FRONTIERS IN PHARMACOLOGY. 2023;14.
63. Chen G, Li X, Sun M, Zhou Y, Yin M, Zhao B, et al. COVID-19 mRNA Vaccines Are Generally Safe in the Short Term: A Vaccine Vigilance Real-World Study Says. FRONTIERS IN IMMUNOLOGY. 2021;12.
64. Chen G, Ren Q, Zhou J, Zhou Y, Luo H, Wang Y, et al. Association of Current Active Illnesses and Severe Acute Kidney Injury after COVID-19 Vaccines: A Real-World Study. 2022;10(5).
65. Cheng A, Hsieh M-J, Chang S-Y, Ieong S-M, Cheng C-Y, Sheng W-H, et al. Correlation of adverse effects and antibody responses following homologous and heterologous COVID19 prime-boost vaccinations. JOURNAL OF THE FORMOSAN MEDICAL ASSOCIATION. 2023;122(5):384-92.
66. Cheng FWT, Wong CKH, Qin SX, Chui CSL, Lai FTT, Li X, et al. Risk of glomerular diseases, proteinuria and hematuria following mRNA (BNT162b2) and inactivated (CoronaVac) SARS-CoV-2 vaccines. 2023;38(1):129-37.
67. Cheung ECL, Leung MTY, Chen K, Wan EYF, Li X, Lai FTT, et al. Risk of Adverse Events and Delirium after COVID-19 Vaccination in Patients Living with Dementia. 2023;24(6):892-900.e12.
68. Choi A, Koch M, Wu K, Chu L, Ma LZ, Hill A, et al. Safety and immunogenicity of SARS-CoV-2 variant mRNA vaccine boosters in healthy adults: an interim analysis. Nat Med. 2021;27(11):2025-31.
69. Choi YY, Kim M-K, Kwon HC, Kim GH. Safety Monitoring after the BNT162b2 COVID-19 Vaccine among Adults Aged 75 Years or Older. 2021;36(45):1-13.
70. Chow SY, Hsu Y-R, Fong VH. Central retinal artery occlusion after Moderna mRNA-1273 vaccination. 2022;121(11):2369-70.
71. Ciniselli CM, Lecchi M, Figini M, Melani CC, Daidone MG, Morelli D, et al. COVID-19 Vaccination in Health Care Workers in Italy: A Literature Review and a Report from a Comprehensive Cancer Center. 2022;10(5).
72. CKH W, KTK L, X X, ICH A, FTT L, EYF W, et al. Adverse events of special interest and mortality following vaccination with mRNA (BNT162b2) and inactivated (CoronaVac) SARS-CoV-2 vaccines in Hong Kong: A retrospective study. PLoS medicine. 2022;19(6):e1004018.
73. Claaß LV, Mayr P, Paschold L, Weber T, Terziev D, Jehs B, et al. No association of malignant B-cell non-Hodgkin lymphomas with ipsilateral SARS-CoV-2 vaccination. 2023;12(8):9313-21.
74. Clothier HJ, Shetty AN, Mesfin Y, Mackie M, Pearce C, Buttery JP. What would have happened anyway? Population data source considerations when estimating background incident rates of adverse events following immunisation to inform vaccine safety. 2024;42(5):1108-15.
75. Cohen DA, Greenberg P, Formanowski B, Parikh PD. Are COVID-19 mRNA vaccine side effects severe enough to cause missed work? Cross-sectional study of health care-associated workers. MEDICINE. 2022;101(7).
76. Corrao G, Rea F, Franchi M, Cereda D, Barone A, Borriello CR, et al. Balancing Benefits and Harms of COVID-19 Vaccines: Lessons from the Ongoing Mass Vaccination Campaign in Lombardy, Italy. VACCINES. 2022;10(4).
77. Cuschieri S, Borg M, Agius S, Souness J, Brincat A, Grech V. Adverse reactions to Pfizer-BioNTech vaccination of healthcare workers at Malta's state hospital. 2021;75(10).

78. D G-E, C P-P, A P-G, J E-G, M R-M, MM R-A. Self-reported adverse events within the seven days following the Spikevax® (Moderna) vaccination. Farmacia hospitalaria : organo oficial de expresion cientifica de la Sociedad Espanola de Farmacia Hospitalaria. 2022;46(5):301-7.
79. D M. Frequency and Associations of Adverse Reactions of COVID-19 Vaccines Reported to Pharmacovigilance Systems in the European Union and the United States. Frontiers in public health. 2021;9:756633.
80. D M. Uncommon thrombotic complications after SARS-CoV-2 vaccination. Vnitrni lekarstvi. 2021;67(5):297-302.
81. D Y, HL J, Y N, YJ C, SA C, J J, et al. A Nationwide Survey of mRNA COVID-19 Vaccinee's Experiences on Adverse Events and Its Associated Factors. Journal of Korean medical science. 2023;38(22):e170.
82. d'Arminio Monforte A, Tavelli A, Perrone PM, Za A, Razzini K, Tomasoni D, et al. Association between previous infection with SARS CoV-2 and the risk of self-reported symptoms after mRNA BNT162b2 vaccination: Data from 3,078 health care workers. 2021;36.
83. DA H, KN H, AR Z, V M, P C, Y D, et al. Comparative Risks of Potential Adverse Events Following COVID-19 mRNA Vaccination Among Older US Adults. JAMA network open. 2023;6(8):e2326852.
84. Dar-Odeh N, Abu-Hammad O, Qasem F, Jambi S, Alhodhodi A, Othman A, et al. Long-term adverse events of three COVID-19 vaccines as reported by vaccinated physicians and dentists, a study from Jordan and Saudi Arabia. 2022;18(1).
85. de Zalan CS-KA, Bouwman M, van Osch F, Damoiseaux J, van der Snoek M-A, Lunel FV, et al. Changes in Local and Systemic Adverse Effects following Primary and Booster Immunisation against COVID-19 in an Observational Cohort of Dutch Healthcare Workers Vaccinated with BNT162b2 (Comirnaty®). VACCINES. 2024;12(1).
86. Demirbakan H, Kocer I, Berk I, Bayram A. Evaluation of Side Effects After Heterologous Vaccination with BNT162b2 Vaccine in Healthcare Workers Vaccinated with Two Doses of Inactive COVID-19 Vaccine. KLIMIK JOURNAL. 2022;35(4):238-43.
87. Deng L, Glover C, Dymock M, Pillsbury A, Marsh JA, Quinn HE, et al. The short term safety of COVID-19 vaccines in Australia: AusVaxSafety active surveillance, February – August 2021. 2022;217(4):195-202.
88. DeSilva MB, Haapala J, Vazquez-Benitez G, Boyce TG, Fuller C, C. a, et al. Medically Attended Acute Adverse Events in Pregnant People After Coronavirus Disease 2019 (COVID-19) Booster Vaccination. OBSTETRICS AND GYNECOLOGY. 2023;142(1):125-9.
89. di Mauro G, Mascolo A, Longo M, Maiorino MI, Scappaticcio L, Bellastella G, et al. European Safety Analysis of mRNA and Viral Vector COVID-19 Vaccines on Glucose Metabolism Events. PHARMACEUTICALS. 2022;15(6).
90. Di Noia V, Pimpinelli F, Renna D, Barberi V, Maccallini MT, Gariazzo L, et al. Immunogenicity and Safety of COVID-19 Vaccine BNT162b2 for Patients with Solid Cancer: A Large Cohort Prospective Study from a Single Institution. 2021;27(24):6815-23.
91. Di Resta C, Ferrari D, Viganò M, Moro M, Sabetta E, Minerva M, et al. The gender impact assessment among healthcare workers in the sars-cov-2 vaccination—an analysis of serological response and side effects. 2021;9(5).
92. Donze C, Min V, Ninove L, de Lamballerie X, Revon Rivière G, Verschuur A, et al. BNT162b2 COVID-19 Vaccines in Children, Adolescents and Young Adults with Cancer—A 1-Year Follow-Up. 2023;11(5).
93. Dorajoo SR, Tan HX, Teo CHD, Neo JW, Koon YL, Ng JJA, et al. Nationwide safety surveillance of COVID-19 mRNA vaccines following primary series and first booster vaccination in Singapore. 2023;15.
94. DP C, YH W, WT L, FP H. Association between the side effect induced by COVID-19 vaccines and the immune regulatory gene polymorphism. Frontiers in immunology. 2022;13:941497.
95. Dracz B, Mueller V, Takacs I, Hagymasi K, Dinya E, Miheller P, et al. Effectiveness of COVID-19 Vaccination with mRNA Vaccines for Patients with Cirrhosis in Hungary: Multicentre Matched Cohort Study. VACCINES. 2023;11(1).
96. Duijster JW, Lieber T, Pacelli S, Van Balveren L, Ruijs LS, Raethke M, et al. Sex-disaggregated outcomes of adverse events after COVID-19 vaccination: A Dutch cohort study and review of the literature. 2023;14.

97. Dziedzic A, Riad A, Attia S, Klugar M, Tanasiewicz M. Self-Reported Adverse Events of COVID-19 Vaccines in Polish Healthcare Workers and Medical Students. Cross-Sectional Study and Pooled Analysis of CoVaST Project Results in Central Europe. JOURNAL OF CLINICAL MEDICINE. 2021;10(22).
98. Eick-Cost AA, Ying S, Wells N. Effectiveness of mRNA-1273, BNT162b2, and JNJ-78436735 COVID-19 Vaccines Among US Military Personnel Before and During the Predominance of the Delta Variant. JAMA NETWORK OPEN. 2022;5(4).
99. F N, D G, A F, A V, JR GB, E M, et al. Immune-related adverse events and disease outcomes after the third dose of SARS-CoV-2 mRNA-BNT162b2 vaccine in cancer patients receiving immune checkpoint inhibitors. Cancer immunology, immunotherapy : CII. 2023;72(10):3217-28.
100. F S-S, S P, L C, H V, R L, D S, et al. Side effects during the week after first dose vaccination with four Covid-19 vaccines. Results of the ProVaVac Survey Study with 13,837 people in Spain. Vaccine. 2022;40(41):5942-9.
101. Faksova K, Walsh D, Jiang Y, Griffin J, Phillips A, Gentile A, et al. COVID-19 vaccines and adverse events of special interest: A multinational Global Vaccine Data Network (GVDN) cohort study of 99 million vaccinated individuals. Vaccine. 2024;42(9):2200-2211.
102. Fang Y, Li J-X, Duangdany D, Li Y, Guo X-L, Phamisith C, et al. Safety, immunogenicity, and efficacy of a modified COVID-19 mRNA vaccine, SW-BIC-213, in healthy people aged 18 years and above: a phase 3 double-blinded, randomized, parallel controlled clinical trial in Lao PDR (Laos). ECLINICALMEDICINE. 2024;67.
103. Fattizzo B, Roeth A, Broome CM, Khan U, Shafer F, Cordoba M, et al. Concomitant Use of Sutimlimab and COVID-19 Vaccines in Patients with Cold Agglutinin Disease from the Phase 3 Cardinal and Cadenza Studies. Blood. 2022;140:2839-41.
104. Fekete M, Horvath A, Santa B, Tomisa G, Szollosi G, Varga JT. First booster dose uptake of COVID-19 vaccine and diseaserelated factors in chronic obstructive pulmonary disease—a cross-sectional survey in Hungary. 2023;12(3):516-28.
105. Ferawati K, Liew K, Aramaki E, Wakamiya S. Monitoring Mentions of COVID-19 Vaccine Side Effects on Japanese and Indonesian Twitter: Infodemiological Study. JMIR INFODEMIOLOGY. 2022;2(2).
106. Fern, ez-Varela AM, Granero-Lopez M, Rodriguez-Rodriguez A, ro, Rodriguez-Penin I. COVID-19 vaccines: safety profile after one year of follow-up. ARS PHARMACEUTICA. 2024;65(1):6-12.
107. Ferrara F, Mancaniello C, Varriale A, Sorrentino S, Zovi A, Nava E, et al. COVID-19 mRNA Vaccines: A Retrospective Observational Pharmacovigilance Study. CLINICAL DRUG INVESTIGATION. 2022;42(12):1065-74.
108. Ferrara P, Ponticelli D, Losa L, Romeo C, Magliuolo R, Vitale A, et al. Risk of Repeated Adverse Effects following Booster Dose of mRNA COVID-19 Vaccine: Results from the MOSAICO Study. VACCINES. 2023;11(2).
109. Filippatos F, Tatsi E-B, Dellis C, Dessypris N, Syriopoulou V, Michos A. Association of clinical and epidemiological characteristics with COVID-19 BNT162b2 mRNA vaccine short-term adverse reactions in healthcare workers. HUMAN VACCINES \& IMMUNOTHERAPEUTICS. 2021;17(12):4755-60.
110. Flacco ME, Soldato G, Martellucci CA, Carota R, Di Luzio R, Caponetti A, et al. Interim Estimates of COVID-19 Vaccine Effectiveness in a Mass Vaccination Setting: Data from an Italian Province. VACCINES. 2021;9(6).
111. Florea A, Wu J, Qian L, Lewin B, Sy LS, Lin I-C, et al. Risk of herpes zoster following mRNA COVID-19 vaccine administration. EXPERT REVIEW OF VACCINES. 2023;22(1):643-9.
112. Fraiman J, Erviti J, Jones M, Greenl, S, er, et al. Serious adverse events of special interest following mRNA COVID-19 vaccination in randomized trials in adults. VACCINE. 2022;40(40):5798-805.
113. Fujomoto D, Kuwabara T, Mizumoto T, Izumi Y, Adachi M, Miyasato Y, et al. WCN24-721 Clinicopathological features of tubulointerstitial nephritis (TIN) associated with irAE, COVID-19 infection, and COVID-19 vaccination in comparison with classical allergic TIN. Kidney Intl Rep. 2024;9(4):S63.
114. Fusco F, Scognamiglio G, Roma AS, Abbate M, Papaccioli G, Merola A, et al. Mid-term follow-up after COVID-19 vaccination in adults with CHD: a prospective study. CARDIOLOGY IN THE YOUNG. 2023;33(12):2574-80.
115. G SL, N M, M B, G B, D M. Real-life safety profile of mRNA vaccines for COVID-19: An analysis of VAERS database. Vaccine. 2023;41(18):2879-86.
116. Galanis P, Katsiroumpa A, Vraka I, Chrysagi V, Siskou O, Konstantakopoulou O, et al. Prevalence and risk factors of adverse effects after the first COVID-19 booster dose: evidence from Greece. 2023;24(3):210-7.

117. Gallo K, Goede A, Mura C, Abel R, Moahamed B, Preissner S, et al. A Comparative Analysis of COVID-19 Vaccines Based on over 580,000 Cases from the Vaccination Adverse Event Reporting System. 2022;10(3).
118. Gan L-L, Razak ZBA, Teh NHMH, Kamaluzaman NHB, Zulkeplee NAB, Amin INBM, et al. Adverse events following BNT162b2 mRNA COVID-19 vaccination among healthcare workers: A single-centre experience in Malaysia. 2022;77(3):300-5.
119. Garcia M, Albizua-Madariaga I, Lertxundi U, Aguirre C. Subacute thyroiditis and COVID-19 vaccines: a case/non-case study. ENDOCRINE. 2022;77(3):480-5.
120. Garcia-Blanco L, Celorio-Murillo W, Delgado V. Leukocytoclastic vasculitis related to mRNA vaccination against COVID-19. 2022;37:S18-S20.
121. Gazit S, Saciuk Y, Perez G, Peretz A, Pitzer VE, Patalon T. Short term, relative effectiveness of four doses versus three doses of BNT162b2 vaccine in people aged 60 years and older in Israel: retrospective, test negative, case-control study. BMJ-BRITISH MEDICAL JOURNAL. 2022;377.
122. Gazit S, Shlezinger R, Perez G, Lotan R, Peretz A, Ben-Tov A, et al. Severe Acute Respiratory Syndrome Coronavirus 2 (SARS-CoV-2) Naturally Acquired Immunity versus Vaccine-induced Immunity, Reinfections versus Breakthrough Infections: A Retrospective Cohort Study. CLINICAL INFECTIOUS DISEASES. 2022;75(1):E545-E51.
123. Getzler IE, Vered S, Gavze RP, Adler L, Oved M. Unraveling the debate: Can the skin manifestations of SARS-CoV-2 infection and vaccination be explained by the reactivation of herpes virus? JOURNAL OF DERMATOLOGY. 2023;50(12):1594-602.
124. GL C, XF L, XH D, N L, ML C, Z H, et al. Safety and immunogenicity of the SARS-CoV-2 ARCoV mRNA vaccine in Chinese adults: a randomised, double-blind, placebo-controlled, phase 1 trial. The Lancet Microbe. 2022;3(3):e193-e202.
125. Göschl L, Mrak D, Grabmeier-Pfistershammer K, Stiasny K, Haslacher H, Schneider L, et al. Reactogenicity and immunogenicity of the second COVID-19 vaccination in patients with inborn errors of immunity or mannan-binding lectin deficiency. 2022;13.
126. GP V, A L, M M, P N, A B, V G, et al. Safety surveillance after BNT162b2 mRNA COVID-19 vaccination: results from a cross-sectional survey among staff of a large Italian teaching hospital. Acta bio-medica : Atenei Parmensis. 2021;92:e2021450.
127. Gram MA, Nielsen J, Schelde AB, Nielsen KF, Moustsen-Helms IR, Sorensen AKB, et al. Vaccine effectiveness against SARS-CoV-2 infection, hospitalization, and death when combining a first dose ChAdOx1 vaccine with a subsequent mRNA vaccine in Denmark: A nationwide population-based cohort study. PLOS MEDICINE. 2021;18(12).
128. Green MS, Peer V, Magid A, Hagani N, Anis E, Nitzan D. Gender Differences in Adverse Events Following the Pfizer-BioNTech COVID-19 Vaccine. 2022;10(2).
129. Gümüs HH, Ödemis I, Aliska HE, Karsli A, Kara S, Özkale M, et al. Side effects and antibody response of an inactive severe acute respiratory syndrome coronavirus 2 vaccine among health care workers. 2021;67(12):1825-31.
130. H M, Y B, J R, K T, R F, M M, et al. Effect of BNT162b2 mRNA booster vaccination on VO(2) (max) in recreational athletes: A prospective cohort study. Health science reports. 2022;5(6):e929.
131. Ha J, Song MC, Park S, Kang H, Kyung T, Kim N, et al. Deciphering deaths associated with severe serious adverse events following SARS-CoV-2 vaccination: A retrospective cohort study. 2024;16.
132. Halder A, Imamura H, Condon S, Boroughs K, Nilsson SC, Anderson T, et al. Pfizer/BioNtech BNT162b2: adverse events and insights from an Australian mass vaccination clinic for COVID-19. INTERNAL MEDICINE JOURNAL. 2022;52(1):121-4.
133. Han C, Zhao ZZ, Chan P, Li F, Chi CL, Zhang X, et al. A large survey on COVID-19 vaccination in patients with Parkinson's disease and healthy population. 2023;41(43):6483-94.
134. Hanson KE, Goddard K, Lewis N, Fireman B, Myers TR, Bakshi N, et al. Incidence of Guillain-Barre Syndrome After COVID-19 Vaccination in the Vaccine Safety Datalink. JAMA NETWORK OPEN. 2022;5(4).
135. Hara M, Furue T, Fukuoka M, Iwanaga K, Matsuishi E, Miike T, et al. Real-World Effectiveness of the mRNA COVID-19 Vaccines in Japan: A Case-Control Study. VACCINES. 2022;10(5).
136. Harris D, Zullo AR, Mor V, Hayes KN. Comparative safety of BNT162b2 and mRNA-1273 vaccines across frailty subgroups among 6.4 million older adults. J Am Geriatr Soc. 2023;71:S127-S8.
137. Hatmal MM, Al-Hatamleh MAI, Olaimat AN, Hatmal M, Alhaj-Qasem DM, Olaimat TM, et al. Side effects and perceptions following covid-19 vaccination in jordan: A randomized, cross-sectional study implementing machine learning for predicting severity of side effects. Vaccines. 2021;9(6).

138. Hatmal MM, Al-Hatamleh MAI, Olaimat AN, Mohamud R, Fawaz M, Kateeb ET, et al. Reported Adverse Effects and Attitudes among Arab Populations Following COVID-19 Vaccination: A Large-Scale Multinational Study Implementing Machine Learning Tools in Predicting Post-Vaccination Adverse Effects Based on Predisposing Factors. 2022;10(3).
139. Hause AM, Baggs J, Marquez P, Abara WE, Baumblatt JG, Thompson D, et al. Safety Monitoring of COVID-19 mRNA Vaccine First Booster Doses Among Persons Aged ≥12 Years with Presumed Immunocompromise Status - United States, January 12, 2022-March 28, 2022. MMWR Morb Mortal Wkly Rep. 2022;71(28):899-903.
140. Hayes KN, Harris DA, Zullo AR, Djibo DA, Smith-Ray RL, Taitel MS, et al. Data resource profile: COVid VAXines effects on the aged (COVVAXAGE). 2023;8(6).
141. Hensley KS, Jongkees MJ, Geers D, GeurtsvanKessel CH, Mueller YM, Dalm VASH, et al. Immunogenicity and reactogenicity of SARSCoV-2 vaccines in people living with HIV in the Netherlands: A nationwide prospective cohort study. 2022;19(10).
142. HG R, J G, R L, PL M, B Z, P S, et al. Safety of mRNA vaccines administered during the initial 6 months of the US COVID-19 vaccination programme: an observational study of reports to the Vaccine Adverse Event Reporting System and v-safe. The Lancet Infectious diseases. 2022;22(6):802-12.
143. Hidayat R, Mustika AP, Avisha F, Djuliannisaa Z, Winari DD, Putri RA, et al. Surveillance of Adverse Events Following Immunization (AEFI) after Third Dose Booster Vaccination with mRNA-Based Vaccine in Universitas Indonesia Hospital Health Personnel. VACCINES. 2022;10(6).
144. HS M, A M, S L, AA S, BV M, J M, et al. Analyses of reported severe adverse events after immunization with SARS-CoV-2 vaccines in the United States: One year on. Frontiers in public health. 2022;10:972464.
145. Hung PV, Nguyen TD, Ha LT, Toi PL, Tram TH. Common Adverse Events from Mixing COVID-19 Vaccine Booster in Hanoi, Vietnam. 2023;11(6).
146. Hurst M, Jordan A, Capstick J, Brewerton M, Cockroft R, Ingram J. Patients with previous immediate hypersensitivity reactions to polyethylene glycol can safely receive the BNT162b2 mRNA COVID-19 vaccine. INTERNAL MEDICINE JOURNAL. 2022;52(10):1818-20.
147. I S, JH O, Y N, CV W, S B, M B, et al. Expectations and Prior Experiences Associated With Adverse Effects of COVID-19 Vaccination. JAMA network open. 2023;6(3):e234732.
148. Ieven T, Coorevits L, Vandebotermet M, Tuyls S, Vanneste H, Santy L, et al. Endotyping of IgE-Mediated Polyethylene Glycol and/or Polysorbate 80 Allergy. 2023;11(10):3146-60.
149. Ieven T, ebotermet M, Nuyttens L, Devolder D, enberghe P, Bullens D, et al. COVID-19 Vaccination Safety and Tolerability in Patients Allegedly at High Risk for Immediate Hypersensitivity Reactions. VACCINES. 2022;10(2).
150. Im JH, Kim E, Lee E, Seo Y, Lee Y, Jang Y, et al. Adverse Events with the Pfizer-BioNTech COVID-19 Vaccine among Korean Healthcare Workers. YONSEI MEDICAL JOURNAL. 2021;62(12):1162-8.
151. IR S, J M. The person-time ratio distribution for the exact monitoring of adverse events: Historical vs surveillance Poisson data. Statistics in medicine. 2023;42(18):3283-301.
152. Israel A, Schaffer A, A. r, Merzon E, Green I, Magen E, et al. A Calculator for COVID-19 Severity Prediction Based on Patient Risk Factors and Number of Vaccines Received. MICROORGANISMS. 2022;10(6).
153. Ivanko I, Celap I, Margetic S, Marijancevic D, Josipovic J, Gacina P. Changes in haemostasis and inflammatory markers after mRNA BNT162b2 and vector Ad26.CoV2.S SARS-CoV-2 vaccination. THROMBOSIS RESEARCH. 2023;228:137-44.
154. JA B, MA I, HP S, L V, MP M, OG V, et al. Adverse Events Following Immunization With mRNA and Viral Vector Vaccines in Individuals With Previous Severe Acute Respiratory Syndrome Coronavirus 2 Infection From the Canadian National Vaccine Safety Network. Clinical infectious diseases : an official publication of the Infectious Diseases Society of America. 2023;76(6):1088-102.
155. Jain AG, Dong NC, Ball S, Tan ES, Whiting J, Komrokji RS, et al. Responses to sars-cov-2 vaccines in patients with myelodysplastic syndrome and acute myeloid leukemia. Blood. 2021;138:217.
156. Jamshidi E, Asgary A, Kharrazi AY, Tavakoli N, Zali A, Mehrazi M, et al. Personalized predictions of adverse side effects of the COVID-19 vaccines. HELIYON. 2023;9(1).
157. Janekrongtham C, Salazar M, Doung-ngern P. Sex Differences in Serious Adverse Events Reported Following Booster Doses of COVID-19 Vaccination in Thailand: A Countrywide Nested Unmatched Case-Control Study. VACCINES. 2023;11(12).

158. Janssen C, Cachanado M, Ninove L, Lachatre M, Michon J, Epaulard O, et al. Immunogenicity and reactogenicity of heterologous and homologous mRNA-1273 and BNT162b2 vaccination: A multicenter non-inferiority randomized trial. ECLINICALMEDICINE. 2022;48.
159. Janzic U, Bidovec-Stojkovic U, Mohorcic K, Mrak L, Dovnik NF, Ivanovic M, et al. Solid cancer patients achieve adequate immunogenicity and low rate of severe adverse events after SARS-CoV-2 vaccination. FUTURE ONCOLOGY. 2022;18(23):2537-50.
160. Jara LJ, Vera-Lastra O, Mahroum N, Pineda C, Shoenfeld Y. Autoimmune post-COVID vaccine syndromes: does the spectrum of autoimmune/inflammatory syndrome expand? 2022;41(5):1603-9.
161. Jeong HS, Chun BC. Signal detection of COVID-19 vaccines adverse events using spontaneous reports from South Korea. 2023;32(9):961-8.
162. Jimenez Ortega A, Francisco r, de Luna Aguilar A, Benitez Fuentes JD, Navarro PF, Munoz EB, et al. Adverse drug reactions of the SARS-CoV-2 mRNA-1273 vaccine in a cohort of cancer patients of a tertiary hospital in Madrid, Spain. JOURNAL OF CLINICAL ONCOLOGY. 2022;40(16).
163. Jin Z, Wu J, Wang Y, Huang T, Zhao K, Liu J, et al. Safety and immunogenicity of the COVID-19 mRNA vaccine CS-2034: A randomized, double-blind, dose-exploration, placebo-controlled multicenter Phase I clinical trial in healthy Chinese adults. JOURNAL OF INFECTION. 2023;87(6):556-70.
164. Joyce MCC, Mountjoy NJJ, Johnson JAA, Newman JTT, y DLL, Atalla NAA, et al. From trial to practice: incidence and severity of COVID-19 vaccine side effects in a medically at-risk and vaccine-hesitant community. BMC PUBLIC HEALTH. 2022;22(1).
165. JY C, Y L, NG P, MS K, SJ R. Serious Safety Signals and Prediction Features Following COVID-19 mRNA Vaccines Using the Vaccine Adverse Event Reporting System. Pharmaceuticals (Basel, Switzerland). 2024;17(3).
166. K B, G H-S, CD C. Maternal and Child Outcomes Reported by Breastfeeding Women Following Messenger RNA COVID-19 Vaccination. Breastfeeding medicine : the official journal of the Academy of Breastfeeding Medicine. 2021;16(9):697-701.
167. Kadali RAK, Janagama R, Peruru S, Gajula V, Madathala RR, Chennaiahgari N, et al. Non-life-threatening adverse effects with COVID-19 mRNA-1273 vaccine: A randomized, cross-sectional study on healthcare workers with detailed self-reported symptoms. JOURNAL OF MEDICAL VIROLOGY. 2021;93(7):4420-9.
168. Kadali RAK, Janagama R, Peruru S, Malayala V, Srikrishna. Side effects of BNT162b2 mRNA COVID-19 vaccine: A randomized, cross-sectional study with detailed self-reported symptoms from healthcare workers. INTERNATIONAL JOURNAL OF INFECTIOUS DISEASES. 2021;106:376-81.
169. Kant A, Jansen J, van Balveren L, van Hunsel F. Description of Frequencies of Reported Adverse Events Following Immunization Among Four Different COVID-19 Vaccine Brands. 2022;45(4):319-31.
170. Karaali R, Öykü Dinç HÖ, İnanç Balkan İ, Can G, Keskin E, Çolak H, et al. Homologous or heterologous COVID-19 vaccine schemes: comparison of immune responses and side effects. 2023;107(2).
171. Khorraminejad-Shirazi M, Nabavizadeh SS, Aminnia S, Ahmadifar M, Borazjani R, Sadeghi E, et al. Association of Demographic, Clinical, and Vaccination Characteristics with COVID-19 Viral Load Assessed by qRT-PCR. ARCHIVES OF IRANIAN MEDICINE. 2023;26(12):688-94.
172. Kim CY, Mcneill EN, Young C, King F, Clague M, Caldwell M, et al. Observational Study of Patients Hospitalized With Neurologic Events After SARS-CoV-2 Vaccination, December 2020-June 2021. NEUROLOGY-CLINICAL PRACTICE. 2023;13(4).
173. Kim MS, Jung SY, Ahn JG, Park SJ, Shoenfeld Y, Kronbichler A, et al. Comparative safety of mRNA COVID-19 vaccines to influenza vaccines: A pharmacovigilance analysis using WHO international database. JOURNAL OF MEDICAL VIROLOGY. 2022;94(3):1085-95.
174. Kim S, Kang M, Park J-S, Seok HY. Risk and characteristics of Bell's palsy in adults as an adverse event following COVID-19 vaccination: a retrospective study. ACTA NEUROLOGICA BELGICA. 2023;123(6):2185-93.
175. KJ A-A, NE BJ, E ML, M B-B, LG CC. Biventricular Takotsubo Cardiomyopathy as an Unusual Presentation of SARS-CoV-2 mRNA Vaccine-Associated Multisystemic Inflammatory Syndrome. Cureus. 2023;15(7):e41365.
176. Klein NP, Lewis N, Goddard K, Fireman B, Zerbo O, Hanson KE, et al. Surveillance for Adverse Events After COVID-19 mRNA Vaccination. JAMA-JOURNAL OF THE AMERICAN MEDICAL ASSOCIATION. 2021;326(14):1390-9.

177. Komine-Aizawa S, Haruyama Y, Deguchi M, Hayakawa S, Kawana K, Kobashi G, et al. The vaccination status and adverse effects of COVID-19 vaccine among pregnant women in Japan in 2021. JOURNAL OF OBSTETRICS AND GYNAECOLOGY RESEARCH. 2022;48(7):1561-9.
178. Kramer KJ, Wilfong EM, Voss K, Barone SM, Shiakolas AR, Raju N, et al. Single-cell profiling of the antigen-specific response to BNT162b2 SARS-CoV-2 RNA vaccine. NATURE COMMUNICATIONS. 2022;13(1).
179. Kremsner PG, Ahuad Guerrero RA, Arana-Arri E, Aroca Martinez GJ, Bonten M, Ch, et al. Efficacy and safety of the CVnCoV SARS-CoV-2 mRNA vaccine candidate in ten countries in Europe and Latin America (HERALD): a randomised, observer-blinded, placebo-controlled, phase 2b/3 trial. LANCET INFECTIOUS DISEASES. 2022;22(3):329-40.
180. Kubota K, Saginoya T, Ishiwata K, Nakasato T, Munechika H. [18F]FDG uptake in axillary lymph nodes and deltoid muscle after COVID-19 mRNA vaccination: a cohort study to determine incidence and contributing factors using a multivariate analysis. 2022;36(4):340-50.
181. Kuo Y-M, Kang C-M, Lai Z-Y, Huang T-Y, Tzeng S-J, Hsu C-C, et al. Temporal changes in biomarkers of neutrophil extracellular traps and NET-promoting autoantibodies following adenovirus-vectored, mRNA, and recombinant protein COVID-19 vaccination. J Med Virol. 2024;96(3).
182. Chu L, McPhee R, Huang W, Bennett H, Pajon R, Nestorova B, et al. A preliminary report of a randomized controlled phase 2 trial of the safety and immunogenicity of mRNA-1273 SARS-CoV-2 vaccine. VACCINE. 2021;39(20):2791-9.
183. L W, N Z, T K, P S, K Ü, I K, et al. Reactogenicity after heterologous and homologous COVID-19 prime-boost vaccination regimens: descriptive interim results of a comparative observational cohort study. BMC infectious diseases. 2022;22(1):504.
184. Larson V, Seidenberg R, Caplan A, Brinster NK, Meehan SA, Kim R, et al. Clinical and histopathological spectrum of delayed adverse cutaneous reactions following COVID-19 vaccination. JOURNAL OF CUTANEOUS PATHOLOGY. 2022;49(1):34-41.
185. Lazarus R, Baos S, Cappel-Porter H, Carson-Stevens A, Clout M, Culliford L, et al. Safety and immunogenicity of concomitant administration of COVID-19 vaccines (ChAdOx1 or BNT162b2) with seasonal influenza vaccines in adults in the UK (ComFluCOV): a multicentre, randomised, controlled, phase 4 trial. 2021;398(10318):2277-87.
186. Lee DS, Kim JW, Lee KL, Jung YJ, Kang HW. Adverse events following COVID-19 vaccination in South Korea between February 28 and August 21, 2021: A nationwide observational study. INTERNATIONAL JOURNAL OF INFECTIOUS DISEASES. 2022;118:173-82.
187. Lee DY, Kang DY, Kim E, Lee S-j, Baek JH, Lee J-S, et al. Adverse events of a third dose of BNT162b2 mRNA COVID-19 vaccine among Korean healthcare workers. MEDICINE. 2023;102(11).
188. Lee MT, You S-H, Lee JW, Park s, Kim J-Y, Nam DR, et al. Distinguishing patterns of serious adverse events with mRNA COVID-19 vaccines using VigiBase: A latent class analysis. PHARMACOEPIDEMIOLOGY AND DRUG SAFETY. 2022;31(2):626.
189. Lehmann HC, Oberle D, Keller-Stanislawski B, Rieck T, Streit R. Rare cases of Guillain-Barre syndrome after COVID-19 vaccination, Germany, December 2020 to August 2021. EUROSURVEILLANCE. 2023;28(24).
190. Lepelley M, Revol B, Perez J, Vial T, Roustit M, Cracowski JL, et al. Recurrence of adverse events of special interest after COVID-19 vaccines rechallenge: A Vigibase® study. Fundam Clin Pharmacol. 2022;36:39.
191. Leung NHL, Cheng SMS, Martin-Sanchez M, Au NYM, Ng YY, Luk LLH, et al. Immunogenicity of a Third Dose of BNT162b2 to Ancestral Severe Acute Respiratory Syndrome Coronavirus 2 and the Omicron Variant in Adults Who Received 2 Doses of Inactivated Vaccine. CLINICAL INFECTIOUS DISEASES. 2023;76(3):E299-E307.
192. Li J, Sutton K, Ong L, Flower O, Gatward JJ, Jang H, et al. Recent SARS-CoV-2 infection: too early to vaccinate? 2022;216(9):449-51.
193. Li X, Ostropolets A, Makadia R, Shoaibi A, Rao G, Sena AG, et al. Characterising the background incidence rates of adverse events of special interest for covid-19 vaccines in eight countries: Multinational network cohort study. 2021;373.
194. Lim S, Liew E, Leo A, Ng BT, Lee J. Haematological changes and adverse events associated with BNT162b2 mRNA COVID-19 vaccine in patients receiving clozapine-Findings from an audit. ACTA PSYCHIATRICA SCANDINAVICA. 2022;146(2):179-81.

195. Lim SM, Chan HC, Santosa A, Quek SC, Liu EHC, Somani J. Safety and side effect profile of Pfizer-BioNTech COVID-19 vaccination among healthcare workers: A tertiary hospital experience in Singapore. ANNALS ACADEMY OF MEDICINE SINGAPORE. 2021;50(9):703-11.
196. Lim XR, Tan JWL, Chan GYL, Hou J, Xie L, Goh VHL, et al. Evaluation of Patients with Vaccine Allergies Prior to mRNA-Based COVID-19 Vaccination. VACCINES. 2022;10(7).
197. Liu C, Lee J, Ta C, Soroush A, Rogers JR, Kim JH, et al. Risk Factors Associated With SARS-CoV-2 Breakthrough Infections in Fully mRNA-Vaccinated Individuals: Retrospective Analysis. JMIR PUBLIC HEALTH AND SURVEILLANCE. 2022;8(5).
198. Liu X, Shaw RH, Stuart ASV, Greenl, M, Aley PK, et al. Safety and immunogenicity of heterologous versus homologous prime-boost schedules with an adenoviral vectored and mRNA COVID-19 vaccine (Com-COV): a single-blind, randomised, non-inferiority trial. LANCET. 2021;398(10303):856-69.
199. Lloyd PC, Hu M, Wong H-L, Shoaibi A, Zhou CK, Lo A-C, et al. Near real-time surveillance of safety outcomes in US COVID-19 vaccine recipients aged 12 to 64 years. VACCINE. 2022;40(45):6481-8.
200. Loosen SH, Bohlken J, Weber K, Konrad M, Luedde T, Roderburg C, et al. Factors Associated with Non-Severe Adverse Reactions after Vaccination against SARS-CoV-2: A Cohort Study of 908,869 Outpatient Vaccinations in Germany. VACCINES. 2022;10(4).
201. Low JM, Afroze S, Al Mamun MA, Afroze S, Tisha S, Hossain MA, et al. Short term Outcomes of COVID-19 Vaccines Among Lactating Mother and Child Dyads in Bangladesh: A Multi-centre, Cross-sectional Study. MATERNAL AND CHILD HEALTH JOURNAL. 2024.
202. Low JM, Lee LY, Ng YPM, Zhong Y, Amin Z. Breastfeeding Mother and Child Clinical Outcomes After COVID-19 Vaccination. JOURNAL OF HUMAN LACTATION. 2022;38(1):37-42.
203. M H, S M, Y G, T Y, Y K, K I. Adverse reactions to mRNA coronavirus disease 2019 (COVID-19) vaccine for severe acute respiratory syndrome coronavirus 2 (SARS-CoV-2) in 576 medical staff. Fujita medical journal. 2022;8(3):79-82.
204. M L, N U, S B-Z, B W, WM B, E D, et al. Temporal association between COVID-19 vaccination and Raynaud's phenomenon: A case series. Human vaccines & immunotherapeutics. 2023;19(1):2199653.
205. M Y, M M, A P, T P, S G, E S, et al. Safety of the fourth COVID-19 BNT162b2 mRNA (second booster) vaccine: a prospective and retrospective cohort study. The Lancet Respiratory medicine. 2023;11(2):139-50.
206. Macy E, ya S, Sheikh J, Burnette A, Shi JM, Chung J, et al. Population-Based Incidence, Severity, and Risk Factors Associated with Treated Acute-Onset COVID-19 mRNA Vaccination-Associated Hypersensitivity Reactions. JOURNAL OF ALLERGY AND CLINICAL IMMUNOLOGY-IN PRACTICE. 2022;10(3):827-36.
207. Magen O, Waxman JG, Makov-Assif M, Vered R, Dicker D, Hernan MA, et al. Fourth Dose of BNT162b2 mRNA Covid-19 Vaccine in a Nationwide Setting. NEW ENGLAND JOURNAL OF MEDICINE. 2022;386(17):1603-14.
208. Mahmoud NM, Eldegla HE, Shenouda RN, Elsaeed AS, Mahmoud MM, Alemam D, et al. COVID-19 Vaccination Adverse Reactions among Vaccinated Persons in Mansoura University: A Prospective Study. 2024;33(1):37-48.
209. Makanut S, Wangteeraprasert A, Jitpewngam W, Ngoenkam J, Pongcharoen S. Immunological responses and adverse reactions of the heterologous second booster dose of BNT162b2 after two-dose CoronaVac for COVID-19 vaccination in healthcare workers of Faculty of Medicine, Naresuan University. VACCINE. 2023;41(29):4335-40.
210. Markewitz R, Pauli D, Dargvainiene J, Steinhagen K, Engel S, Herbst V, et al. The temporal course of T- and B-cell responses to vaccination with BNT162b2 and mRNA-1273. CLINICAL MICROBIOLOGY AND INFECTION. 2022;28(5):701-9.
211. Martellucci CA, Flacco ME, Soldato G, Di Martino G, Carota R, Caponetti A, et al. Effectiveness of COVID-19 Vaccines in the General Population of an Italian Region before and during the Omicron Wave. VACCINES. 2022;10(5).
212. Massa F, Cremoni M, Gerard A, re, Grabsi H, Rogier L, et al. Safety and Cross-Variant Immunogenicity of a Three-dose COVID-19 mRNA Vaccine Regimen in Kidney Transplant Recipients. EBIOMEDICINE. 2021;73.
213. Massari M, Alegiani SS, Morciano C, Spuri M, Marchione P, Felicetti P, et al. Postmarketing active surveillance of myocarditis and pericarditis following vaccination with COVID-19 mRNA vaccines in persons aged 12 to 39 years in Italy: A multi-database, self-controlled case series study. 2022;19(7).
214. Massy N, Atzenhoffer M, Boulay C, Pecquet P-E, Ledys F, Cracowski J-L, et al. COVID-19 and adenovirus vaccines: French experience of enhanced pharmacovigilance. THERAPIE. 2023;78(5):489-98.

215. Masuda T, Murakami K, Sugiura K, Sakui S, Philip Schuring R, Mori M. A phase 1/2 randomised placebo-controlled study of the COVID-19 vaccine mRNA-1273 in healthy Japanese adults: An interim report. Vaccine. 2022;40(13):2044-52.
216. Mateus Rodriguez J, Bifano M, Mendez Placencia C, Olivé Torralba A, Fontseca Martin E, Roy Millan P. Effect and Tolerability of a Nutritional Supplement in Volunteers Receiving the Influenza or the COVID-19 Vaccine: Randomized, Double-Blind, Placebo-Controlled Study. Eur J Clin Pharmacol. 2022;78:S60.
217. Mathew M, Sebastian J, Basavaraja CK, Josh D, Varughese D, Soman S, et al. A sentinel surveillance of adverse events and breakthrough infections following COVID-19 precautionary dose among south Indian healthcare workers. 2023;7(2).
218. Mathioudakis A, G. e, Ghrew M, Ustianowski A, Ahmad S, Borrow R, et al. Self-Reported Real-World Safety and Reactogenicity of COVID-19 Vaccines: A Vaccine Recipient Survey. LIFE-BASEL. 2021;11(3).
219. Matsuzaki K, Aoki R, Nihei Y, Suzuki H, Kihara M, Yokoo T, et al. Gross hematuria after SARS-CoV-2 vaccination: questionnaire survey in Japan. 2022;26(4):316-22.
220. Mcelvaney OJ, Cleary B, Fraughen DD, Kelly G, Mcelvaney OF, Murphy MP, et al. Safety and Reactogenicity of COVID-19 Vaccination in Severe Alpha-1 Antitrypsin Deficiency. CHRONIC OBSTRUCTIVE PULMONARY DISEASES-JOURNAL OF THE COPD FOUNDATION. 2024;11(1):3-12.
221. McMurry R, Lenehan P, Awasthi S, Silvert E, Puranik A, Pawlowski C, et al. Real-time analysis of a mass vaccination effort confirms the safety of FDA-authorized mRNA COVID-19 vaccines. MED. 2021;2(8):965+.
222. Meeraus W, Stuurman AL, Durukal I, Conde-Sousa E, Lee A, Santa Maria A, et al. COVID-19 vaccine booster doses provide increased protection against COVID-19 hospitalization compared with previously vaccinated individuals: Interim findings from the REFORCO-Brazil real-world effectiveness study during Delta and Omicron. VACCINE. 2023;41(42):6366-78.
223. Mendez-Lizarraga CA, Chacon-Cruz E, Carrillo-Meza R, Hern, ez-Milan NS, Inustroza-Sanchez LC, et al. Report of Adverse Effects Following Population-Wide COVID-19 Vaccination: A Comparative Study between Six Different Vaccines in Baja-California, Mexico. VACCINES. 2022;10(8).
224. Mesina FZ, Sapinoso FAD, De Castro JAV, Vaswani PPM, Sy CES, Oquendo RAA, et al. Hematologic adverse events reported after COVID-19 vaccination in the Philippines: A national database study. 2023;41(23):3550-5.
225. Methi F, Gran JM, Valberg M, Kinge JM, Telle K, Magnusson K. Third dose mRNA vaccination against SARS-CoV-2 reduces medical complaints seen in primary care: a matched cohort study. BMC MEDICINE. 2023;21(1).
226. Milano E, Ricciardi A, Casciaro R, Pallara E, De Vita E, Bavaro DF, et al. Immunogenicity and safety of the BNT162b2 COVID-19 mRNA vaccine in PLWH: A monocentric study in Bari, Italy. JOURNAL OF MEDICAL VIROLOGY. 2022;94(5):2230-6.
227. Mittal A, Solera JT, Ferreira VH, Kothari S, Kimura M, Pasic I, et al. Immunogenicity and Safety of Booster SARS-CoV-2 mRNA Vaccine Dose in Allogeneic Hematopoietic Stem Cell Transplantation Recipients. TRANSPLANTATION AND CELLULAR THERAPY. 2023;29(11).
228. MJM N, C P, JC OH, DW C, E S, G D, et al. Surveillance of Safety of 3 Doses of COVID-19 mRNA Vaccination Using Electronic Health Records. JAMA network open. 2022;5(4):e227038.
229. Modenese A, Paduano S, Bargellini A, Bellucci R, Marchetti S, Bruno F, et al. Neutralizing anti-SARS-CoV-2 antibody titer and reported adverse effects, in a sample of italian nursing home personnel after two doses of the BNT162b2 vaccine administered four weeks apart. 2021;9(6).
230. Mofaz M, Yechezkel M, Guan G, Br, eau ML, Patalon T, et al. Self Reported and Physiologic Reactions to Third BNT162b2 mRNA COVID-19 (Booster) Vaccine Dose. EMERGING INFECTIOUS DISEASES. 2022;28(7):1375-83.
231. Moghaddas F, Tsiougkos N, Grammatikos A, Bright PD, Johnston S, Gompels M. COVID-19 vaccine allergy advice and guidance: The experience of a UK tertiary referral centre. 2023;16(1).
232. Mohr-Sasson A, Haas J, Abuhasira S, Sivan M, Doitch Amdurski H, Dadon T, et al. The effect of Covid-19 mRNA vaccine on serum anti-Müllerian hormone levels. 2022;37(3):534-41.
233. Montalti M, Guaraldi F, Di Valerio Z, Ragghianti B, Tedesco D, Mannucci E, et al. Adherence to and early adverse events of COVID-19 vaccine in a cohort of 600 Italian breastfeeding and pregnant physicians. HUMAN VACCINES \& IMMUNOTHERAPEUTICS. 2022;18(6).
234. Moradi Hasan-Abad A, Arbabi M, Gilasi H, Motedayyen H. Immunogenicity and adverse events of the COVID-19 vaccines in healthy and individuals with autoimmune diseases in an Iranian population. 2024;38.

235. Moreira ED, Kitchin N, Xu X, Dychter SS, Lockhart S, Gurtman A, et al. Safety and Efficacy of a Third Dose of BNT162b2 Covid-19 Vaccine. 2022;386(20):1910-21.
236. Morimoto H, Hayano S, Ozawa N, Ogura Y, Usui H, Usami T, et al. Questionnaire survey of possible association of allergic diseases with adverse reactions to sars-cov-2 vaccination. 2021;9(12).
237. Moro PL, Ennulat C, Brown H, Woody G, Zhang B, Marquez P, et al. Safety of Simultaneous Administration of Bivalent mRNA COVID-19 and Influenza Vaccines in the Vaccine Adverse Event Reporting System (VAERS). DRUG SAFETY. 2024.
238. Moro PL, Olson CK, Clark E, Marquez P, Strid P, Ellington S, et al. Post-authorization surveillance of adverse events following COVID-19 vaccines in pregnant persons in the vaccine adverse event reporting system (VAERS), December 2020-October 2021. VACCINE. 2022;40(24):3389-94.
239. Moro PL, Olson CK, Zhang B, Marquez P, Strid P. Safety of Booster Doses of Coronavirus Disease 2019 (COVID-19) Vaccine in Pregnancy in the Vaccine Adverse Event Reporting System. OBSTETRICS AND GYNECOLOGY. 2022;140(3):421-7.
240. Moscara L, Venerito V, Martinelli A, Di Lorenzo A, Toro F, Violante F, et al. Safety profile and SARS-CoV-2 breakthrough infections among HCWs receiving anti-SARS-CoV-2 and influenza vaccines simultaneously: an Italian observational study. VACCINE. 2023;41(38):5655-61.
241. Moukafih B, Belaroussi L, Achour S, Kartouti AE. Side Effects Reported by Moroccan Medical Students Who Received COVID-19 Vaccines. 2024;19(2):268-76.
242. MT L-S, S DC, M C, S M, R M, A M, et al. mRNA-COVID19 Vaccination Can Be Considered Safe and Tolerable for Frail Patients. Frontiers in oncology. 2022;12:855723.
243. Mungmunpuntipantip R, Wiwanitkit V. Post COVID-19 vaccination and adverse events: Correspondence. 2022;38(4):141.
244. Munro APS, Feng S, Janani L, Cornelius V, Aley PK, Babbage G, et al. Safety, immunogenicity, and reactogenicity of BNT162b2 and mRNA-1273 COVID-19 vaccines given as fourth-dose boosters following two doses of ChAdOx1 nCoV-19 or BNT162b2 and a third dose of BNT162b2 (COV-BOOST): a multicentre, blinded, phase 2, randomised trial. 2022;22(8):1131-41.
245. Munro APS, Janani L, Cornelius V, Aley PK, Babbage G, Baxter D, et al. Safety and immunogenicity of seven COVID-19 vaccines as a third dose (booster) following two doses of ChAdOx1 nCov-19 or BNT162b2 in the UK (COV-BOOST): a blinded, multicentre, randomised, controlled, phase 2 trial. 2021;398(10318):2258-76.
246. N B, N D, Y B-S, E K, J W, R O, et al. Safety of the BNT162b2 mRNA Covid-19 Vaccine in a Nationwide Setting. The New England journal of medicine. 2021;385(12):1078-90.
247. N M, C H, C M, T M, H H, S T, et al. Review of a Series of Surveys on Adverse Reactions to the COVID-19 mRNA-1273 Vaccine at Okayama University. Acta medica Okayama. 2023;77(6):567-75.
248. Najjar M, Albuaini S, Fadel M, Mohsen F. Covid-19 vaccination reported side effects and hesitancy among the Syrian population: a cross-sectional study. 2023;55(2).
249. Namiki T, Komine-Aizawa S, Takada K, Takano C, Trinh QD, Hayakawa S. Adverse events after BNT162b2 mRNA COVID-19 vaccination in health care workers and medical students in Japan. JOURNAL OF INFECTION AND CHEMOTHERAPY. 2022;28(8).
250. Nasergivehchi S, Togha M, Jafari E, Sheikhvatan M, Shahamati D. Headache following vaccination against COVID-19 among healthcare workers with a history of COVID-19 infection: a cross-sectional study in Iran with a meta-analytic review of the literature. 2023;19(1).
251. Noh Y, Kim JH, Yoon D, Choe YJ, Choe S-A, Jung J, et al. Predictors of COVID-19 booster vaccine hesitancy among fully vaccinated adults in Korea: a nationwide cross-sectional survey. EPIDEMIOLOGY AND HEALTH. 2022;44.
252. Noh Y, Ko HY, Kim JH, Yoon D, Choe YJ, Choe S-A, et al. Barriers to COVID-19 vaccine surveillance: the issue of under-reporting adverse events. 2023;45:1-7.
253. Noureldine HA, Maamari J, El Helou MO, Chedid G, Farra A, Husni R, et al. The effect of the BNT162b2 vaccine on antinuclear antibody and antiphospholipid antibody levels. IMMUNOLOGIC RESEARCH. 2022;70(6):800-10.
254. Nunez I, Garcia-Grimshaw M, Castillo Valencia CY, Aguilera Callejas DE, Moya Alfaro ML, Del Mar Saniger-Alba M, et al. Seizures following COVID-19 vaccination in Mexico: A nationwide observational study. EPILEPSIA. 2022;63(10):E144-E9.
255. Nuzzo A, Manacorda S, Sammarco E, Sbrana A, Bazzurri S, Paolieri F, et al. Safety and Tolerability of COVID-19 Vaccines in Patients with Cancer: A Single Center Retrospective Analysis. VACCINES. 2022;10(6).

256. Ochoa-Azze R, Chang-Monteagudo A, Climent-Ruiz Y, Macías-Abraham C, Valenzuela-Silva C, de los Ángeles García-García M, et al. Safety and immunogenicity of the FINLAY-FR-1A vaccine in COVID-19 convalescent participants: an open-label phase 2a and double-blind, randomised, placebo-controlled, phase 2b, seamless, clinical trial. 2022;10(8):785-95.
257. Oda Y, Kumagai Y, Kanai M, Iwama Y, Okura I, Minamida T, et al. Immunogenicity and safety of a booster dose of a self-amplifying RNA COVID-19 vaccine (ARCT-154) versus BNT162b2 mRNA COVID-19 vaccine: a double-blind, multicentre, randomised, controlled, phase 3, non-inferiority trial. Lancet Infect Dis. 2024;24(4):351-60.
258. Odeh M, Al-Jussani GN, Ashour A, AlNaqah H, Hasan HA, Sbitan L, et al. Follow-Up of Side Effects throughout the Entire Course of Coronavirus Vaccination. 2023;11(3).
259. Olana S, Mazzilli R, Salerno G, Zamponi V, Tarsitano MG, Simmaco M, et al. 4BNT162b2 mRNA COVID-19 vaccine and semen: What do we know? 2022;10(6):1023-9.
260. Ossato A, Tessari R, Trabucchi C, Zuppini T, Realdon N, Marchesini F. Comparison of medium-term adverse reactions induced by the first and second dose of mRNA BNT162b2 (Comirnaty, Pfizer-BioNTech) vaccine: a post-marketing Italian study conducted between 1 January and 28 February 2021. EUROPEAN JOURNAL OF HOSPITAL PHARMACY. 2023;30(4).
261. Oudjedi A, Allali A, Bekli A, Lounis M, Ben Saad H, Boukoufa M. Reported COVID-19 vaccines side effects among Algerian athletes: a comparison between inactivated virus, adenoviral vector, and mRNA COVID-19 vaccines. 2024;52(2):134-46.
262. Oyebanji OA, Wilson B, Keresztesy D, Carias L, Wilk D, Payne M, et al. Does a lack of vaccine side effects correlate with reduced BNT162b2 mRNA vaccine response among healthcare workers and nursing home residents? 2021;33(11):3151-60.
263. Pai M-F, Tung K-T, Hsu S-P, Peng Y-S, Lin W-Y, Yang J-Y, et al. Adverse events following the first, second and third doses of a COVID-19 vaccine in hemodialysis patients. RENAL FAILURE. 2023;45(1).
264. Pakhchanian H, Raiker R, Wolf M, Trotter SC. Examining the risk of breakthrough infection and COVID-19 vaccination safety in patients with atopic dermatitis. 2022;187(2):251-3.
265. Pani A, Cento V, Vismara C, Campisi D, Di Ruscio F, Romandini A, et al. Results of the RENAISSANCE Study: REsponse to BNT162b2 COVID-19 vacciNe—short- And long-term Immune reSponSe evAluatioN in health Care workErs. 2021;96(12):2966-79.
266. Paran Y, Saiag E, Spitzer A, Angel Y, Yakubovsky M, Padova H, et al. Short-Term Safety of Booster Immunization With BNT162b2 mRNA COVID-19 Vaccine in Healthcare Workers. OPEN FORUM INFECTIOUS DISEASES. 2022;9(3).
267. Park C, Sakong J, Jo S, Kim M, Baek K. Adverse effects on work and daily life interference among healthcare workers after the first and second chadox1 and bnt162b2 covid-19 vaccine doses. 2021;9(8).
268. Park J, Kim D, Song T-J. A Disproportionality Analysis for Association of Systemic Capillary Leak Syndrome with COVID-19 Vaccination Using the World Health Organization Pharmacovigilance Database. VACCINES. 2022;10(6).
269. Park YH, Kim MH, Choi MG, Chun EM. Correlation between COVID-19 vaccination and inflammatory musculoskeletal disorders. medRxiv. 2023.
270. Pasternak G, Pieniawska-Śmiech K, Walkowiak M, Sado J, Pytel A, Jasińska P, et al. Before and After: Attitude and Adverse Effects Induced by the First and Second Doses of mRNA BNT162b2 Vaccine among Healthcare Professionals in the First Weeks after Their Introduction in Poland. 2023;11(5).
271. Payne A, B. a, Novosad S, Wieg, RE, Najdowski M, et al. Effectiveness of Bivalent mRNA COVID-19 Vaccines in Preventing COVID-19-Related Thromboembolic Events Among Medicare Enrollees Aged ≥65 Years and Those with End Stage Renal Disease - United States, September 2022-March 2023. MMWR-MORBIDITY AND MORTALITY WEEKLY REPORT. 2024;73(1):16-23.
272. Peretz SB, Regev N, Novick L, Nachshol M, Goffer E, Ben-David A, et al. Short-term outcome of pregnant women vaccinated with BNT162b2 mRNA COVID-19 vaccine. ULTRASOUND IN OBSTETRICS \& GYNECOLOGY. 2021;58(3):450-6.
273. Perez-Campuzano V, Pe R, Marjot T, Praktiknjo M, Alvarado-Tapias E, Turco L, et al. Efficacy and safety of vaccination againts SARS-CoV-2 in patients with vascular liver disease. J Hepatol. 2023;78:970-1.
274. Perkhofer L, Nägele J, Kroschel J, Mayer B, Müller M, Seufferlein T. Acute reactions after vaccination against COVID-19 and long-term antibody levels. 2022;119(27):484-5.
275. PIEROBON E, SECHI A, GERMI l, PEZZOLO E, TREVISAN G, FELICIANI C, et al. Severe injection site reaction ensuing the first dose of mRNA COVID-19 vaccine. ITALIAN JOURNAL OF DERMATOLOGY AND VENEREOLOGY. 2022;157(3):281-2.

276. Pires C. Serious Adverse Reactions to COVID-19 vaccines in Portugal up to July 2021: thrombosis with thrombocytopenia syndrome, myocarditis/pericarditis, and Guillain-Barre syndrome. 2022;19(1).
277. Polack F, P. o, Thomas SJ, Kitchin N, Absalon J, Gurtman A, et al. Safety and Efficacy of the BNT162b2 mRNA Covid-19 Vaccine. NEW ENGLAND JOURNAL OF MEDICINE. 2020;383(27):2603-15.
278. Pollock KM, Cheeseman HM, Szubert AJ, Libri V, Boffito M, Owen D, et al. Safety and immunogenicity of a self-amplifying RNA vaccine against COVID-19: COVAC1, a phase I, dose-ranging trial. 2022;44.
279. Ponticelli D, Madotto F, Conti S, Antonazzo IC, Vitale A, Della Ragione G, et al. Response to BNT162b2 mRNA COVID-19 vaccine among healthcare workers in Italy: a 3-month follow-up. INTERNAL AND EMERGENCY MEDICINE. 2022;17(2):481-6.
280. Powell AA, Power L, Westrop S, McOwat K, Campbell H, Simmons R, et al. Real-world data shows increased reactogenicity in adults after heterologous compared to homologous prime-boost COVID-19 vaccination, March-June 2021, England. EUROSURVEILLANCE. 2021;26(28).
281. Raethke M, van Hunsel F, Thurin NH, Dureau-Pournin C, Mentzer D, Kovačić B, et al. Cohort Event Monitoring of Adverse Reactions to COVID-19 Vaccines in Seven European Countries: Pooled Results on First Dose. 2023;46(4):391-404.
282. Raj S, Ogola G, Han J. COVID-19 Vaccine-Associated Subclinical Axillary Lymphadenopathy on Screening Mammogram. ACADEMIC RADIOLOGY. 2022;29(4):501-7.
283. Rechtien L, Erfurt-Berge C, Sticherling M. SCLE manifestation after mRNA COVID-19 vaccination. 2022;36(4):e261-e3.
284. Reynolds MW, Xie Y, Knuth KB, Mack CD, Brinkley E, Toovey S, et al. COVID-19 Vaccination Breakthrough Infections in a Real-World Setting: Using Community Reporters to Evaluate Vaccine Effectiveness. 2022;15:5167-82.
285. Riad A, Hockova B, Kantorova L, Slavik R, Spurna L, Stebel A, et al. Side Effects of mRNA-Based COVID-19 Vaccine: Nationwide Phase IV Study among Healthcare Workers in Slovakia. PHARMACEUTICALS. 2021;14(9).
286. Riad A, Issa J, Attia S, Dusek L, Klugar M. Oral adverse events following COVID-19 and influenza vaccination in Australia. HUMAN VACCINES \& IMMUNOTHERAPEUTICS. 2023;19(2).
287. Rodríguez Y, Rojas M, Beltrán S, Polo F, Camacho-Domínguez L, Morales SD, et al. Autoimmune and autoinflammatory conditions after COVID-19 vaccination. New case reports and updated literature review. J Autoimmun. 2022;132.
288. Rodríguez-Ferreras A, Maray I, Coya-Fernández C, Octavio-Bocigas MM, Fernández-Del Río MF, Casares-López S, et al. Kikuchi-Fujimoto Disease and COVID-19 vaccination: pharmacovigilance approach. 2023;55(6):278-82.
289. Rolfes L, Härmark L, Kant A, van Balveren L, Hilgersom W, van Hunsel F. COVID-19 vaccine reactogenicity – A cohort event monitoring study in the Netherlands using patient reported outcomes. 2022;40(7):970-6.
290. Romero-Ibarguengoitia ME, González-Cantú A, Rivera-Salinas D, Hernández-Ruíz YG, Armendariz-Vázquez AG, Barco-Flores IA, et al. Analysis of Immunization, Adverse Events, and Efficacy of a Fourth Dose of BNT162b2 Vaccine in Health Workers in Mexico, a Pilot Study. 2022;10(7).
291. Rosso A, Flacco ME, Soldato G, Di Martino G, Martellucci CA, Carota R, et al. COVID-19 Vaccination Effectiveness in the General Population of an Italian Province: Two Years of Follow-Up. VACCINES. 2023;11(8).
292. Rottenstreich M, Rotem R, Wiener-Well Y, Grisaru-Granovsky S, Sela HY. Covid-19 third vaccination during pregnancy: maternal and neonatal outcomes—a retrospective study. 2023;308(4):1197-205.
293. Tsang RSM, Joy M, Byford R, Robertson C, Anand SN, Hinton W, et al. Adverse events following first and second dose COVID-19 vaccination in England, October 2020 to September 2021: a national vaccine surveillance platform self-controlled case series study. 2023;28(3).
294. Ruggiero R, Balzano N, Di Napoli R, Mascolo A, Berrino PM, Rafaniello C, et al. Capillary leak syndrome following COVID-19 vaccination: Data from the European pharmacovigilance database Eudravigilance. FRONTIERS IN IMMUNOLOGY. 2022;13.
295. Rzymski P, Sikora D, Zeyl, J, Poniedzialek B, Kiedik D, et al. Frequency and Nuisance Level of Adverse Events in Individuals Receiving Homologous and Heterologous COVID-19 Booster Vaccine. VACCINES. 2022;10(5).
296. S DC, MT L-S, M C, R M, F C, C S, et al. Safety of third dose of COVID-19 vaccination in frail patients: Results from the prospective Italian VAX4FRAIL study. Frontiers in oncology. 2022;12:1002168.

297. S LV, M B, J B, MJ J, J D, L S, et al. Risk of Guillain-Barré Syndrome Following COVID-19 Vaccines: A Nationwide Self-Controlled Case Series Study. Neurology. 2023;101(21):e2094-e102.
298. S S, AK S, J P, F S, M B, J T, et al. Which vaccine attributes foster vaccine uptake? A cross-country conjoint experiment. PloS one. 2022;17(5):e0266003.
299. Sa S, Lee CW, Shim SR, Yoo H, Choi J, Kim JH, et al. The Safety of mRNA-1273, BNT162b2 and JNJ-78436735 COVID-19 Vaccines: Safety Monitoring for Adverse Events Using Real-World Data. VACCINES. 2022;10(2).
300. Sacchi MC, Pelazza C, Bertolotti M, Agatea L, De Gaspari P, Tamiazzo S, et al. The onset of <i>de novo</i> autoantibodies in healthcare workers after mRNA based anti-SARS-CoV-2 vaccines: a single centre prospective follow-up study. AUTOIMMUNITY. 2023;56(1).
301. Sadat Larijani M, Sorouri R, Eybpoosh S, Doroud D, Moradi L, Ahmadinezhad M, et al. Assessment of long-term adverse events regarding different COVID-19 vaccine regimens within an 18-month follow-up study. 2023;81.
302. Said EA, Al-Rubkhi A, Jaju S, Koh CY, Al-Balushi MS, Al-Naamani K, et al. Association of the Magnitude of Anti-SARS-CoV-2 Vaccine Side Effects with Sex, Allergy History, Chronic Diseases, Medication Intake, and SARS-CoV-2 Infection. 2024;12(1).
303. Saito K, Ichikawa S, Hatta S, Katsuoka Y, Harigae H, Izumi T. Vincristine therapy for severe and refractory immune thrombocytopenia following COVID-19 vaccination. 2022;101(4):885-7.
304. Sakamoto A, Yoshimura M, Itoh R, Ozuru R, Ishii K, Sechi Y, et al. Longitudinal Dynamics of SARS-CoV-2 IgG Antibody Responses after the Two-Dose Regimen of BNT162b2 Vaccination and the Effect of a Third Dose on Healthcare Workers in Japan. VACCINES. 2022;10(6).
305. Salvo F, Pariente A, Valnet-Rabier MB, Drici M-D, Cholle C, Mathieu C, et al. Role of spontaneous reporting in investigating the relationship between mRNA COVID-19 vaccines and myocarditis: The French perspective. THERAPIE. 2023;78(5):509-15.
306. Sanjay S, Handa A, Kawali A, Shetty R, Bhakti Mishra S, Mahendradas P. Scleritis and Episcleritis following Coronavirus Disease (COVID-19) Vaccination. 2023;31(6):1184-90.
307. Sari US, Dulger D, Yolcu A, Hekimoglu CH. Acute Adverse Events After BNT162b2 mRNA and Inactivated SARS-CoV-2 Vaccines in People who had COVID-19 Vaccines. KLIMIK JOURNAL. 2022;35(4):230-7.
308. SC L, NN B, R S, K E, J P, JC K, et al. A Population-Based Test-Negative Matched Case-Control Analysis of SARS-CoV-2 Vaccine Effectiveness Among Pregnant People in Ontario, Canada. Journal of obstetrics and gynaecology Canada : JOGC = Journal d'obstetrique et gynecologie du Canada : JOGC. 2024;46(2):102239.
309. Scher AI, Berjohn CM, Byrne C, Colombo RE, Colombo CJ, Edwards MS, et al. Variation by Type, Dose, and History, Severity, and Recency of Prior SARS-CoV-2 Infection. Open Forum Infect Dis. 2022;9(7).
310. Serrano-Arias B, Ferrara F, Zavaleta E, Zovi A, Ortiz-Barboza A, Pasquinucci R, et al. Adverse Events Following mRNA COVID-19 Vaccine in 2021 and 2022: A Retrospective Analysis in Costa Rica and Italy. CUREUS JOURNAL OF MEDICAL SCIENCE. 2023;15(10).
311. SH K, DK Y, YS C, J L, KH P, YJ L, et al. Vertigo and Dizziness After Coronavirus Disease-2019 Vaccination: A Nationwide Analysis. The journal of international advanced otology. 2023;19(3):228-33.
312. Shabu A, Nishtala PS. Analysis of the adverse events following the mRNA-1273 COVID-19 vaccine. EXPERT REVIEW OF VACCINES. 2023;22(1):801-12.
313. Shasha D, Bareket R, Sikron FH, Gertel O, Tsamir J, Dvir D, et al. Real-world safety data for the Pfizer BNT162b2 SARS-CoV-2 vaccine: historical cohort study. CLINICAL MICROBIOLOGY AND INFECTION. 2022;28(1):130-4.
314. Shaw CA, Essink B, on, Harper C, Mithani R, Kapoor A, et al. Safety and Immunogenicity of an mRNA-Based RSV Vaccine Including a 12-Month Booster in a Phase 1 Clinical Trial in Healthy Older Adults. JOURNAL OF INFECTIOUS DISEASES. 2024.
315. Shechtman L, Lahad K, Livneh A, Grossman C, Druyan A, Giat E, et al. Safety of the BNT162b2 mRNA COVID-19 vaccine in patients with familial Mediterranean fever. RHEUMATOLOGY. 2022;61:SI129-SI35.
316. Shoaibi A, Lloyd PC, Wong H-L, Clarke TC, Chillarige Y, Do R, et al. Evaluation of Potential Adverse Events Following COVID-19 mRNA Vaccination Among Adults Aged 65 Years and Older: A Self-Controlled Study in the U.S. medRxiv. 2023.

317. Shrotri M, Krutikov M, Nacer-Laidi H, Azmi B, Palmer T, Giddings R, et al. Duration of vaccine effectiveness against SARS-CoV-2 infection, hospitalisation, and death in residents and staff of long-term care facilities in England (VIVALDI): a prospective cohort study. 2022;3(7):e470-e80.
318. Siangphoe U, Baden LR, El Sahly HM, Essink B, Ali K, Berman G, et al. Associations of Immunogenicity and Reactogenicity After Severe Acute Respiratory Syndrome Coronavirus 2 mRNA-1273 Vaccine in the COVE and TeenCOVE Trials. 2023;76(2):271-80.
319. Slizien Z, Muchlado M, Kubanek A, Biedunkiewicz B, Renke M, Komorowska K, et al. Safety and Tolerability of mRNA COVID-19 Vaccines in Kidney Transplant Recipients. TRANSPLANTATION PROCEEDINGS. 2022;54(4):878-83.
320. Son C-S, Jin S-H, Kang W-S. Propensity-Score-Matched Evaluation of Adverse Events Affecting Recovery after COVID-19 Vaccination: On Adenovirus and mRNA Vaccines. VACCINES. 2022;10(2).
321. Speletas M, Voulgaridi I, Sarrou S, Dadouli A, Mouchtouri VA, Nikoulis DJ, et al. Intensity and Dynamics of Anti-SARS-CoV-2 Immune Responses after BNT162b2 mRNA Vaccination: Implications for Public Health Vaccination Strategies. VACCINES. 2022;10(2).
322. Sriphoosanaphan S, Suksawatamnuay S, Srisoonthorn N, Siripon N, Thaimai P, Makhasen W, et al. IMMUNOGENICITY AND SAFETY OF HETEROLOGOUS VERSUS HOMOLOGOUS PRIME-BOOST REGIMENS WITH ADENOVIRAL VECTORED AND MRNA SARS-COV-2 VACCINE IN LIVER TRANSPLANT RECIPIENTS. Gastroenterology. 2022;162(7):S-1252.
323. St Clair B, Hoffman D, Lee R, Montgomery J. MRNA COVID-19 VACCINE ADVERSE EVENTS FOLLOWING IMMUNIZATION: SHOULD YOU RECOMMEND THE SECOND DOSE? ANNALS OF ALLERGY ASTHMA \& IMMUNOLOGY. 2021;127(5):S3.
324. Stehlin F, Tommasini F, Monzambani-B, eret V, Girard C, Yerly D, et al. Graded-dosing immunization in adults at risk for immediate-type reactions to mRNA SARS-CoV-2 vaccines. ALLERGOLOGY INTERNATIONAL. 2023;72(2):332-4.
325. Stochino ME, Ambu G, Anania L, Boccalini A, Cau EE, Congiu A, et al. Covid-19 Vaccines and Menstrual Disorders: Reports from Sardinian Regional Centre of Pharmacovigilance. Drug Saf. 2022;45(10):1193-4.
326. Stone Jr CA, Robinson LB, Li L, Krantz MS, Kwah JH, Ortega G, et al. Clinical Phenotypes of Immediate First-Dose Reactions to mRNA COVID-19: A Multicenter Latent Class Analysis. JOURNAL OF ALLERGY AND CLINICAL IMMUNOLOGY-IN PRACTICE. 2023;11(2):458+.
327. Strobel SB, Machiraju D, Kälber KA, Hassel JC. Immune-related adverse events of COVID-19 vaccination in skin cancer patients receiving immune-checkpoint inhibitor treatment. 2022;71(8):2051-6.
328. Stuart ASV, Shaw RH, Liu X, Greenland M, Aley PK, Andrews NJ, et al. Immunogenicity, safety, and reactogenicity of heterologous COVID-19 primary vaccination incorporating mRNA, viral-vector, and protein-adjuvant vaccines in the UK (Com-COV2): a single-blind, randomised, phase 2, non-inferiority trial. 2022;399(10319):36-49.
329. Sultana A, Shahriar S, Tahsin MR, Mim SR, Fatema KR, Saha A, et al. A retrospective cross-sectional study assessing self-reported adverse events following immunization (AEFI) of the COVID-19 vaccine in Bangladesh. 2021;9(10).
330. Swierkot J, Madej M, Szmyrka M, Korman L, Sokolik R, Andrasiak I, et al. The Risk of Autoimmunity Development following mRNA COVID-19 Vaccination. VIRUSES-BASEL. 2022;14(12).
331. T A, F A, NS B, AH A, SA A, KF A, et al. Analyzing COVID-19 Vaccination Side Effects Among the Adult Population in Jeddah, Saudi Arabia. Cureus. 2023;15(10):e47136.
332. T A, T O, T T, H H, T I. Acute Adverse Events at a Mass Vaccination Site after the Third and Fourth COVID-19 Vaccinations in Japan. The Tohoku journal of experimental medicine. 2023;259(4):263-71.
333. Tadfor Y, Nguyen-Hua N, Bennett H, Bos A, J. eJ, Smolinske SCC. An observational study of adverse drug reactions to COVID-19 vaccines reported to the New Mexico poison center hotline. CLINICAL TOXICOLOGY. 2023;61(1):77-83.
334. Takeuchi Y, Iwagami M, Ono S, Michihata N, Uemura K, Yasunaga H. A post-marketing safety assessment of COVID-19 mRNA vaccination for serious adverse outcomes using administrative claims data linked with vaccination registry in a city of Japan. 2022;40(52):7622-30.
335. Tamburro M, Ripabelli G, D'Amico A, De Dona R, Iafigliola M, Parente A, et al. A Cross-Sectional Study of Untoward Reactions Following Homologous and Heterologous COVID-19 Booster Immunizations in Recipients Seventeen Years of Age and Older. JOURNAL OF COMMUNITY HEALTH. 2022;47(5):814-21.

336. Tan AY, Chang CT, Yu YK, Low YX, Razali NFM, Tey SY, et al. Adverse Events Following BNT162b2 mRNA COVID-19 Vaccine Immunization among Healthcare Workers in a Tertiary Hospital in Johor, Malaysia. VACCINES. 2022;10(4).
337. Tan JTC, Tan C, Teoh J, Wahab MT, Tan GZ, Chin RYZ, et al. Adverse reactions and safety profile of the mRNA COVID-19 vaccines among Asian military personnel. ANNALS ACADEMY OF MEDICINE SINGAPORE. 2021;50(11).
338. Tanzilli A, Pace A, Ciliberto G, La Malfa AM, Buonomo V, Benincasa D, et al. COV-BT Ire study: safety and efficacy of the BNT162b2 mRNA COVID-19 vaccine in patients with brain tumors. NEUROLOGICAL SCIENCES. 2022;43(6):3519-22.
339. Tenforde MW, Self WH, Zhu Y, Naioti EA, Gaglani M, Ginde AA, et al. Protection of Messenger RNA Vaccines Against Hospitalized Coronavirus Disease 2019 in Adults Over the First Year Following Authorization in the United States. CLINICAL INFECTIOUS DISEASES. 2023;76(3):E460-E8.
340. Terezia H, Zuzana K, Petr N. Evaluation of SARS-CoV-2 vaccination in pregnant and breastfeeding women. 2023;8:58-63.
341. Terpos E, Trougakos IP, Karalis V, Ntanasis-Stathopoulos I, Apostolakou F, Gumeni S, et al. Kinetics of anti-SARS-CoV-2 antibody responses 3 months post complete vaccination with BNT162B2; a prospective study in 283 health workers. Blood. 2021;138:4202.
342. Thai-Van H, Valnet-Rabier M-B, Anciaux M, Lambert A, Maurier A, Cottin J, et al. Safety Signal Generation for Sudden Sensorineural Hearing Loss Following Messenger RNA COVID-19 Vaccination: Postmarketing Surveillance Using the French Pharmacovigilance Spontaneous Reporting Database. JMIR PUBLIC HEALTH AND SURVEILLANCE. 2023;9.
343. Theodorou DJ, Theodorou SJ, Rizos D. Major cardiovascular events after COVID-19, event rates post-vaccination, antiviral or anti-inflammatory therapy, and temporal trends: Rationale and methodology of the CORONA-VTE-Network study. 2023;232:146-7.
344. Thompson MG, Burgess JL, Naleway AL, Tyner H, Yoon SK, Meece J, et al. Prevention and Attenuation of Covid-19 with the BNT162b2 and mRNA-1273 Vaccines. NEW ENGLAND JOURNAL OF MEDICINE. 2021;385(4):320-9.
345. Thurm C, Reinhold A, Borucki K, Kahlfuss S, Feist E, Schreiber J, et al. Homologous and Heterologous Anti-COVID-19 Vaccination Does Not Induce New-Onset Formation of Autoantibodies Typically Accompanying Lupus Erythematodes, Rheumatoid Arthritis, Celiac Disease and Antiphospholipid Syndrome. Vaccines. 2022;10(2).
346. TR M, PL M, JM G, AM H, L P, B Z, et al. The v-safe after vaccination health checker: Active vaccine safety monitoring during CDC's COVID-19 pandemic response. Vaccine. 2023;41(7):1310-8.
347. Tsai C, Chung W, Chen C. 227 Immune response and allergenic components of COVID-19 vaccines induced delayed cutaneous reactions. J Invest Dermatol. 2023;143(5):S39.
348. Tsoi JYH, Cai J, Situ J, Lam WJ, Shun EHK, Leung JKY, et al. Autoantibodies against angiotensin-converting enzyme 2 (ACE2) after COVID-19 infection or vaccination. JOURNAL OF MEDICAL VIROLOGY. 2023;95(12).
349. Urakawa R, Isomura ET, Matsunaga K, Kubota K. Young Age, Female Sex, and No Comorbidities Are Risk Factors for Adverse Reactions after the Third Dose of BNT162b2 COVID-19 Vaccine against SARS-CoV-2: A Prospective Cohort Study in Japan. 2022;10(8).
350. Urakawa R, Isomura ET, Matsunaga K, Kubota K. Multivariate Analysis of Adverse Reactions and Recipient Profiles in COVID-19 Booster Vaccinations: A Prospective Cohort Study. 2023;11(10).
351. V F, P E, H C, LE R, F L, B G, et al. [Pharmacovigilance - mRNA COVID-19 vaccines : current state of knowledge on their adverse effects]. Revue medicale suisse. 2022;18(767):190-7.
352. V F, T E, D Z, H P, D P, D L, et al. Immunogenicity and safety of the BNT162b2 mRNA COVID-19 vaccine in adult patients with autoimmune inflammatory rheumatic diseases and in the general population: a multicentre study. Annals of the rheumatic diseases. 2021;80(10):1330-8.
353. Valcarce V, Stafford LS, Neu J, Parker L, Vicuna V, Cross T, et al. COVID-19 booster enhances IgG mediated viral neutralization by human milk in vitro. 2024;11.
354. Valera-Rubio M, Isabel Sierra-Torres M, Castillejo Garcia R, Cordero-Ramos J, Reyes Lopez-Marquez M, Cruz-Salgado O, et al. Adverse events reported after administration of BNT162b2 and mRNA-1273 COVID-19 vaccines among hospital workers: a cross-sectional survey-based study in a Spanish hospital. EXPERT REVIEW OF VACCINES. 2022;21(4):533-40.

355. van der Boom MDX, van Eekeren R, van Hunsel FPAM. Observed-over-Expected analysis as additional method for pharmacovigilance signal detection in large-scaled spontaneous adverse event reporting. 2023;32(7):783-94.
356. van der Boor SC, Schmitz-de Vries ETJ, Smits D, Scholl JHG, Rolfes L, van Hunsel F. Spontaneously reported adverse events following COVID-19 basic and booster immunizations in the Netherlands. 2023;41(29):4319-26.
357. Vieira J, Marcelino J, Ferreira F, Proença M, Guimarães J, Tomaz E. Adverse reactions to COVID-19 vaccines: Differences and similarities. Allergy Eur J Allergy Clin Immunol. 2023;78:332.
358. Villanueva P, McDonald E, Croda J, Croda MG, Dalcolmo M, dos Santos G, et al. Factors influencing adverse events following COVID-19 vaccination. 2024;20(1).
359. Walle T, Bajaj S, Kraske JA, Rösner T, Cussigh CS, Kälber KA, et al. Cytokine release syndrome-like serum responses after COVID-19 vaccination are frequent and clinically inapparent under cancer immunotherapy. 2022;3(9):1039-51.
360. Walton M, Pletzer V, Teunissen T, Lumley T, Hanlon T. Adverse Events Following the BNT162b2 mRNA COVID-19 Vaccine (Pfizer-BioNTech) in Aotearoa New Zealand. DRUG SAFETY. 2023;46(9):867-79.
361. Wan EYF, Chui CSL, Mok AHY, Xu W, Yan VKC, Lai FTT, et al. mRNA (BNT162b2) and Inactivated (CoronaVac) COVID-19 Vaccination and Risk of Adverse Events and Acute Diabetic Complications in Patients with Type 2 Diabetes Mellitus: A Population-Based Study. 2022;45(12):1477-90.
362. Werner F, Zeschick N, Kuehlein T, Steininger P, Ueberla K, Kaiser I, et al. Patient-reported reactogenicity and safety of COVID-19 vaccinations vs. comparator vaccinations: a comparative observational cohort study. BMC MEDICINE. 2023;21(1).
363. Wi Y-M, Kim S-H, Peck K-R. Early adverse events between mrna and adenovirus-vectored covid-19 vaccines in healthcare workers. 2021;9(8).
364. Widman AJ, Cohen B, Park V, McClure T, Wolchok J, Kamboj M. Immune-Related Adverse Events among COVID-19–Vaccinated Patients with Cancer Receiving Immune Checkpoint Blockade. 2022;20(10):1134-8.
365. WJ S, K AC, JH C, TA W, SF C, YC C, et al. Acute reactions after a homologous primary COVID-19 vaccination series: Analysis of Taiwan V-Watch data. Vaccine. 2023;41(17):2853-9.
366. Wong H-L, Tworkoski E, Zhou CK, Hu M, Thompson D, Lufkin B, et al. Surveillance of COVID-19 vaccine safety among elderly persons aged 65 years and older. VACCINE. 2023;41(2):532-9.
367. WP B, D R, P V-B, M T, L L, B T, et al. Association of Self-reported Systemic Reactions Following SARS-CoV-2 Vaccination With Immunological Response in the Danish National Cohort Study of Effectiveness and Safety of SARS-CoV-2 Vaccines (ENFORCE). Open forum infectious diseases. 2023;10(6):ofad248.
368. Wu G, Zhang M, Xie X, Zhu Y, Tang H, Zhu X, et al. A survey on the safety of the SARS-CoV-2 vaccine among a population with stroke risk in China. 2022;9.
369. Wu J-D, Li J-X, Liu J, Wang H-M, Zhou G-H, Li J, et al. Safety, immunogenicity, and efficacy of the mRNA vaccine CS-2034 as a heterologous booster versus homologous booster with BBIBP-CorV in adults aged ≥18 years: a randomised, double-blind, phase 2b trial. Lancet Infect Dis. 2023;23(9):1020-30.
370. Xiong X, Wong CKH, Au ICH, Lai FTT, Li X, Wan EYF, et al. Safety of Inactivated and mRNA COVID-19 Vaccination Among Patients Treated for Hypothyroidism: A Population-Based Cohort Study. THYROID. 2022;32(5):505-14.
371. Xu S, Hong V, Sy LS, Glenn SC, Ryan DS, Morrissette KL, et al. Changes in incidence rates of outcomes of interest in vaccine safety studies during the COVID-19 pandemic. 2022;40(23):3150-8.
372. Xu W, Wen X, Cong X, Jiang W. COVID-19 mRNA vaccine, but not a viral vector-based vaccine, promotes neutralizing anti-type I interferon autoantibody production in a small group of healthy individuals. J Med Virol. 2023;95(10).
373. Y K, I H, M K, H K, S K, SY S, et al. Self-reported adverse events after 2 doses of COVID-19 vaccine in Korea. Epidemiology and health. 2023;45:e2023006.
374. Yagci AK, Alci G, Guncu MM, Yllmaz OB, Tekin E, Cakir SE, et al. Demographic Features and Laboratory Parameters Among Hospitalized Vaccinated Patients With COVID-19 in Istanbul, Turkey. INFECTIOUS DISEASES IN CLINICAL PRACTICE. 2023;31(3).
375. Yalcinkaya A, Cavalli M, Cederholm A, Ar, a-Guillen M, Behere A, et al. No link between type I interferon autoantibody positivity and adverse reactions to COVID-19 vaccines. NPJ VACCINES. 2024;9(1).
376. Yan M-M, Zhao H, Li Z-R, Chow J-W, Zhang Q, Qi Y-P, et al. Serious adverse reaction associated with the COVID-19 vaccines of BNT162b2, Ad26.COV2.S, and mRNA-1273: Gaining insight through the VAERS. FRONTIERS IN PHARMACOLOGY. 2022;13.

377. Yan VKC, Wan EYF, Ye X, Mok AHY, Lai FTT, Chui CSL, et al. Waning effectiveness against COVID-19-related hospitalization, severe complications, and mortality with two to three doses of CoronaVac and BNT162b2: a case–control study. 2023;12(1).
378. Yasri S, Wiwanitkit V. Expected Usefulness of Fourth Dose of COVID-19 Vaccine for Patients with Underlying Solid Tumor who Previously Received the Primary Heterologous COVID-19 Vaccine. INDIAN JOURNAL OF MEDICAL AND PAEDIATRIC ONCOLOGY. 2024.
379. Ye X, Ye W, Yu J, Gao Y, Ren Z, Chen L, et al. The landscape of COVID-19 vaccination among healthcare workers at the first round of COVID-19 vaccination in China: willingness, acceptance and self-reported adverse effects. 2021;17(12):4846-56.
380. Yendewa GA, Perez JA, Patil N, McComsey GA. Associations between post-acute sequelae of SARS-CoV-2, COVID-19 vaccination and HIV infection: a United States cohort study. 2024;15.
381. Yih WK, Daley MF, Duffy J, Fireman B, McClure D, Nelson J, et al. A broad assessment of covid-19 vaccine safety using tree-based data-mining in the vaccine safety datalink. VACCINE. 2023;41(3):826-35.
382. Yoshida M, Kobashi Y, Kawamura T, Shimazu Y, Nishikawa Y, Omata F, et al. Association of systemic adverse reaction patterns with long-term dynamics of humoral and cellular immunity after coronavirus disease 2019 third vaccination. SCIENTIFIC REPORTS. 2023;13(1).
383. Z N, L M, S U, H A, L N. A prospective observational study to evaluate the safety of COVID-19 mRNA vaccines administered to Qatar Rehabilitation Institute patients. Qatar medical journal. 2023;2023(1):10.
384. Zahradka I, Petr V, Modos I, Magicova M, Dusek L, Viklicky O. Association Between SARS-CoV-2 Messenger RNA Vaccines and Lower Infection Rates in Kidney Transplant Recipients A Registry-Based Report. ANNALS OF INTERNAL MEDICINE. 2022;175(7):961+.
385. Zeitoun A, Hallit S, Chehade S, Ibrahim A, Helali M, Allam C, et al. A 1-year analysis of adverse events following COVID-19 vaccination in Lebanon: a retrospective study. 2023;16(1).
386. Zenilman J, Belshe R, Edwards K, Self S, Stanberry L, Newell T, et al. Safety and efficacy of the BNT162B2 mRNA covid-19 vaccine through 6 months. 2021;385(19):1761-73.
387. Zhang XS, Moreau A, Cruz-Santiago D, Langevin S, Nguyen QD. Safety and Adverse Events Among Long-term Care Residents Receiving a Third COVID-19 mRNA Vaccine Booster Dose in Quebec. JAMA NETWORK OPEN. 2022;5(7).
388. Zhang Y, Ma X, Yan G, Wu Y, Chen Y, Zhou Z, et al. Immunogenicity, durability, and safety of an mRNA and three platform-based COVID-19 vaccines as a third dose following two doses of CoronaVac in China: A randomised, double-blinded, placebo-controlled, phase 2 trial. eClinicalMedicine. 2022;54.
389. Zhou C, Qiu Y, Wang J, Zhong X, Zhu X, Huang X, et al. The safety, immunogenicity, and efficacy of heterologous boosting with a SARS-CoV-2 mRNA vaccine (SYS6006) in Chinese participants aged 18 years or more: a randomized, open-label, active-controlled phase 3 trial. 2024;13(1).
390. Zhu F, Huang S, Liu X, Chen Q, Zhuang C, Zhao H, et al. Safety and efficacy of the intranasal spray SARS-CoV-2 vaccine dNS1-RBD: a multicentre, randomised, double-blind, placebo-controlled, phase 3 trial. 2023;11(12):1075-88.
391. Zou H, Daveluy S. Linear IgA bullous dermatosis after COVID-19 vaccination. 2023;62(2):e56-e8.
392. Gee, J., Marquez, P., Su, J., Calvert, G. M., Liu, R., Myers, T., et al. (2021). First month of COVID-19 vaccine safety monitoring—United States, December 14, 2020–January 13, 2021. MMWR Morbidity and Mortality Weekly Report, 70(8), 283–288.
393. Menni, C.; Klaser, K.; May, A.; Polidori, L.; Capdevila, J.; Louca, P.; Sudre, C.H.; Nguyen, L.H.; Drew, D.A.; Merino, J.; et al. Vaccine side-effects and SARS-CoV-2 infection after vaccination in users of the COVID Symptom Study app in the UK: A prospective observational study. Lancet Infect. Dis. 2021, 21, 939–949. https://doi.org/10.1038/s41467-021-27474-4.
394. Haranaka, M., Baber, J., Ogama, Y., et al (2021). A randomized study to evaluate safety and immunogenicity of the BNT162b2 COVID-19 vaccine in healthy Japanese adults. Nature Communications, 12(1), 7105. https://doi.org/10.1038/s41467-021-27474-4.
395. Abdeen S, Abed Alah M, Al-Zaidan M, Mohamed Ibrahim MI, Abdulmajeed J, Al-Nuaimi AA, et al. Short-term side effects of BNT162b2 vaccine in primary care settings in Qatar: a retrospective study. Frontiers in Public Health. 2024;12.
396. Ain Q, Khan M, Afrah A, Akmal Z, Javed S, Nawaz A, et al. Side effects of COVID-19 vaccination in Pakistani population: A cross sectional study. Bangladesh Journal of Medical Science. 2024;23(2):355-67.
397. Al Busaidi BH, Al Riyami IM, Wazir HB, Al Zakwani IS. Analysis of COVID-19 Vaccine Adverse Drug Reactions Reported Among Sultan Qaboos University Hospital Staff. Sultan Qaboos University Medical Journal. 2024;24(2):216-20.

398. Al-Rousan N, Al-Najjar H. Evaluation of the effects of MERCK, MODERNA, PFIZER/BioNTech, and JANSSEN COVID-19 vaccines on vaccinated people: A metadata analysis. Informatics in Medicine Unlocked. 2024;49.
399. F K, D T, M M, C J, D S, K K, et al. A phase 3, randomised, observer-blinded, placebo controlled-trial evaluating the safety and immunogenicity of investigational SARS-CoV-2 mRNA vaccine CVnCoV in adult healthcare workers in Mainz (Germany). Vaccine: X. 2024;19:100512.
400. Gayed J, Diya O, Lowry F, Xu X, Bangad V, Mensa F, et al. Safety and Immunogenicity of the Monovalent Omicron XBB.1.5-Adapted BNT162b2 COVID-19 Vaccine in Individuals ≥12 Years Old: A Phase 2/3 Trial. VACCINES. 2024;12(2).
401. Hồ NT, Hughes SG, Ta VT, Phan LT, Đỗ Q, Nguyễn TV, et al. Safety, immunogenicity and efficacy of the self-amplifying mRNA ARCT-154 COVID-19 vaccine: pooled phase 1, 2, 3a and 3b randomized, controlled trials. Nat Commun. 2024;15(1).
402. Hu W-C, Chiu S-K, Yang Y-F, Singh S. COVID-19 Vaccination Reporting and Adverse Event Analysis in Taiwan. Vaccines. 2024;12(6).
403. Schmidt J, Liu Y, Skarabis H, et al. Immunogenicity, reactogenicity, and safety of a second booster with BNT162b2 or full-dose mRNA-1273: A randomized VACCELERATE trial in adults aged ≥75 years (EU-COVAT-1-AGED Part B). International Journal of Infectious Diseases. 2024;146:107161.
404. Lu Y, He C-L, Jiang G. Local and systemic reactions associated with COVID-19 vaccines: logistic regression analysis. International Journal of Dermatology and Venereology. 2024.
405. M S, I AD, A N, M A, S P, R A, et al. Safety and immunogenicity of COReNAPCIN, a SARS-CoV-2 mRNA vaccine, as a fourth heterologous booster in healthy Iranian adults: A double-blind, randomized, placebo-controlled, phase 1 clinical trial with a six-month follow-up. International immunopharmacology. 2024;134:112192.
406. Malo-Castillo J, Jiménez-Álvarez H, Ludeña-Meléndez V, Mayor Castro SS, Rodríguez S, Ishikawa-Arias P, et al. Short-Term Adverse Effects of the Fourth Dose of Vaccination against COVID-19 in Adults over 40 Years of Age. Vaccines. 2024;12(4).
407. Haverkort MR, de Melker HE, de Gier B, et al. Association between adverse events after COVID-19 vaccination and anti-SARS-CoV-2 antibody concentrations, the Netherlands, May 2021 to November 2022: a population-based prospective cohort study. Euro Surveill. 2024;29(25).
408. Lee MR, Huang CH, Chen CH, Chen CJ. Evaluation of Vaccine Strategies among Healthcare Workers during COVID-19 Omicron Outbreak in Taiwan. Vaccines. 2024;12(9).
409. N T, K S, K S, T I, T M, S T, et al. Adverse events of COVID-19 vaccination during 2021-2022 suppressed by breakfast consumption and favorable sleeping habit among Japanese university students. Vaccine: X. 2024;19:100516.
410. Hồ NT, Hughes SG, Ta VT, Phan LT, Đỗ Q, Nguyễn TV, et al. Safety, immunogenicity and efficacy of the self-amplifying mRNA ARCT-154 COVID-19 vaccine: pooled phase 1, 2, 3a and 3b randomized, controlled trials. Nature Communications. 2024;15(1).
411. P S, H W, M N, MP M, OG V, JD K, et al. mRNA COVID-19 vaccine safety among older adults from the Canadian National Vaccine Safety Network. Vaccine. 2024;42(18):3819-29.
412. Rousculp MD, Hollis K, Ziemiecki R, Odom D, Marchese AM, Montazeri M, et al. Reactogenicity Differences between Adjuvanted, Protein-Based and Messenger Ribonucleic Acid (mRNA)-Based COVID-19 Vaccines. Vaccines. 2024;12(7).
413. Saraf A, Gurjar R, Kaviraj S, Kulkarni A, Kumar D, Kulkarni R, et al. An Omicron-specific, self-amplifying mRNA booster vaccine for COVID-19: a phase 2/3 randomized trial. NATURE MEDICINE. 2024.
414. Su Y-W, Qiu Y-Z, Wang Y-H, Xu Y, Huang C-C, Zhang Q, et al. Safety and immunogenicity of heterologous boosting with a bivalent SARS-CoV-2 mRNA vaccine (XBB.1.5/BQ.1) in Chinese participants aged ≥18 years: A randomised, double-blinded, active-controlled phase 1 trial. Vaccine. 2024;42(9):2438-2447.
415. T N, K T, S H, S K-A. Comparison of adverse events following the second/third dose of BNT162b2 in a medical institute in Japan. Heliyon. 2024;10(14):e34347.
416. V M, M S, JD G, PR H. Reports of Batch-Dependent Suspected Adverse Events of the BNT162b2 mRNA COVID-19 Vaccine: Comparison of Results from Denmark and Sweden. Medicina (Kaunas, Lithuania). 2024;60(8).
417. van Dijk WJ, Prins MLM, Roukens AHE, Roozen GVT, Roestenberg M, Visser LG, et al. Coagulation and inflammatory response after intramuscular or intradermal mRNA-1273 SARS-CoV-2 vaccine: secondary analysis of a randomized trial. Research and Practice in Thrombosis and Haemostasis. 2024;8(3).

418. Yamamoto C, Kobashi Y, Kawamura T, Nishikawa Y, Saito H, Oguro F, et al. Group of longitudinal adverse event patterns after the fourth dose of COVID-19 vaccination with a latent class analysis. FRONTIERS IN PUBLIC HEALTH. 2024;12.
419. Yu X, Yang W, Li W, Wan N, Yan G, Zhou Z, et al. Safety, immunogenicity, and preliminary efficacy of a randomized clinical trial of omicron XBB.1.5-containing bivalent mRNA vaccine. hLife. 2024;2(3):113-25.
420. Li LL, Zheng C, La J, Do V, Nhan, Monach PA, Strymish JM, et al. Impact of prior SARS-CoV-2 infection on incidence of hospitalization and adverse events following mRNA SARS-CoV-2 vaccination: A nationwide, retrospective cohort study. VACCINE. 2022;40(8):1082-9.
421. Urdaneta V, Esposito DB, Dharia P, Moraga MS, Anteyi K, Oduyebo-Omotosho T, Rossi M, Burton P, Vega JM, Dawson R, Straus W. Global Safety Assessment of Adverse Events of Special Interest Following 2 Years of Use and 772 Million Administered Doses of mRNA-1273. Open Forum Infect Dis. 2024 Feb 2;11(3):ofae067. doi: 10.1093/ofid/ofae067. PMID: 38500575; PMCID: PMC10946654.
422. Lambo J, Keli S, Kaplan SK, Njideaka-Kevin T, Arja SB, Khedir Omer Altahir A, Olonade I, Kumar R. The descriptive epidemiology of adverse events following two doses of mRNA COVID-19 vaccination in Curaçao, the Caribbean. Infect Dis (Lond). 2025 Feb;57(2):137-149. doi: 10.1080/23744235.2024.2399108. Epub 2024 Sep 3. PMID: 39226235.
423. Abdul-Nabi ZN, Mohamed-Jawad NK, Fareed NY, Neamah NF, Shari FH. Adverse Effects Post COVID-19 Vaccination and its Association with Age, Gender and Comorbid Disease in Basrah City Southern of Iraq. 2024;19(2):248-54.
424. Sabati K, Kovačić B, Pavičić M, Kuliš I, Skvrce NM, Tomić S. Suspected adverse reactions to COVID-19 vaccines reported to the Agency for Medicinal Products and Medical Devices. Praćenje sumnji nuspojava na cjepiva protiv bolesti COVID-19 prijavljenih Agenciji za lijekove i medicinske proizvode. 2022;78(1):29-44.
425. Randomised Phase II Study to Assess the Immunogenicity and Safety of Heterologous SARS-CoV-2 Vaccine Schedules (rAd26-rAd5, rAd26-rAd26, rAd26-ChAdOx1 and rAd26-mRNA-1273)2021 2021.
426. Immunogenicity and Adverse Events Following Immunization (AEFI) With Alternate Schedules of COVID-19 Vaccines in Canada: is "Mix and Match" of the Second Dose (MOSAIC-1;CT24a) and Additional Doses (MOSAIC-2 and MOSAIC-3;CT24b and CT24c) Safe and Immunogenic?2021 2021.
427. A Phase 1/2 Study of Delayed Heterologous SARS-CoV-2 Vaccine Dosing (Boost) After Receipt of EUA Vaccines2021 2021.
428. Phase 1, Open-Label, Randomized Study of the Safety and Immunogenicity of a SARS-CoV-2 Variant Vaccine (mRNA-1273.351) in Naïve and Previously Vaccinated Adults2021 2021.
429. A Phase 1a/1b, Randomized, Observer-Blind, Placebo-Controlled Study to Evaluate the Safety, Tolerability, and Immunogenicity of the COVID-19 (SARS-CoV-2) Vaccine Candidates VBI-2902a and VBI-2905a in Healthy Adults2021 2021.
430. A Phase 2 Randomized, Open-Label, Parallel Group Study to Assess the Immunogenicity, Safety, and Tolerability of Moderna mRNA-1273 Vaccine Administered With Casirivimab+Imdevimab in Healthy Adult Volunteers2021 2021-4-19.
431. Phase 2 Clinical Trial to Optimize Immune Coverage of SARS-CoV-2 Existing and Emerging Variants2022 2022.
432. Randomized, Observer-Blind, Active-Controlled, Clinical Trial to Assess the Immunogenicity of an Investigational mRNA-1273.815 COVID-19 Vaccine in Previously Vaccinated Adults2024 2024.
433. A Y, N W, PJ S, EN R, H K, JR S, et al. Sex and gender differences in adverse events following influenza and COVID-19 vaccination. Biology of sex differences. 2024;15(1):50.
434. Abdel Aziz HS, Hamed RMR, Abd El-Ghani SE, Hassanen HAM, Abdelmageed RA, Soliman SB, et al. Assessing COVID-19 Risks among Egyptian Healthcare Workers: Incidence, Reinfection, and Vaccination Insights: A Retrospective Study. 2025;34(2):345-59.
435. Acuti Martellucci C, Rosso A, Zauli E, Bianconi A, Fiore M, Soldato G, et al. The Effectiveness of Four Quadrivalent, Inactivated Influenza Vaccines Administered Alone or in Combination with Pneumococcal and/or SARS-CoV-2 Vaccines: A Population-Wide Cohort Study. 2025;13(3).
436. AD A, RR A, SS S, N A-S, HA N, H L, et al. COVID-19 Vaccines Breakthrough Infections and Adverse Effects Reported by the Birzeit University Community in Palestine. International journal of general medicine. 2024;17:3349-60.
437. Ann Costa Clemens S, Weckx L, Milan EP, Smolenov I, Clemens R. Interchangeability of different COVID-19 vaccine platforms as booster doses: A phase 3 study mimicking real-world practice. 2024;42(19):3989-98.

438. B Z, Y Z, J K, L Z. BAYESIAN LEARNING OF COVID-19 VACCINE SAFETY WHILE INCORPORATING ADVERSE EVENTS ONTOLOGY. The annals of applied statistics. 2023;17(4):2887-902.
439. Barnay M, Foubert-Samier A, Violleau M-H, Campana-Salort E, Cintas P, Laforêt P, et al. The safety of COVID-19 vaccines in a large French series of patients with neuromuscular conditions and the impacts of vaccination on their daily lives. 2025.
440. Bennett C, Rivers EJ, Woo W, Bloch M, Cheung K, Griffin P, et al. Immunogenicity and Safety of Heterologous Omicron BA.1 and Bivalent SARS-CoV-2 Recombinant Spike Protein Booster Vaccines: A Phase 3 Randomized Clinical Trial. J Infect Dis. 2024;230(1):e4-e16.
441. C L-M, M T, B A-C, NH W, L C-C, AA C-D, et al. Phase II study on the safety and immunogenicity of single-dose intramuscular or intranasal administration of the AVX/COVID-12 "Patria" recombinant Newcastle disease virus vaccine as a heterologous booster against COVID-19 in Mexico. Netherlands2025 2025-1-1. 126511 p.
442. Dutta S, Kaur RJ, Bhardwaj P, Sharma P, Ambwani S, Islam S, et al. Adverse events reported from the COVID-19 vaccines: A descriptive study based on the WHO database (VigiBase®). 2021;11(8):1-9.
443. Fierro C, Sanchez-Crespo N, Makrinos D, Zhang W, Sun Y, Rohilla P, et al. Shared clinical and immunologic features of mRNA vaccines: preliminary results from a comparative clinical study. Front Immunol. 2025;16.
444. Garrett N, Reddy T, Yende-Zuma N, Takalani A, Anteyi K, Woeber K, et al. Heterologous mRNA-1273 Boost After Ad26.CoV2.S Prime in Health Workers in South Africa: SHERPA Trial. Top Antiviral Med. 2024;32(1):92-3.
445. Hannawi S, Wu X-H, Villalobos RE, Burhan E, Lallaine Borra MD, Gupta RK, et al. Efficacy, immunogenicity, and safety of a monovalent mRNA vaccine, ABO1020, in adults: A randomized, double-blind, placebo-controlled, phase 3 trial. Med. 2024;5(10):1282-92.e3.
446. Hirano H. Anti-spike protein IgG antibody responses up to 3 months after the third dose of the BNT162b2 mRNA vaccine in medical care workers. 2024;25(3):140-5.
447. J S-P, G C, J B, MM M, MÁ M, N E, et al. Association between Reactogenicity and Immunogenicity in a Vaccinated Cohort with Two mRNA SARS-CoV-2 Vaccines at a High-Complexity Reference Hospital: A Post Hoc Analysis on Immunology Aspects of a Prospective Cohort Study. Vaccines. 2024;12(6).
448. Kazaal MA, Jaythoom RA, Mahdi ND, Mohammed DY. Evaluating the Side Effects of COVID-19 Vaccines Available in Al-Diwaniyah Province. 2024;21(3):521-6.
449. Kim HJ, Suh JH, Kim M-H, Choi MG, Chun EM. Broad-Spectrum Adverse Events of Special Interests Based on Immune Response Following COVID-19 Vaccination: A Large-Scale Population-Based Cohort Study. 2025;14(5).
450. L R-P, MM S, CK O, CE D, E vP, D W, et al. Comparison of Adverse Events in Pregnant Persons Receiving COVID-19 and Influenza Vaccines: A Disproportionality Analysis Using Combined Data from US VAERS and EudraVigilance Spontaneous Report Databases. Drug safety. 2025.
451. Lee IT, Cosgrove CA, Moore P, Bula M, Kalra P, Dargan P, et al. Three-Month Safety and Immunogenicity of Bivalent SARS-CoV-2 Omicron-Containing Booster Vaccines: Interim Results From a Phase 3, Randomized, Observer-Blind, Active-Controlled Trial. Open Forum Infect Dis. 2023;10:S1009-S10.
452. Lu Y-A, Huang F-Y, Chi H, Lin C-Y, Chiu N-C. Preliminary Report of Nationwide COVID-19 Vaccine Compensation in Taiwan. 2024;12(13).
453. MAF P, M S, M AS, Y N, S K. COVID-19 vaccination and major cardiovascular and haematological adverse events in Abu Dhabi: retrospective cohort study. Nature communications. 2024;15(1):5490.
454. Miyaji KT, Ibrahim KY, Infante V, Moreira RM, Dos Santos CF, de Cássia Belizário J, et al. Safety of two-dose schedule of COVID-19 adsorbed inactivated vaccine (CoronaVac; Sinovac/Butantan) and heterologous additional doses of mRNA BNT162b2 (Pfizer/ BioNTech) in immunocompromised and immunocompetent individuals. 2025;67.
455. Moreira Puga MA, Dias de Oliveira R, Vieira da Silva P, Charu V, Hedlin H, Lu D, et al. Immunogenicity and reactogenicity of fractional vs. full booster doses of COVID-19 vaccines: a non-inferiority, randomised, double-blind, phase IV clinical trial in Brazil. 2025;44.
456. Mukenge EK, Sumbu BM-M, Nkodila AN, Muwonga JM, Makulo J-RR, Ngole MZ, et al. Incidence of dynamic seroconversion in subjects received the first dose of the SARS-COV-2 vaccine (AstraZeneca, Moderna and Pfizer) in Kinshasa, Democratic Republic of Congo: prospective cohort study. 2025;25(1).
457. Nagare A, Martin E, Koen A, Mitha E, Ahmed K, Dhar M, et al. Durable immune response induced by self-amplifying mRNA (samRNA) SARS-CoV-2 vaccine candidates against variants of concern (VOC) in HIV-

negative and people living with HIV (PLWH) populations in South Africa, irrespective of prior SARS-CoV-2 vaccination. Open Forum Infect Dis. 2025;12:S193-S5.
458. Neutel JM, Erdem R, Jiang Q, Cannon K, Stacey H, Newton R, et al. Safety and Immunogenicity of Concomitant Administration and Combined Administration of Bivalent BNT162b2 COVID-19 Vaccine and Bivalent RSVpreF Respiratory Syncytial Virus Vaccine with or Without Quadrivalent Influenza Vaccine in Adults ≥ 65 Years of Age. 2025;13(2).
459. NT H, I S, L TLT, VT N, VT T, TV N, et al. Safety profile of self-amplifying mRNA SARS-CoV-2 vaccine ARCT-154 in adults: a pooled phase 1/2/3 randomized clinical study. England2025 2025-12. 299-312 p.
460. P S, N S, JK S, DA C, RP S, KE T. The Risk of Optic Neuritis following mRNA Coronavirus Disease 2019 Vaccination Compared to Coronavirus Disease 2019 Infection and Other Vaccinations. United States2024 2024-9. 1076-82 p.
461. Park YH, Kim MH, Choi MG, Chun EM. Correlation between COVID-19 vaccination and inflammatory musculoskeletal disorders. medRxiv. 2023:2023.11.14.23298544.
462. Putri ND, Zhafira AS, Wicaksana P, Sinto R, Hanafi G, Wiyono L, et al. Immunogenicity and Safety of Half-Dose Heterologous mRNA-1273 Booster Vaccination for Adults Primed with the CoronaVac® and ChAdOx1-S Vaccines for SARS-CoV-2. Vaccines. 2024;12(4).
463. R F-d-S, MF L, AM P, M M, JJ P, I R-V. Network analysis of adverse event patterns following immunization with mRNA COVID-19 vaccines: real-world data from the European pharmacovigilance database EudraVigilance. Frontiers in medicine. 2025;12:1501921.
464. Roozen GVT, Prins MLM, Prins C, Janse JJ, de Gruyter HLM, Pothast CR, et al. Intradermal delivery of the third dose of the mRNA-1273 SARS-CoV-2 vaccine: safety and immunogenicity of a fractional booster dose. 2024;30(7):930-6.
465. Rudman Spergel AK, Ananworanich J, Guo R, Deng W, Carmona L, Schaefers K, et al. mRNA-based seasonal influenza and SARS-CoV-2 multicomponent vaccine in healthy adults: a phase 1/2 trial. Nat Med. 2025;31(5):1484-93.
466. Runge M, Karimian Z, Kheirandish M, Borghi G, Wodniak N, Fahmy K, et al. COVID-19 Vaccine Effectiveness Studies against Symptomatic and Severe Outcomes during the Omicron Period in Four Countries in the Eastern Mediterranean Region. 2024;12(8).
467. Saavedra RDC, Paixao ES, Ichihara MYT, Costa MDCN, Carvalho-Sauer R, de Castro CT, et al. Prevalence of Adverse Events Reported Following the First Dose of COVID-19 Vaccines in Bahia State, Brazil, from 2021 to 2022. 2025;13(2).
468. Saiag E, Gamzu R, Padova H, Paran Y, Goldiner I, Cohen N, et al. Antibody Response After a Fifth Dose (Third Booster) of BNT162b2 mRNA COVID-19 Vaccine in Healthcare Workers. 2024;13(21).
469. Saraf A, Gurjar R, Kaviraj S, Kulkarni A, Kumar D, Kulkarni R, et al. An Omicron-specific, self-amplifying mRNA booster vaccine for COVID-19: a phase 2/3 randomized trial. Nat Med. 2024;30(5):1363-72.
470. Sauserienė J, Serapinas D, Liseckienė I, Budrevičiūtė A, Vaičiūnienė R, Gradauskienė B, et al. The Impact of the Emotional Disposition of Healthcare Workers on the Expression of Adverse Events after Primary Vaccination against SARS-CoV-2. 2024;60(3).
471. Silva RMA, Cabral CM, Percio J, da Nóbrega MEB, Kobayashi CD, Martins AF, et al. Pharmacovigilance of COVID-19 Vaccines in Brazil: Actions Taken, Lessons Learned, and Next Steps. 2025;49.
472. Sow SO, Tapia MD, Haidara FC, Diallo F, Han X, Chen J, et al. Safety, reactogenicity, and immunogenicity of ZR-202-CoV and ZR-202a-CoV recombinant vaccines compared with Comirnaty®: A randomized, observer-blind, controlled, phase 1 study. 2024;148.
473. Tshipata RM, Diyoka CK, Nwakama CN, Mwanvua LN, Mpoji FN, Mubalamata CM, et al. Vaccination Coverage Covid-19 and Self-Reported Adverse Post-Vaccination Events in Mbuji-Mayi, DR Congo. 2025;18:1687-97.
474. University AK, Foundation OC, University S. Randomized Controlled Trial to Assess the Immunogenicity and Safety of Full Versus Fractional Dose of Pfizer/BioNTech, AstraZeneca, and Sinovac COVID-19 Vaccines Given as a Booster Dose at Least 6 Months After Primary Vaccination Series or PCR-confirmed Infection With SARS-CoV-2 in Healthy Adults2022 2022-4-20.
475. Uriel A, Garbes P, Nagare A, Kuan JC, Palmer C, Bae K, et al. Preliminary results of durable immune response induced by a self-amplifying mRNA (samRNA) SARS-CoV-2 vaccine candidate, GRT-R910, in adults previously vaccinated with mRNA or AZD1222 primary series. Open Forum Infect Dis. 2023;10:S1001.
476. Ventura-Enríquez Y, Cortina-De la Rosa E, Díaz-Padilla E, Murrieta S, Segundo-Martínez S, Fernández-Sánchez V, et al. Immunogenicity of Two Doses of BNT162b2 mRNA COVID-19 Vaccine with a ChAdOx1-S Booster Dose among Navy Personnel in Mexico. 2024;16(4).

477. W N, J R, G N, M N, R B-D, L D-S. Serious adverse drug reactions associated with anti-SARS-CoV-2 vaccines and their reporting trends in the EudraVigilance database. Scientific reports. 2025;15(1):18582.
478. XQ L, YY Q, T H, LL H, MX Z, ZF W, et al. Efficacy, immunogenicity, and safety of heterologous boosting with a novel chimera Chinese mRNA (RQ3013) SARS-CoV-2 vaccine: A randomized, double-blind, active-controlled trial. United States2025 2025-12. 2502250 p.
479. Y G, Y C, J H, C Z, W Z, L Q, et al. Safety and immunogenicity of a modified COVID-19 mRNA vaccine, SYS6006, as a fourth-dose booster following three doses of inactivated vaccines in healthy adults: an open-labeled Phase 1 trial. Life metabolism. 2023;2(3):load019.
480. Y O, Y K, I O, M O, N I, Y I, et al. Immunogenicity of a booster dose of a bivalent (Asp614Gly and omicron BA.4/5 variant) self-amplifying mRNA SARS-CoV-2 booster vaccine versus the BNT162b2 omicron BA.4/5 mRNA vaccine: a randomised phase 3 trial. United States2025 2025-3. 290-300 p.
481. Yadav G, Dandu H, Malhotra HS, Jain A, Radera S, Agrawal V, et al. COVID-19 vaccination and utility of booster dose: A community-based cross-sectional study. 2025;60.
482. Yoon D, Jung K, Kim JH, Ko HY, Yoon B-A, Shin J-Y. Risk for Facial Palsy after COVID-19 Vaccination, South Korea, 2021–2022. 2024;30(11):2313-22.
483. Zidan AA, Jad AY, Zakaria NH, El-Hariri HM, El-Setouhy M. Assessment of the factors affecting the clinical outcomes of infection and safety of vaccines against SARS-CoV-2 among Egyptian patients. 2025;25(1).
484. A B, L L, S S, CR M, J B, ES A, et al. Comprehensive humoral and cellular immune responses to COVID-19 vaccination in adults with cancer. Vaccine. 2025;46:126547.
485. AL F, JR A, B E, F P, D S, U S, et al. Safety and Immunogenicity of the mRNA-1273 Coronavirus Disease 2019 Vaccine in Solid Organ Transplant Recipients. United States2024 2024-9-23. e591-e600 p.
486. Al-Battat RA, Al-Aish ST, Al Sakini AS, Allawi MM. Systemic and cutaneous side effects of COVID-19 vaccines in Iraq, A cross-sectional study. 2024;27.
487. B P, HA L, Y K, CH K, H P, S J, et al. Active Surveillance for Safety Monitoring of XBB.1.5-Containing COVID-19 mRNA Vaccines in Korea. Journal of Korean medical science. 2024;39(43):e309.
488. Bannister W, Raben D, Valentiner-Branth P, Tolstrup M, Larsen L, Tarp B, et al. Association of Self-reported Systemic Reactions Following SARS-CoV-2 Vaccination With Immunological Response in the Danish National Cohort Study of Effectiveness and Safety of SARS-CoV-2 Vaccines (ENFORCE). OPEN FORUM INFECTIOUS DISEASES. 2023;10(6).
489. BJ E, C S, MGD I, P B, A P, M B, et al. Safety and immunogenicity of a modified mRNA-lipid nanoparticle vaccine candidate against COVID-19: Results from a phase 1, dose-escalation study. United States2024 2024-12-31. 2408863 p.
490. C M, M D, KL F, M P, H M, MJ E, et al. The Platform Trial In COVID-19 Priming and BOOsting (PICOBOO): The immunogenicity, reactogenicity, and safety of different COVID-19 vaccinations administered as a second booster (fourth dose) in AZD1222 primed individuals aged 50-<70 years old. England2024 2024-12. 106286 p.
491. C Y, Y K, T K, Y N, H S, F O, et al. Group of longitudinal adverse event patterns after the fourth dose of COVID-19 vaccination with a latent class analysis. Frontiers in public health. 2024;12:1406315.
492. Chen G, Yu X, Luo L, Zhang F, Dai X, Li N, et al. Phase I study of a non-S2P SARS-CoV-2 mRNA vaccine LVRNA009 in Chinese adults. VACCINE. 2023;41(48):7297-306.
493. Chu L, Vrbicky K, Montefiori D, Huang W, Nestorova B, Chang Y, et al. Immune response to SARS-CoV-2 after a booster of mRNA-1273: an open-label phase 2 trial. NATURE MEDICINE. 2022;28(5):1042-+.
494. DM D, SK S, E K-B, E N, K A-A, NT M, et al. Safety of mRNA COVID-19 vaccines among persons 15- years and above in Ghana: A cohort event monitoring study. Vaccine. 2024;42(26):126460.
495. Dorajoo S, Tan H, Teo C, Neo J, Koon Y, Ng J, et al. Nationwide safety surveillance of COVID-19 mRNA vaccines following primary series and first booster vaccination in Singapore. VACCINE: X. 2023;15.
496. Dutcher EG, Epel ES, Mason AE, Hecht FM, Robinson JE, Drury SS, et al. COVID-19 Vaccine Side Effects and Long-Term Neutralizing Antibody Response A Prospective Cohort Study. 2024;177(7):892-900.
497. EB W, EP S, KR T, W R, KR B, J D, et al. Safety of Simultaneous vs Sequential mRNA COVID-19 and Inactivated Influenza Vaccines: A Randomized Clinical Trial. JAMA network open. 2024;7(11):e2443166.
498. G M, S C, S C, E F, CYJ F, E L, et al. COVID-19 vaccine reactogenicity among participants enrolled in the GENCOV study. Vaccine. 2024;42(11):2733-9.
499. Gui Y, Li X, Li J, Shen M, Zhang M, Cao Y, et al. Safety and immunogenicity of a modified COVID-19 mRNA vaccine, SW-BIC-213, as a heterologous booster in healthy adults: an open-labeled, two-centered and multi-arm randomised, phase 1 trial. EBIOMEDICINE. 2023;91.

500. H Z, H Z, Y P, Y S, X Y, W W, et al. Efficacy, safety, and immunogenicity of SARS-CoV-2 mRNA vaccine (Omicron BA.5) LVRNA012: a randomized, double-blind, placebo-controlled phase 3 trial. Switzerland2024 2024. 1407826 p.
501. HJ C, C P, JH M, P E, L B, D C, et al. Nuvaxovid NVX-CoV2373 vaccine safety profile: real-world data evidence after 100,000 doses, Australia, 2022 to 2023. Euro surveillance : bulletin Europeen sur les maladies transmissibles = European communicable disease bulletin. 2024;29(50).
502. HS B, SU L, YD W, TW C, SH L, YD K, et al. Nationwide Cohort observational study on the safety and efficacy of COVID-19 vaccination in patients with Moyamoya disease. Scientific reports. 2024;14(1):24400.
503. Huang S, Huang Y, Liu W, Pan S, Sun H, Lien C, et al. Immunogenicity and safety of heterologous mRNA-1273/MVC-COV1901 vaccination versus homologous mRNA1273 vaccination: A randomized, double-blind controlled study. JOURNAL OF THE FORMOSAN MEDICAL ASSOCIATION. 2023;122(11):1165-73.
504. Hui A, Li J, Zhu L, Tang R, Ye H, Lin M, et al. Immunogenicity and safety of BNT162b2 mRNA vaccine in Chinese adults: A phase 2 randomised clinical trial. LANCET REGIONAL HEALTH-WESTERN PACIFIC. 2022;29.
505. J G, JF C, DC H, AK S, L W, R D, et al. Safety and immunogenicity of mRNA-1345 RSV vaccine coadministered with an influenza or COVID-19 vaccine in adults aged 50 years or older: an observer-blinded, placebo-controlled, randomised, phase 3 trial. The Lancet Infectious diseases. 2024.
506. Jorda A, Prager M, Pracher L, Haselwanter P, Jackwerth M, al Jalali V, et al. Immunogenicity, safety, and reactogenicity of concomitant administration of the novavax vaccine against Omicron XBB.1.5 (NVX-CoV2601) and a 20-valent pneumococcal conjugate vaccine in adults aged ≥60 years: A randomised, double-blind, placebo-controlled, non-inferiority trial. 2025;90(2).
507. Jubishi D, Okamoto K, Hamada K, Ishii T, Hashimoto H, Shinohara T, et al. The association between adverse reactions and immune response against SARS-CoV-2 spike protein after vaccination with BNT162b2 among healthcare workers in a single healthcare system: a prospective observational cohort study. HUMAN VACCINES & IMMUNOTHERAPEUTICS. 2022;18(5).
508. K A, K K, RS M, A K, JS P, R K, et al. Immunogenicity and safety of a monovalent omicron XBB.1.5 SARS-CoV-2 recombinant spike protein vaccine as a heterologous booster dose in US adults: interim analysis of a single-arm phase 2/3 study. The Lancet Infectious diseases. 2025.
509. K K, K M, T M, K S, S S, RP S, et al. Immunogenicity and safety of a single booster dose of NVX-CoV2373 (TAK-019) in healthy Japanese adults who had previously received a primary series of COVID-19 mRNA vaccine: Primary analysis report of a phase 3 open-label trial. Vaccine. 2023;41(25):3763-71.
510. Karam R, Iskandar K, Watfa M, Zeitoun A. Serious adverse events following immunization with COVID-19 vaccines in Lebanon: a retrospective analysis of the National Pharmacovigilance Database. 2024;24(1).
511. Klingel H, Krüttgen A, Imöhl M, Kleines M. Humoral immune response to SARS-CoV-2 mRNA vaccines is associated with choice of vaccine and systemic adverse reactions. CLINICAL AND EXPERIMENTAL VACCINE RESEARCH. 2023;12(1):60-9.
512. LAR O, AS C, TA dJ, MJS B, EA T, RO R. Distinct Adverse Reactions to mRNA, Inactivated Virus, and Adenovirus Vector COVID-19 Vaccines: Insights from a Cohort Study on Atopic and Non-Atopic Subjects in Brazil. Vaccines. 2024;12(4).
513. Lee I, Cosgrove C, Moore P, Bethune C, Nally R, Bula M, et al. Omicron BA.1-containing mRNA-1273 boosters compared with the original COVID-19 vaccine in the UK: a randomised, observer-blind, active-controlled trial. LANCET INFECTIOUS DISEASES. 2023;23(9):1007-19.
514. Liberata S, Gaetano L, Vera P, Giuseppe C, Simona P, Giacomo O, et al. Oropharyngeal Adverse Events to Drugs and Vaccines: Pharmacovigilance Data From Italy (2019–2021). 2024.
515. Liu X, Sun Z, Wang Z, Chen J, Wu Q, Zheng Y, et al. Safety, immunogenicity, and efficacy of an mRNA COVID-19 vaccine (RQ3013) given as the fourth booster following three doses of inactivated vaccines: a double-blinded, randomised, controlled, phase 3b trial. ECLINICALMEDICINE. 2023;64.
516. López-Contreras JE, Paredes-Casillas P, Morales-Romero J, Castillo-Vélez FE, Lona-Reyes JC, Bedolla-Barajas M. Incidence and factors associated with early and late adverse reactions after the first dose of Pfizer-BioNTech vaccine among healthcare workers. 2023;91(1):34-40.
517. M I, S Y, Y S, T S, K T, E M, et al. COVID-19 mRNA booster vaccination induces robust antibody responses but few adverse events among SARS-CoV-2 naïve nursing home residents. Scientific reports. 2024;14(1):23295.

518. M S, I AD, A N, M A, S P, R A, et al. Safety and immunogenicity of COReNAPCIN, a SARS-CoV-2 mRNA vaccine, as a fourth heterologous booster in healthy Iranian adults: A double-blind, randomized, placebo-controlled, phase 1 clinical trial with a six-month follow-up. Netherlands2024 2024-6-15. 112192 p.
519. M Y, G Q, Y L, N D, E S, D Y, et al. Comparison of physiological and clinical reactions to COVID-19 and influenza vaccination. Communications medicine. 2024;4(1):169.
520. Marziali M, Pugliese P, Losardo AA, Ribersani M, Anastasi E, Angeloni A, et al. Efficacy and safety of BNT162b2 mRNA vaccine in a cohort of 90 transfusion dependent thalassemia patients. 2024;34(3):238-40.
521. Miyashita Y, Yoshida T, Takagi Y, Tsukamoto H, Takashima K, Kouwaki T, et al. Circulating extracellular vesicle microRNAs associated with adverse reactions, proinflammatory cytokine, and antibody production after COVID-19 vaccination. NPJ VACCINES. 2022;7(1).
522. MJ LF, S N, A C, JM E-S, MJ F, E A-A, et al. Omicron XBB.1.16-Adapted Vaccine for COVID-19: Interim Immunogenicity and Safety Clinical Trial Results. Vaccines. 2024;12(8).
523. N G, T R, N Y-Z, A T, K W, A B, et al. Safety, effectiveness and immunogenicity of heterologous mRNA-1273 boost after prime with Ad26.COV2.S among healthcare workers in South Africa: The single-arm, open-label, phase 3 SHERPA study. PLOS global public health. 2024;4(12):e0003260.
524. Naficy A, Kuxhausen A, Seifert H, Hastie A, Leav B, Miller J, et al. No immunological interference or concerns about safety when seasonal quadrivalent influenza vaccine is co-administered with a COVID-19 mRNA-1273 booster vaccine in adults: A randomized trial. HUMAN VACCINES & IMMUNOTHERAPEUTICS. 2024;20(1).
525. Niyomnaitham S, Chokephaibulkit K, Pheerapanyawaranun C, Toh ZQ, Licciardi PV, Satayasanskul A, et al. Immunogenicity of BNT162b2 as a first booster after a ChAdOx1 primary series in a Thai geriatric population living with frailty. 2024;28(8).
526. NT H, SG H, R S, VT T, TV N, AT VP, et al. A randomized trial comparing safety, immunogenicity and efficacy of self-amplifying mRNA and adenovirus-vector COVID-19 vaccines. NPJ vaccines. 2024;9(1):233.
527. OO W, D V, G Q, C L, SD K, SK K, et al. Innate Responses to the Former COVID-19 Vaccine Candidate CVnCoV and Their Relation to Reactogenicity and Adaptive Immunogenicity. Vaccines. 2024;12(4).
528. Park H, Lim E, Jun S, Lee H, Lee HA, Park H, et al. Reported Adverse Events and Associated Factors in Korean Coronavirus Disease 2019 Vaccinations. 2024;39(42).
529. Pasternak G, Pieniawska-Smiech K, Walkowiak M, Sado J, Pytel A, Jasinska P, et al. Before and After: Attitude and Adverse Effects Induced by the First and Second Doses of mRNA BNT162b2 Vaccine among Healthcare Professionals in the First Weeks after Their Introduction in Poland. VACCINES. 2023;11(5).
530. R R, E T, M D, L D, C G, LK L, et al. Short-Term Active Safety Surveillance of the Spikevax and Nuvaxovid Priming Doses in Australia. Vaccines. 2024;12(9).
531. Rasheed WS, Sarkees AN. Immediate Adverse Reaction and SARS-CoV-2 Anti-Spike Receptor Binding Domain IgG of COVID-19 Vaccines among Health Staffs. 2024;18.
532. RS T, U A, M J, R B, C R, SN A, et al. Adverse events after first and second doses of COVID-19 vaccination in England: a national vaccine surveillance platform self-controlled case series study. Journal of the Royal Society of Medicine. 2024;117(4):134-48.
533. S I, N T, S K, Y K, T N, S H, et al. Safety comparison between Pfizer BNT162b2, Moderna mRNA-1273, and AstraZeneca AZD1222 in a Nationwide prospective cohort survey at the beginning of the severe acute respiratory syndrome coronavirus 2 vaccination in Japan. Vaccine. 2025;49:126754.
534. S K, J C, N S, L W, R A, P V, et al. Safety and antibody responses of Omicron BA.4/5 bivalent booster vaccine among hybrid immunity with diverse vaccination histories: A cohort study. Vaccine: X. 2024;20:100538.
535. Saez-Llorens X, Lanata C, Aranguren E, Celis C, Cornejo R, DeAntonio R, et al. Safety and immunogenicity of mRNA-LNP COVID-19 vaccine CVnCoV in Latin American adults: A phase 2 randomized study. VACCINE: X. 2022;11.
536. Santi T, Sungono V, Kamarga L, De Samakto B, Hidayat F, Hidayat F, et al. Heterologous prime-boost with the mRNA-1273 vaccine among CoronaVac-vaccinated healthcare workers in Indonesia. CLINICAL AND EXPERIMENTAL VACCINE RESEARCH. 2022;11(2):209-16.
537. Sheng W, Chang S, Lin P, Hsieh M, Chang H, Cheng C, et al. Immune response and safety of heterologous ChAdOx1-nCoV-19/mRNA-1273 vaccination compared with homologous ChAdOx1-nCoV-19 or homologous mRNA-1273 vaccination. JOURNAL OF THE FORMOSAN MEDICAL ASSOCIATION. 2022;121(4):766-77.

538. Sheng W, Ieong S, Lin P, Hsieh M, Yang H, Pan C, et al. Immunogenicity and safety of third-dose mRNA COVID-19 vaccines in healthy adults previously vaccinated with two doses of the ChAdOx1 vaccine. JOURNAL OF THE FORMOSAN MEDICAL ASSOCIATION. 2023;122(2):121-31.
539. Shoaibi A, Lloyd P, Wong H, Clarke T, Chillarige Y, Do R, et al. Evaluation of potential adverse events following COVID-19 mRNA vaccination among adults aged 65 years and older: Two self-controlled studies in the US. VACCINE. 2023;41(32):4666-78.
540. SK C, S K, M K, Y H, TE K, Y L, et al. Safety of the bivalent COVID-19 mRNA booster vaccination among persons aged over 18 years in the Republic of Korea. Osong public health and research perspectives. 2024;15(6):542-9.
541. Sonoyama T, Kamitani A, Shibata R, Seki N, Omoto S, Igarashi K, et al. Safety and immunogenicity of a booster dose of S-268019-b: Interim findings of a Phase 3, open-label clinical study in Japan. VACCINE: X. 2023;15.
542. Steenackers K, Hanning N, Bruckers L, Desombere I, Marchant A, Ariën KK, et al. Humoral immune response against SARS-CoV-2 after adapted COVID-19 vaccine schedules in healthy adults: The IMCOVAS randomized clinical trial. 2024;42(25).
543. Strobel S, Machiraju D, Kälber K, Hassel J. Immune-related adverse events of COVID-19 vaccination in skin cancer patients receiving immune-checkpoint inhibitor treatment. CANCER IMMUNOLOGY IMMUNOTHERAPY. 2022;71(8):2051-6.
544. Sun JW, Dodge LE, Kim EJ, Zhou L, Mather S, Goebe H, et al. Risk of adverse events after Omicron XBB-adapted BNT162b2 COVID-19 vaccination in the United States. 2025;45.
545. T N, K T, S H, S K-A. Comparison of adverse events following the second/third dose of BNT162b2 in a medical institute in Japan. Heliyon. 2024;10(14):e34347.
546. Tan-Lim C, Gonzales M, Dans L, Cordero C, Alejandria M, dela Paz E, et al. Reinfection rates, change in antibody titers and adverse events after COVID-19 vaccination among patients previously infected with COVID-19 in Metro Manila, Philippines: a secondary analysis of a completed cohort study. BMC INFECTIOUS DISEASES. 2023;23(1).
547. Thiem VD, Anh DD, Ha VH, Van Thom N, Thang TC, Mateus J, et al. Safety and immunogenicity of an inactivated recombinant Newcastle disease virus vaccine expressing SARS-CoV-2 spike: A randomised, comparator-controlled, phase 2 trial. 2025;44.
548. V M, M S, JD G, PR H. Reports of Batch-Dependent Suspected Adverse Events of the BNT162b2 mRNA COVID-19 Vaccine: Comparison of Results from Denmark and Sweden. Switzerland2024 2024-8-19.
549. Voulgaridi I, Sarrou S, Dadouli A, Peristeri A, Nasika A, Onoufriadis I, et al. Intensity of Humoral Immune Responses, Adverse Reactions, and Post-Vaccination Morbidity after Adenovirus Vector-Based and mRNA Anti-COVID-19 Vaccines. VACCINES. 2022;10(8).
550. Wan EYF, Wang B, Lee AL, Zhou J, Chui CSL, Lai FTT, et al. Comparative effectiveness and safety of BNT162b2 and CoronaVac in Hong Kong: A target trial emulation. 2024;146.
551. Wong H, Tworkoski E, Zhou C, Hu M, Thompson D, Lufkin B, et al. Surveillance of COVID-19 vaccine safety among elderly persons aged 65 years and older. VACCINE. 2023;41(2):532-9.
552. Wu J, Li J, Liu J, Wang H, Zhou G, Li J, et al. Safety, immunogenicity, and efficacy of the mRNA vaccine CS-2034 as a heterologous booster versus homologous booster with BBIBP-CorV in adults aged ≥18 years: a randomised, double-blind, phase 2b trial. LANCET INFECTIOUS DISEASES. 2023;23(9):1020-30.
553. Y M, M K, G F, K K, H U, D S, et al. Reported Adverse Events Following SARS-CoV-2 Vaccinations in the Canadian Province of Alberta and Associated Risk Factors: A Retrospective Cohort Study. Vaccines. 2024;12(12).
554. Yoshida M, Kobashi Y, Kawamura T, Shimazu Y, Nishikawa Y, Omata F, et al. Association of systemic adverse reaction patterns with long-term dynamics of humoral and cellular immunity after coronavirus disease 2019 third vaccination. SCIENTIFIC REPORTS. 2023;13(1).
555. Yu X, Yang W, Li W, Wan N, Yan G, Zhou Z, et al. Safety, immunogenicity, and preliminary efficacy of a randomized clinical trial of omicron XBB.1.5-containing bivalent mRNA vaccine. 2024;2(3):113-25.
556. Zhang S, Li J, Xu R, Chen Q, Sun G, Lin Y, et al. Safety of COVID-19 Vaccination in Patients With Breast Cancer: Cross-Sectional Study in China. JMIR PUBLIC HEALTH AND SURVEILLANCE. 2023;9.
557. Zhang Y, Ma X, Yan G, Wu Y, Chen Y, Zhou Z, et al. Immunogenicity, durability, and safety of an mRNA and three platform-based COVID-19 vaccines as a third dose following two doses of CoronaVac in China: A randomised, double-blinded, placebo-controlled phase 2 trial. ECLINICALMEDICINE. 2022;54.
558. Alves K, Kouassi A, Plested JS, Kalkeri R, Smith K, Kaba M, et al. Immunogenicity and safety of a monovalent Omicron XBB.1.5 SARS-CoV-2 recombinant spike protein vaccine in previously unvaccinated,

SARS-CoV-2 seropositive participants: Primary day-28 analysis of a phase 2/3 open-label study. VACCINE. 2025;55.
559. Diya O, Gayed J, Lowry FS, Ma H, Bangad V, Mensa F, et al. Safety and Immunogenicity of Monovalent Omicron KP.2-Adapted BNT162b2 COVID-19 Vaccine in Adults: Single-Arm Substudy from a Phase 2/3 Trial. Infect Dis Ther. 2025;14(8):1973-87.
560. Ferreira-da-Silva R, Lobo MF, Pereira AM, Morato M, Polónia JJ, Ribeiro-Vaz I. Network analysis of adverse event patterns following immunization with mRNA COVID-19 vaccines: real-world data from the European pharmacovigilance database EudraVigilance. FRONTIERS IN MEDICINE. 2025;12.
561. Fierro C, Sanchez-Crespo N, Makrinos D, Zhang WJ, Sun YB, Rohilla P, et al. Shared clinical and immunologic features of mRNA vaccines: preliminary results from a comparative clinical study. FRONTIERS IN IMMUNOLOGY. 2025;16.
562. Goswami J, Cardona JF, Hsu DC, Simorellis AK, Wilson L, Dhar R, et al. Safety and immunogenicity of mRNA-1345 RSV vaccine coadministered with an influenza or COVID-19 vaccine in adults aged 50 years or older: an observer-blinded, placebo controlled, randomised, phase 3 trial. LANCET INFECTIOUS DISEASES. 2025;25(4):411-23.
563. Ho NT, Smolenov I, Tran LTL, Nguyen V, Ta V, Nguyen TV, et al. Safety profile of self-amplifying mRNA SARS-CoV-2 vaccine ARCT-154 in adults: a pooled phase 1/2/3 randomized clinical study. EXPERT REVIEW OF VACCINES. 2025;24(1):299-312.
564. Jantarabenjakul W, Nantanee R, Puthanakit T, Gatechompol S, Avihingsanon A, Punrin S, et al. Immunogenicity and safety of 'Comvigen', a bivalent SARS-CoV-2 vaccine, in comparison to Comirnaty bivalent vaccine in Thailand: a phase 2, non-inferiority randomised trial. Lancet Reg Heal Southeast Asia. 2025;40.
565. Krusic A, Mencin N, Leban M, Nett E, Perkovic M, Sahin U, et al. Reverse-phase chromatography removes double-stranded RNA, fragments, and residual template to decrease immunogenicity and increase cell potency of mRNA and saRNA. MOLECULAR THERAPY NUCLEIC ACIDS. 2025;36(2).
566. Liu XQ, Qi YY, Huang T, Huang LL, Zhao MX, Wang ZF, et al. Efficacy, immunogenicity, and safety of heterologous boosting with a novel chimera Chinese mRNA (RQ3013) SARS-CoV-2 vaccine: A randomized, double-blind, active-controlled trial. HUMAN VACCINES & IMMUNOTHERAPEUTICS. 2025;21(1).
567. Maine CJ, Miyake-Stoner SJ, Spasova DS, Picarda G, Chou AC, Brand ED, et al. Safety and immunogenicity of an optimized self-replicating RNA platform for low dose or single dose vaccine applications: a randomized, open label Phase I study in healthy volunteers. NATURE COMMUNICATIONS. 2025;16(1).
568. Mazarakis N, Toh ZQ, Neal E, Bright K, Luu S, Quah L, et al. The immunogenicity, reactogenicity, and safety of a bivalent mRNA or protein COVID-19 vaccine given as a fourth dose. JOURNAL OF INFECTION. 2025;90(3).
569. Nurminen ML, Lindemo P, Sundström A, Zethelius B, Larsson M, Attelind S, et al. Spontaneous Reports of Adverse Reactions with Fatal Outcomes After COVID-19 Vaccination During the National Vaccination Campaign in Sweden. Clin Drug Invest. 2025;45(9):665-75.
570. Okada Y, Kumagai Y, Okura I, Otsuki M, Ishida N, Iwama Y, et al. Immunogenicity of a booster dose of a bivalent (Asp614Gly and omicron BA.4/5 variant) self-amplifying mRNA SARS-CoV-2 booster vaccine versus the BNT162b2 omicron BA.4/5 mRNA vaccine: a randomised phase 3 trial. LANCET INFECTIOUS DISEASES. 2025;25(3):290-300.
571. Ramgi P, Siribié M, Rakotozandrindrainy N, Bule O, Shrivastava H, Chambule L, et al. Immunogenicity and Safety of Heterologous Versus Homologous Prime-Boost Regimens With BBIBP-CorV and Ad26.COV2.S COVID-19 Vaccines: A Multicentric, Randomized, Observer-Blinded Non-inferiority Trial in Madagascar and Mozambique. Clin Infect Dis. 2025;80:S37-S46.
572. Rowe SL, Sullivan SG, Koerber JJ, Muňoz FM, Coates MM, Arah OA, et al. Adverse events associated with COVID-19 vaccination or diagnosis among pregnant and non-pregnant women in the United States, 2021-2022. Int J Infect Dis. 2025;160.
573. Thuluva S, Paradkar V, Gunneri S, Mogulla RR, Yerroju V, Dhar C, et al. Safety and immunogenicity of booster doses of an XBB.1.5 RBD subunit COVID-19 vaccine among individuals aged 5–80 years in India: a phase 3, single-blind, randomised controlled trial. Lancet Reg Heal Southeast Asia. 2025;40.
574. Komorowski AS, Mbuagbaw L, Jeyanathan M, Afkhami S, Swinton M, D'Agostino MR, Howie K, Medina MFC, Gauvreau GM, Brister DL, Satia I, Lichty BD, Miller MS, Cameron DW, Halperin S, Thabane L, Loeb M, Xing Z, Smaill FM. AeroVax: study protocol for a randomised, double-blind, placebo-controlled, phase 2 trial to evaluate safety and immunogenicity of a next-generation COVID-19 vaccine delivered by inhaled

aerosol to humans. ERJ Open Res. 2025 Jun 9;11(3):00758-2024. doi: 10.1183/23120541.00758-2024. PMID: 40491461; PMCID: PMC12147143.
575. Chaiwong W, Takheaw N, Laopajon W, Nisoong C, Pata S, Duangjit P, Inchai J, Pothirat C, Bumroongkit C, Deesomchok A, Theerakittikul T, Limsukon A, Tajarernmuang P, Niyatiwatchanchai N, Trongtrakul K, Kasinrerk W, Liwsrisakun C. Low-dose intradermal mRNA-1273 boosting vaccine following BBiBP-CorV vaccination during the omicron pandemics. Vaccine. 2025 Aug 13;61:127330. doi: 10.1016/j.vaccine.2025.127330. Epub 2025 Jun 4. PMID: 40472671.
576. Mayer EF, Falsey AR, Clark R, Ferguson M, Cardona J, She F, Jones B, Reuter C, Collins A, Mannan A, Kapoor A, Slobod K, Stoszek SK, Du J, Mou J, Lan L, Zhou H, Wilson E, Goswami J, Das R, Priddy F. Safety, Tolerability, and Immunogenicity of mRNA-1345 in Adults at Increased Risk for RSV Disease Aged 18 to 59 Years. Clin Infect Dis. 2025 Jun 4:ciaf292. doi: 10.1093/cid/ciaf292. Epub ahead of print. PMID: 40464662.
577. Palacka P, Marusakova E, Rejlekova K, Svobodova A, Skokanova L, Vulganova M, Obertova J. Humoral immune response following the third dose of BNT162b2 received by employees at a Slovak cancer healthcare facility. Biomed Rep. 2025 May 20;23(1):120. doi: 10.3892/br.2025.1998. PMID: 40463399; PMCID: PMC12131294.
578. Kawamoto A, Hashida M, Ishida K, Furihata K, Ota A, Takahashi K, Sakakibara S, Nakano T, Takeshita F. Immunogenicity and safety of DS-5670d, an omicron XBB.1.5-targeting COVID-19 mRNA vaccine: A phase 3, randomized, active-controlled study. PLoS Med. 2025 Oct 13;22(10):e1004499. doi: 10.1371/journal.pmed.1004499. PMID: 41082550; PMCID: PMC12517495.
579. van Beek LF, He X, Koks MS, van der Gaast-de Jongh CE, Roukens AHE, Visser LG, Diavatopoulos DA, de Graaf H, de Jonge MI. Comparing the immunogenicity of intradermal and intramuscular vaccination of elderly with BNT162b2 XBB.1.5: An equivalent dose study. Vaccine. 2025 Oct 3;64:127749. doi: 10.1016/j.vaccine.2025.127749. Epub 2025 Sep 15. PMID: 40957304.
580. Giles ML, Tabora C, Baccarini C, Barrientos L, Vargas JC, Montellano ME, Nguyen P, Deshmukh S, Neville M, Hohenboken M, van Boxmeer J, Jin H, Bugarini R, Liu X, Walson JL, Verhoeven C, Smolenov I. Immunogenicity and safety of self-amplifying mRNA COVID-19 vaccine (ARCT-2303), with or without co-administration of seasonal inactivated influenza vaccine in adults: a phase 3, randomised, controlled, observer-blind, multicentre study. EClinicalMedicine. 2025 Aug 20;87:103428. doi: 10.1016/j.eclinm.2025.103428. PMID: 40896462; PMCID: PMC12396474.
581. Bao Z, Gao W, Yu X, Chai L, Liu Y. Post-marketing safety monitoring of RSV vaccines: A real-world study based on the Vaccine Adverse Event Reporting System (VAERS). Hum Vaccin Immunother. 2025 Dec;21(1):2550857. doi: 10.1080/21645515.2025.2550857. Epub 2025 Aug 27. PMID: 40862314; PMCID: PMC12392792.
582. Loedy N, Dorta HG, Abrams S, Crèvecoeur J, Morales DR, Cohet C, Willem L, Molenberghs G, Hens N, Kurz X, Quinten C, Verbeeck J. An extension of the Benefit Risk Assessment of VaccinEs toolkit to evaluate Comirnaty and Spikevax vaccination in the European Union. Eur J Public Health. 2025 Oct 1;35(5):1007-1013. doi: 10.1093/eurpub/ckaf135. PMID: 40829167; PMCID: PMC12529280.
583. Hviid A, Svalgaard IB. Safety of BNT162b2 mRNA COVID-19 Vaccine Batches: A Nationwide Cohort Study. Pharmacoepidemiol Drug Saf. 2025 Sep;34(9):e70207. doi: 10.1002/pds.70207. PMID: 40814857; PMCID: PMC12355449.
584. Janani L, Munro APS, Wright A, Aley PK, Babbage G, Baxter D, Bawa T, Bibi S, Bula M, Cathie K, Chatterjee K, Cosgrove C, Enever Y, Galiza E, Goodman AL, Green CA, Harris M, Hicks A, Jones CE, Kanji N, van der Klaauw AA, Libri V, Llewelyn MJ, Mansfield R, McGregor AC, Minassian AM, Moore P, Mujadidi YF, Belhadef HT, Holliday K, Osanlou O, Osanlou R, Pacurar M, Palfreeman A, Regan K, Saich S, Saralaya D, Sharma S, Sheridan R, Stokes M, Thomson EC, Todd S, Twelves C, Wright D, Read RC, Charlton S, Hallis B, Ramsay M, Andrews N, Nguyen-Van-Tam JS, Cornelius V, Lambe T, Heath PT, Liu X, Faust SN; COV-BOOST study group. Heterologous COVID-19 vaccine schedule with protein-based prime (NVX-CoV2373) and mRNA boost (BNT162b2) induces strong humoral responses: Results from COV-BOOST trial. J Infect. 2025 Sep;91(3):106576. doi: 10.1016/j.jinf.2025.106576. Epub 2025 Aug 7. PMID: 40782888.
585. Wu S, Huang J, Wang B, Li J, Wu J, Zhang Z, Luo L, Zhang J, Huo N, Long J, Huang H, Chen Z, Zhang M, Zhao Z, Dan J, Song X, Mao H, Huo S, Yan H, Zhang Y, Wang X, Hou L. Safety and Immunogenicity of aerosolized adenovirus-vectored COVID-19 vaccine and intramuscular mRNA vaccine bivalent boosters: a randomized open-label clinical trial. Nat Commun. 2025 Aug 7;16(1):7281. doi: 10.1038/s41467-025-62698-7. PMID: 40775215; PMCID: PMC12332051.

586. Lidström AK, Albinsson B, Sund F, Lindbäck J, van Hunsel F, Fall T, Westman G. Adverse drug reactions following SARS-CoV-2 vaccination of 3805 healthcare workers cause substantial sick-leave and are correlated to vaccine regimen, age, sex and serological response. Vaccine. 2025 Aug 30;62:127553. doi: 10.1016/j.vaccine.2025.127553. Epub 2025 Aug 6. PMID: 40773962.
587. Kuriyama K, Murakami K, Sugiura K, Sakui S, Schuring RP, Mori M. One-year follow-up of the immunogenicity and safety of a first and second booster dose of the NVX-CoV2373 (TAK-019) vaccine in healthy Japanese adults who had previously received a primary series of COVID-19 mRNA vaccine: Final report of a phase 3 open-label trial. Vaccine. 2025 Aug 30;62:127562. doi: 10.1016/j.vaccine.2025.127562. Epub 2025 Aug 5. PMID: 40753673.
588. Bawazir WM, Ibad AA, Mohsin M, Niyazi HA, Alamri TA, Bazuhair MA, Hazzazi M, Chehab NA, Harakeh S, Yousafzai YM. Immune and hematologicak responses to the third dose of an mRNA COVID-19 vaccine: a six-month longitudinal study. Front Cell Infect Microbiol. 2025 Jul 10;15:1615227. doi: 10.3389/fcimb.2025.1615227. PMID: 40708749; PMCID: PMC12286925.
589. Pan Y, Han Y, Zhou C, Zhao L, Zheng J, Ye X, He Y; N3C Consortium. Evaluating the safety of XBB.1.5-containing COVID-19 mRNA vaccines using a self-controlled case series study. Nat Commun. 2025 Jul 15;16(1):6514. doi: 10.1038/s41467-025-61613-4. PMID: 40664645; PMCID: PMC12264068.
590. Chalkias S, Dennis P, Petersen D, Radhakrishnan K, Vaughan L, Handforth R, Rossi A, Wahid R, Edwards DK, Feng J, Deng W, Zhou H, De Windt E, Urdaneta V, Paila Y, Girard B, Faust SN, Walsh SR, Cosgrove CA, Miller J, Das R. Efficacy, immunogenicity, and safety of a next-generation mRNA-1283 COVID-19 vaccine compared with the mRNA-1273 vaccine (NextCOVE): results from a phase 3, randomised, observer-blind, active-controlled trial. Lancet Infect Dis. 2025 Nov;25(11):1230-1242. doi: 10.1016/S1473-3099(25)00236-1. Epub 2025 Jul 7. PMID: 40639387.
591. Prabhu R, Prabhu G, Htay MNN, Thundakattil AV, Moe S, Das S. Safety comparison of mRNA, viral vector, and inactivated Covid-19 vaccines: incidence of adverse events following primary and booster doses among medical professionals in Malaysia. BMC Infect Dis. 2025 Jul 7;25(1):898. doi: 10.1186/s12879-025-11254-1. PMID: 40624462; PMCID: PMC12232647.
592. Kim R, Uhm TG, Kim J, Woo D, Kim UI, Meng X, Yang B, Kim S, Kim H, Kim J, Yoon S, Lee JY, Kim B, Cho D, Chang D, Cho YH, Choi K, Gwak W, Lee HW, Bang J, Hellström E, Kim B, Kim K, Yang JS. A novel and safe SmartCap® SC101 to develop the COVID-19 mRNA vaccine STP2104 inducing potent immune responses in humans. Front Immunol. 2025 Jun 16;16:1571092. doi: 10.3389/fimmu.2025.1571092. PMID: 40589754; PMCID: PMC12206861.
593. Kitonsa J, Serwanga J, Cheeseman HM, Abaasa A, Lunkuse JF, Ruzagira E, Kato L, Nambaziira F, Oluka GK, Gombe B, Jackson S, Ssebwana JK, McFarlane LR, Joseph S, Pierce BF, Shattock RJ, Kaleebu P. Safety and Immunogenicity of a Modified Self-Amplifying Ribonucleic Acid (saRNA) Vaccine Encoding SARS-CoV-2 Spike Glycoprotein in SARS-CoV-2 Seronegative and Seropositive Ugandan Individuals. Vaccines (Basel). 2025 May 23;13(6):553. doi: 10.3390/vaccines13060553. PMID: 40573884; PMCID: PMC12197785.
594. Konishi A, Fukushima W, Matsuura T, Ohfuji S, Kase T, Kondo K, Matsumoto K, Suita A, Mukai E, Kasamatsu A, Kido Y, Nakagama Y, Nitahara Y, Kaneko Y, Kaneko A, Nakagami-Yamaguchi E, Kakeya H, Hirota Y. Adverse reactions following first three doses of the BNT162b2 mRNA COVID-19 vaccine: A prospective cohort study on relationships with individual characteristics and previous experience of adverse reactions. Hum Vaccin Immunother. 2025 Dec;21(1):2518646. doi: 10.1080/21645515.2025.2518646. Epub 2025 Jun 24. PMID: 40555671.
595. Stefanizzi P, Moscara L, Palmieri C, Martinelli A, Fontanelli S, Bellomo C, Spinelli G, Tafuri S. Safety of SARS-CoV-2 XBB.1.5 and seasonal influenza vaccines co-administration: data from a perspective observational active surveillance study. Puglia (Italy), season 2023/2024. Virology. 2025 Sep;610:110613. doi: 10.1016/j.virol.2025.110613. Epub 2025 Jun 20. PMID: 40561867.
596. Vacharathit V, Pluempreecha M, Manopwisedjaroen S, Srisaowakarn C, Srichatrapimuk S, Sritipsukho P, Sritipsukho N, Thitithanyanont A. Persistent IP-10/CXCL10 dysregulation following mild Omicron breakthrough infection: Immune network signatures across COVID-19 waves and implications for mRNA vaccine outcomes. Clin Immunol. 2025 Sep;278:110507. doi: 10.1016/j.clim.2025.110507. Epub 2025 Apr 28. PMID: 40306350.
597. Covid-19 Vaccine Boosting in a Real World Setting: Exploratory Phase 3, Cohort Randomized, Single-blind, Multi-center Study to Evaluate the Safety and Immunogenicity of a Booster Dose of Various Covid-19 Vaccines in Fully Covid-19 Vaccine-primed Subjects Regardless of the Number of Prior Booster2023 2023.

598. Immunogenicity and Safety of Concomitant Administration of Omicron-containing Bivalent COVID-19 Vaccines With Seasonal Influenza Vaccines2023 2023.
599. Analysis of Immunization Time, Amplitude and Adverse Events of 7 Different Vaccines Against SARS-COV-2 Across 4 Countries2022 2022.
600. Establishing Immunogenicity and Safety of Needle-free Intradermal Delivery by Solid Micro Needle Skin Patch of mRNA SARS-CoV-2 Vaccine as a Revaccination Strategy in Healthy Volunteers2022 2022.
601. Randomized Trial of COVID-19 Booster Vaccinations (Cobovax Study)2021 2021.
602. Immunogenicity and Reactogenicity Following a 3rd Dose of COVID-19 mRNA Vaccine (Pfizer-BioNtech) and Two Adjuvanted Sub-unit Vaccines (SP/GSK) Administered as a Booster in Adults Who Received 2 Doses of Pfizer-BioNTech mRNA Vaccine as a Primary Vaccination: A Randomized, Single-blinded Multicent2021 2021.
603. Canetti, Michal et al. "Immunogenicity and efficacy of fourth BNT162b2 and mRNA1273 COVID-19 vaccine doses; three months follow-up." Nature communications vol. 13,1 7711. 13 Dec. 2022, doi:10.1038/s41467-022-35480-2
604. Stærke NB, Reekie J, Johansen IS, Nielsen H, Benfield T, Wiese L, Søgaard OS, Tolstrup M, Iversen KK, Tarp B, Larsen FD, Larsen L, Lindvig SO, Holden IK, Iversen MB, Knudsen LS, Fogh K, Jakobsen ML, Traytel AK, Ostergaard L, Lundgren J; ENFORCE Study Group. Cohort Profile:The Danish National Cohort Study of Effectiveness and Safety of SARS-CoV-2 vaccines (ENFORCE). BMJ Open. 2022 Dec 30;12(12):e069065. doi: 10.1136/bmjopen-2022-069065. PMID: 36585137; PMCID: PMC9809224.
605. Abdel-Qader DH, Abdel-Qader H, Silverthorne J, Kongkaew C, Al Meslamani AZ, Hayajneh W, et al. Active Safety Surveillance of Four Types of COVID-19 Vaccines: A National Study from Jordan. 2022;42(10):813-27.
606. Salter S, M. r, Li D, Trentino K, Nissen L, Lee K, et al. Safety of Four COVID-19 Vaccines across Primary Doses 1, 2, 3 and Booster: A Prospective Cohort Study of Australian Community Pharmacy Vaccinations. VACCINES. 2022;10(12). (unclear intervention-outcome link)

607. A Parallel Group, Prospective, Randomized, Double-blind, Two-arm, Single-center Study to Evaluate the Immunogenicity, Safety, and Tolerability of mRNA-1273 in Heterologous Prime-Boost With MVC-COV1901 in Adult Volunteers of 20 to 70 Years [Internet]. 2021. Available from: https://clinicaltrials.gov/study/NCT05079633.
608. Fourth BNT162b2 COVID-19 Vaccine Dose - the Sheba HCW Cohort [Internet]. 2022. Available from: https://clinicaltrials.gov/study/NCT05231005.
609. A Phase 3b/4, Open-label Study to Assess the Immunogenicity and Safety of mRNA COVID-19 Variant-containing Vaccine Formulations [Internet]. 2024. Available from: https://clinicaltrials.gov/study/NCT06585241.
610. Asomaning K, Duh MS, DerSarkissian M, de Luise C, Nguyen C, Cheng M, et al. Post-emergency use authorization active safety surveillance study of the Pfizer-BioNTech coronavirus disease 2019 (COVID-19) vaccine in the Veterans Affairs health system: Findings from a three-year, comprehensive safety assessment program. Vaccine: X. 2026;29.
611. Escolano S, Quentin E, Demailly R, Ahmed I, Tubert-Bitter P. Background incidence rates and observed-to-expected ratios of adverse events of special interest after covid-19 mRNA vaccination during pregnancy in France: a nationwide population-based study. The Lancet regional health Europe. 2026;64:101630.
612. Figueroa AL, Johnson K, Springer R, Lowe J, Cripple A, Edwards DK, et al. Immunogenicity and safety of LP.8.1 variant-containing mRNA COVID-19 vaccines. medRxiv. S. Chalkias, Moderna, Inc., 325 Binney Street, Cambridge, MA, United States2026.
613. Hansen CH, Lassaunière R, Rasmussen M, Moustsen-Helms IR, Valentiner-Branth P. Effectiveness of the BNT162b2 and mRNA-1273 JN.1-adapted vaccines against COVID-19-associated hospitalisation and death: a Danish, nationwide, register-based, cohort study. LANCET INFECTIOUS DISEASES. 2025;25(12):1293-302.
614. He AN, Hur K, Li XX, Zheng J, Hur J, He YQ. Updated profiling of COVID-19 vaccine adverse events using VAERS case reports. FRONTIERS IN PHARMACOLOGY. 2026;17.
615. Ishiguro C, Mimura W, Oda F, Maeda M, Fukuda H. Population-based cohort study assessing the effectiveness of maternal BNT162b2/mRNA-1273 vaccination against infant coronavirus disease 2019 outcomes during Omicron predominance: The VENUS study. Human vaccines & immunotherapeutics. 2025;21(1):2585595.
616. Ljung R, Pihlström N, Dahl J, Tapia G, Sundström A, Nurminen ML, et al. SARS-CoV-2 vaccination and myositis in Norway and Sweden. Rheumatology. 2026;65(2).

617. Rodríguez-Vidales EP, Santos-Flores JM, Garza-Rodríguez MI, Salinas-Martínez AM, Martínez-Pérez AG, Montes de Oca-Luna R, et al. Sex, Age, and COVID-19 Vaccine Characteristics Associated with Adverse Events After Vaccination and Severity: A Retrospective Analysis. Infect Dis Rep. 2025;17(5).
618. Zidan M, Trinh NTH, Desalegn A, Smith LH, van Gelder M, Nordeng H, et al. BNT162b2 mRNA COVID-19 vaccine effectiveness in pregnancy: Emulating trial NCT04754594 using observational data from Norwegian health registries. Vaccine. 2025;68:127908.
619. A Phase I/II Clinical Trial to Evaluate the Safety, Immunogenicity and Immune Persistence of COVID-19 mRNA Vaccine in Healthy People Aged 18 Years and Above2021 2021.

Ongoing trials (n = 6)

1. A Randomized, Single-blind, Parallel Controlled Trial to Evaluate the Immunogenicity ,Safety,Efficacy of A Heterologous Booster Dose With SW-BIC-213, in Previously Vaccinated Subjects Against COVID-19 With Two Inactivated COVID-19 Vaccine Doses Compared to a Booster Dose With Pfizer of Sinopharmi2022 2022.
2. A Phase 2 Randomized, Active-controlled, Observer-blind Study to Assess the Safety, Reactogenicity, and Immunogenicity of a Booster Dose of Investigational COVID-19 mRNA Vaccines in Healthy Adults Who Previously Received a Complete Primary Vaccination Series With or Without Booster Dose(s)2023 2023.
3. A Phase 2/3, Randomized, Double-blinded , Parallel Controlled Trial to Evaluate the Immunogenicity, Safety and Efficacy of A Heterologous Booster Dose With COVID-19 mRNA Vaccines (Bivalent) , in Previously Vaccinated Subjects Against COVID-19 With 2/3 Doses COVID-19 Vaccine Compared to a Booster2023 2023.
4. A Phase 1/2 Multicenter, Randomized Study of the Safety and Immunogenicity of a Sub-unit Protein CD40.RBDv Bivalent COVID-19 Vaccine, Adjuvanted or Not, as a Booster in Volunteers2024 2024.
5. A Phase 4, Randomized, Observer-Blind, Placebo-Controlled Study to Evaluate the Efficacy and Safety of mRNA-1283 And mRNA-1273 Variant-Containing Formulation in Adults 50 to 64 Years of Age Without Underlying Conditions That Put Them at High Risk for Severe Outcomes From COVID-19 [Internet]. 2025. Available from: https://clinicaltrials.gov/study/NCT07266558.

6. National Cohort Study of Effectiveness and Safety of SARS-CoV-2/COVID-19 Vaccines [Internet]. 2021. Available from: https://clinicaltrials.gov/study/NCT04760132.

Full text not retrievable (n = 1)

1. Argirò C, Costantino L, Morrillo R, Salerno V, Daniele E, Saullo F. Safety monitoring of anti-COVID-19 vaccines and the key role of the Hospital Pharmacist during the vaccination campaign. 2023;37(2):46-54.
